# Supplementary material for: Breastfeeding may lessen socioeconomic disparities in child health through differences in the infant gut microbiome
Source: Cell Rep Med. 2026 Apr 13;7(4):102755. doi: 10.1016/j.xcrm.2026.102755 (PMC13130633; doi:10.1016/j.xcrm.2026.102755)
Supplement: Document S1. Figures S1–S6 and Tables S1–S5 [file mmc1.pdf]

Cell Reports Medicine, Volume *7*

Supplemental information

Breastfeeding may lessen socioeconomic disparities in child health through differences in the infant gut microbiome

Darlene L.Y. Dai, Melissa B. Manus, Courtney Hoskinson, Jie Jiang, Hind Sbihi, Kozeta Miliku, Susan C. Campisi, Daphne J. Korczak, Qingling Duan, Theo J. Moraes, Piushkumar J. Mandhane, B. Brett Finlay, Elinor Simons, Hannah Lishman, David M. Patrick, Padmaja Subbarao, Meghan B. Azad, Bo Chawes, Klaus Bønnelykke, Søren Johannes Sørensen, Jonathan Thorsen, Jakob Stokholm, Charisse Petersen, and Stuart E. Turvey

Supplemental Figures and Tables

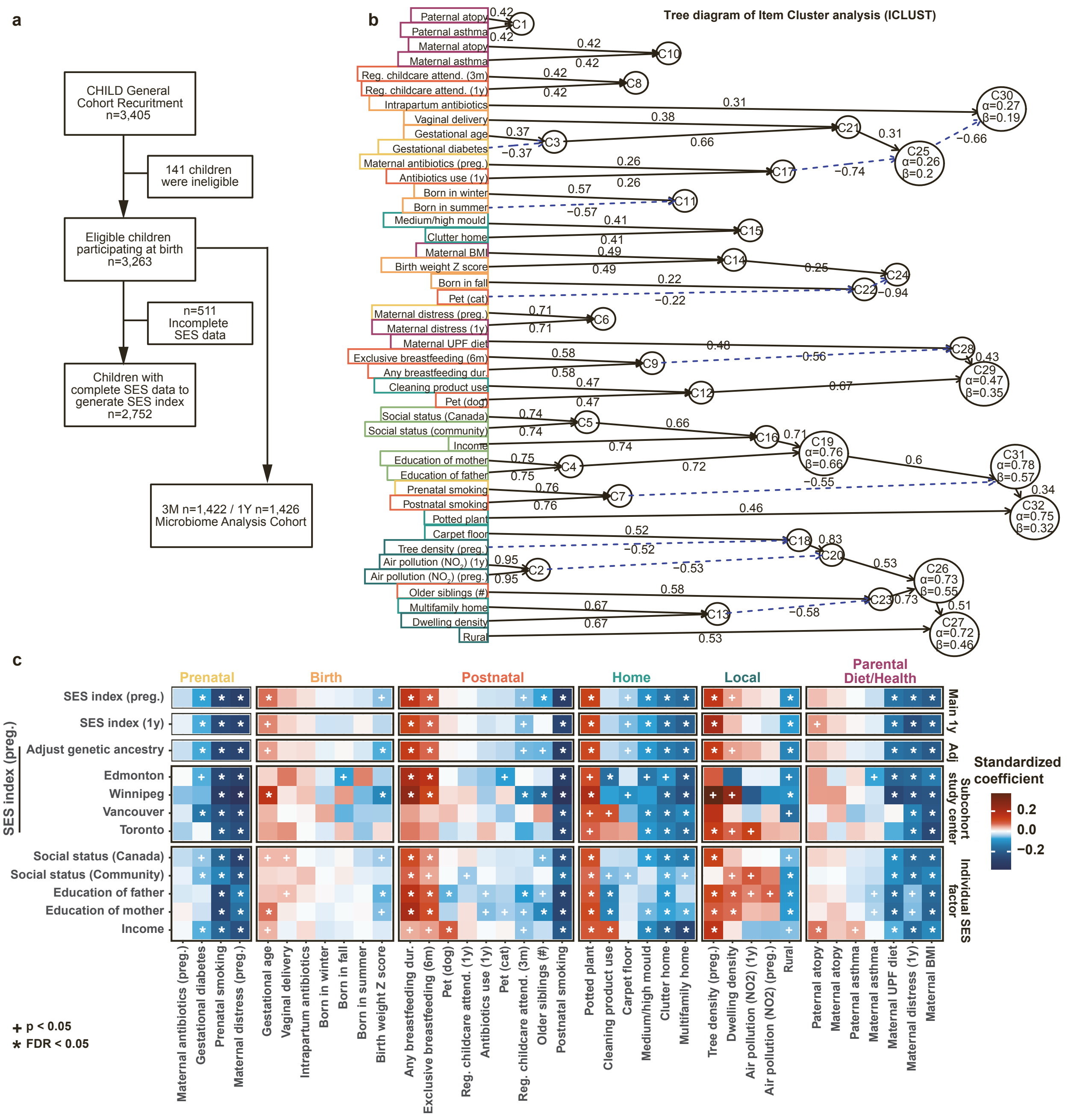

Fig. S1. Associations across SES, perinatal factors, and childhood health outcomes, Related to Figure 1. (a) Consort diagram of CHILD Cohort. (b) Tree diagram of the item cluster analysis (ICLUST) to cluster observed SES factors and perinatal factors and exposomes based on their Pearson correlations. Cluster 19 (C19) was found to include the 5 observed SES factors with Cronbach's alpha values >0.7, indicating internal consistency within the calculated clusters. (c) Heatmap of the associations between summarized SES index at enrollment and perinatal factors, and sensitivity analysis using summarized SES index based on observed SES factors collected at 1 year, with adjustment of genetic ancestry, sub-cohort analyses for each study center and individual observed SES factors using regression models. Red represents positive association and blue represents negative associations with + indicating $p<0.05$ and * indicating FDR<0.05. Factors are ordered by hierarchical clustering.

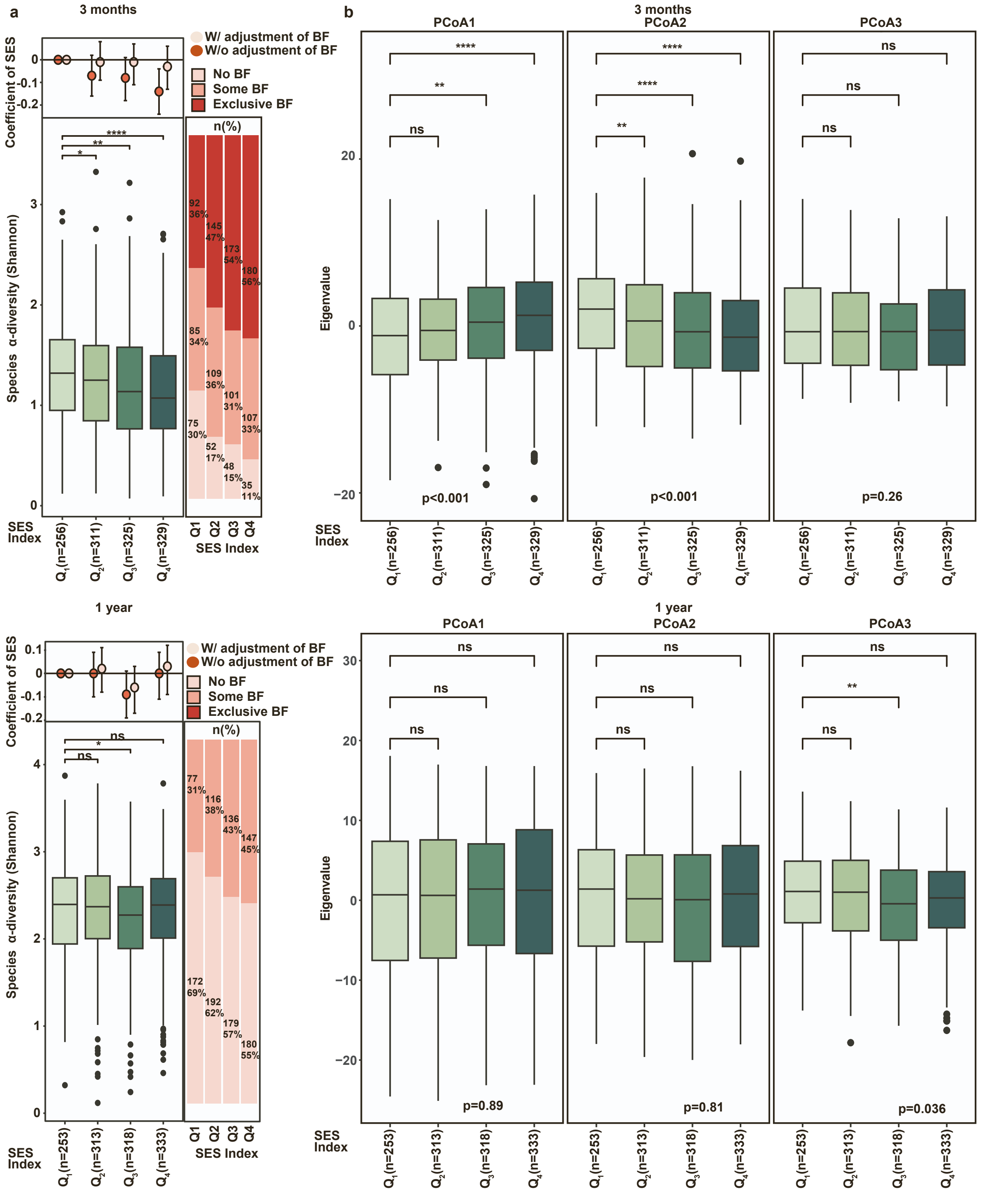

Fig. S2. SES is associated with infant microbiome composition at 3 month and 1 year, Related to Figure 2. (a) Differences in gut microbiota species α-diversity (Shannon index) at 3 months and 1 year across children with different quartiles of SES index. SES Q1 includes samples with the lowest 25% SES index and SES Q4 includes samples with the top 25% SES index. The right-side stacked bar plots showed the proportion of some (dark pink) and exclusive (orange) breastfeeding across SES quartiles. The top panels show the associations between species α-diversity and SES quartiles using linear regression models with (pink) and without (orange) adjustment of breastfeeding. Linear

regression models were all adjusted for stool sample collection age and processing time and with study site as a random effect. (b) Boxplot of the top 3 PCoAs across SES quartiles. Differences across SES quartiles compared to the first quartile of SES were estimated based on Wilcoxon test. ns $p>0.05$, * $p<0.05$, ** $p<0.01$, and *** $p<0.001$. The box displays 25th, 50th (median), and 75th percentiles, with whiskers extending 1.5 * IQR. P-values at the bottom of each figure were based on linear regression models using the continuous SES index with study site as a random effect and adjustment of stool sample collection age and processing time.

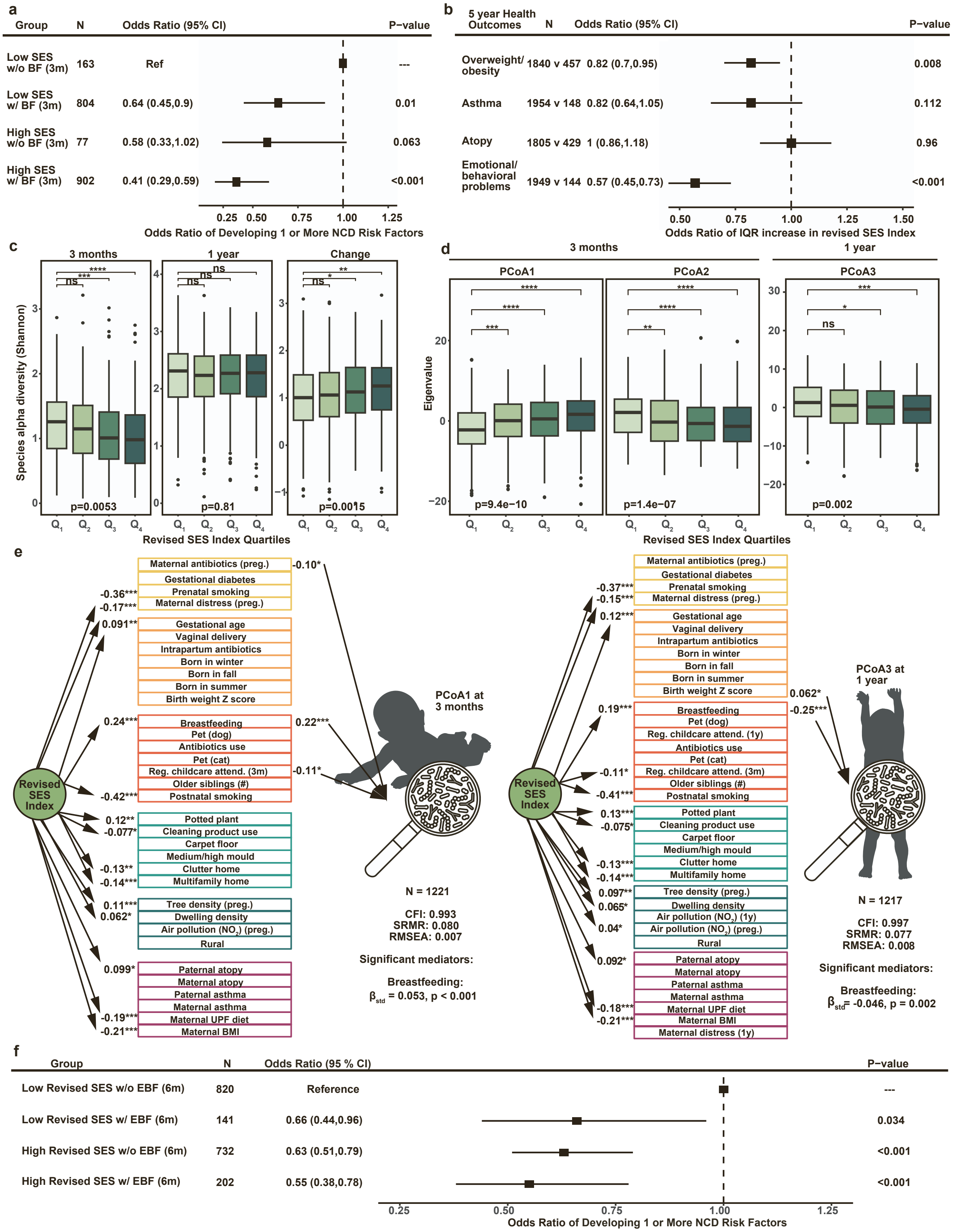

Fig. S3. Sensitivity analyses in CHILD Cohort examining any breastfeeding up to 3 months and the revised SES index, Related to Figure 2 and 3. (a) Forest plot of odds ratio (95% CI) for compound SES and any breastfeeding status (BF) at 3 months on the presence of one or more SES-associated NCD risk factor at age 5 (childhood

overweight/obesity, asthma and emotional/behavioral problems). (b) Forest plot of odds ratio (95% CI) for the association between childhood NCD risk factors at 5 years of age and an interquartile increase in the revised SES index, which is derived using only the three indicators applied in COPSAC (income and highest parental education). (c) Gut microbiota α-diversity (Shannon index) at 3 months, 1 year, and the change between these ages, and the top three significant PCoA axes (d) across the revised SES quartiles (Q_1 = lowest 25%, Q_4 = highest 25 %). Wilcoxon tests compare each quartile to Q_1. P-values shown beneath each panel are from regression models using the continuous revised SES index with study site as a random effect and adjustment for stool collection age and processing time. ns $p>0.05$, * $p<0.05$, ** $p<0.01$, *** $p<0.001$. (e) SEM models linking the revised SES index, perinatal factors and exposomes, and infant gut microbiota (PCoA1 at 3 months on left; PCoA3 at 1 year on right). Only significant indirect effects and associations (one-headed arrows; $p<0.05$) are shown. (f) Forest plot of odds ratio (95% CI) for the revised SES index and exclusive breastfeeding status (EBF) at 6 months on the presence of one or more SES-associated NCD risk factor at age 5 (childhood overweight/obesity, asthma and emotional/behavioral problems).

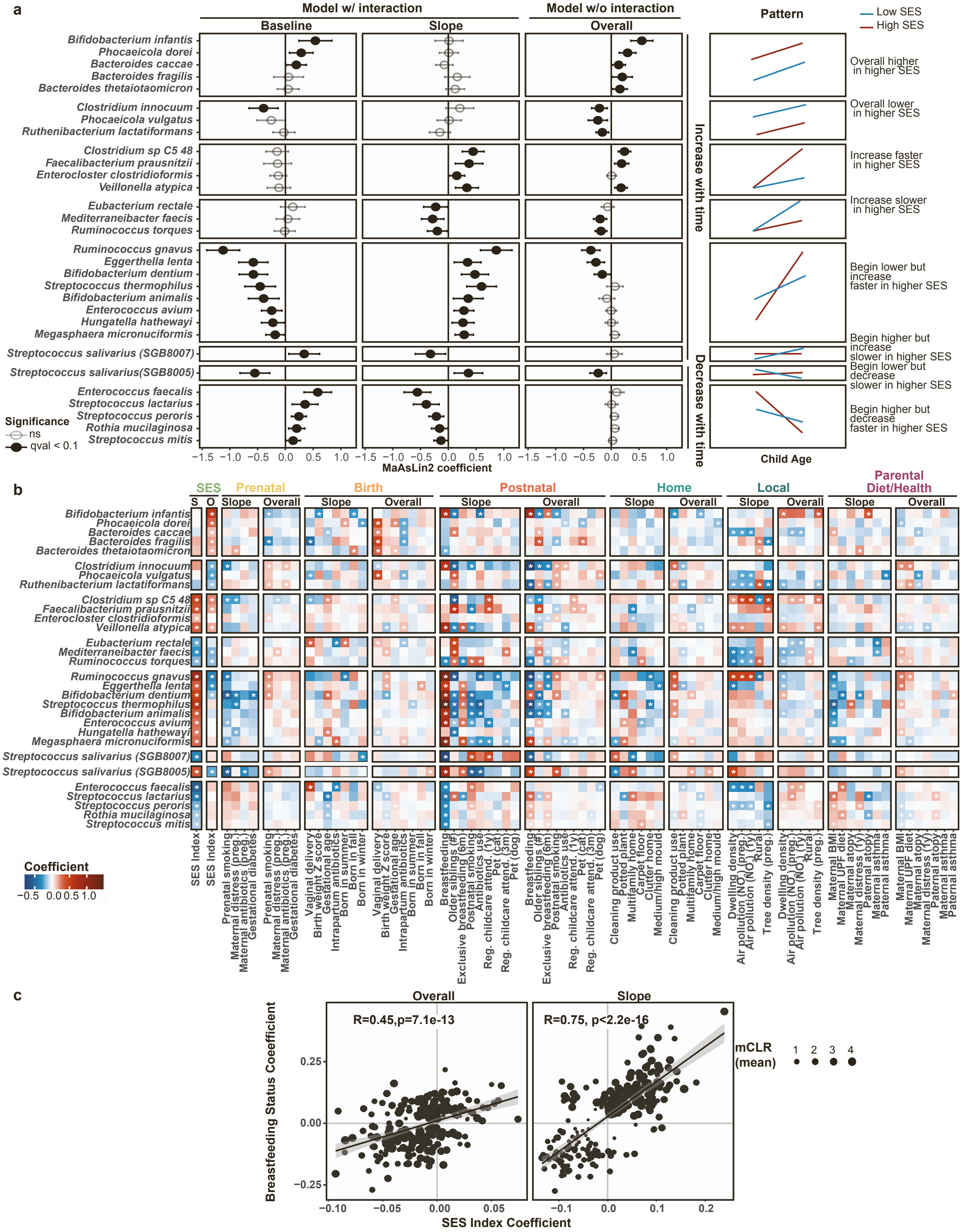

Fig. S4. Species and Metacyc pathways associated with SES, breastfeeding, and perinatal factors and exposomes, Related to Figure 5. (a) Baseline and slope effect (model with interaction) and overall effect (model without interaction) of SES index for species significantly associated with SES (FDR of slope effect or overall effect

< 0.1) across 3 months to 1 year based on MaAslin2 models. Data were presented as standardized coefficients of SES ± 1.96*standard error of the mean. The right-side panel summarizes the pattern of SES and time effect on each species across child age, in which the red line represents higher SES and the blue line represents lower SES and there is an interaction if the slope effect is significant. (b) Heatmap of the standardized slope and overall effect of perinatal factors on SES-associated species based on MaAslin2 models (FDR <0.1). Red represents positive association, while blue represents negative association. Stars represent significant associations (FDR < 0.1). (c) Correlation plot of overall effect (MaAslin2 models without interaction measuring overall colonization) and slope effect (MaAslin2 models with interaction measuring change over time) between SES and breastfeeding for Metacyc pathways significantly associated with either SES or breastfeeding (FDR of slope or overall effect < 0.1). Dot size represents the mean of relative abundance of Metacyc pathways across 3 months and 1 year. All MaAslin2 models used subject ID and study center as random effects and were adjusted for stool sample collection age and processing time.

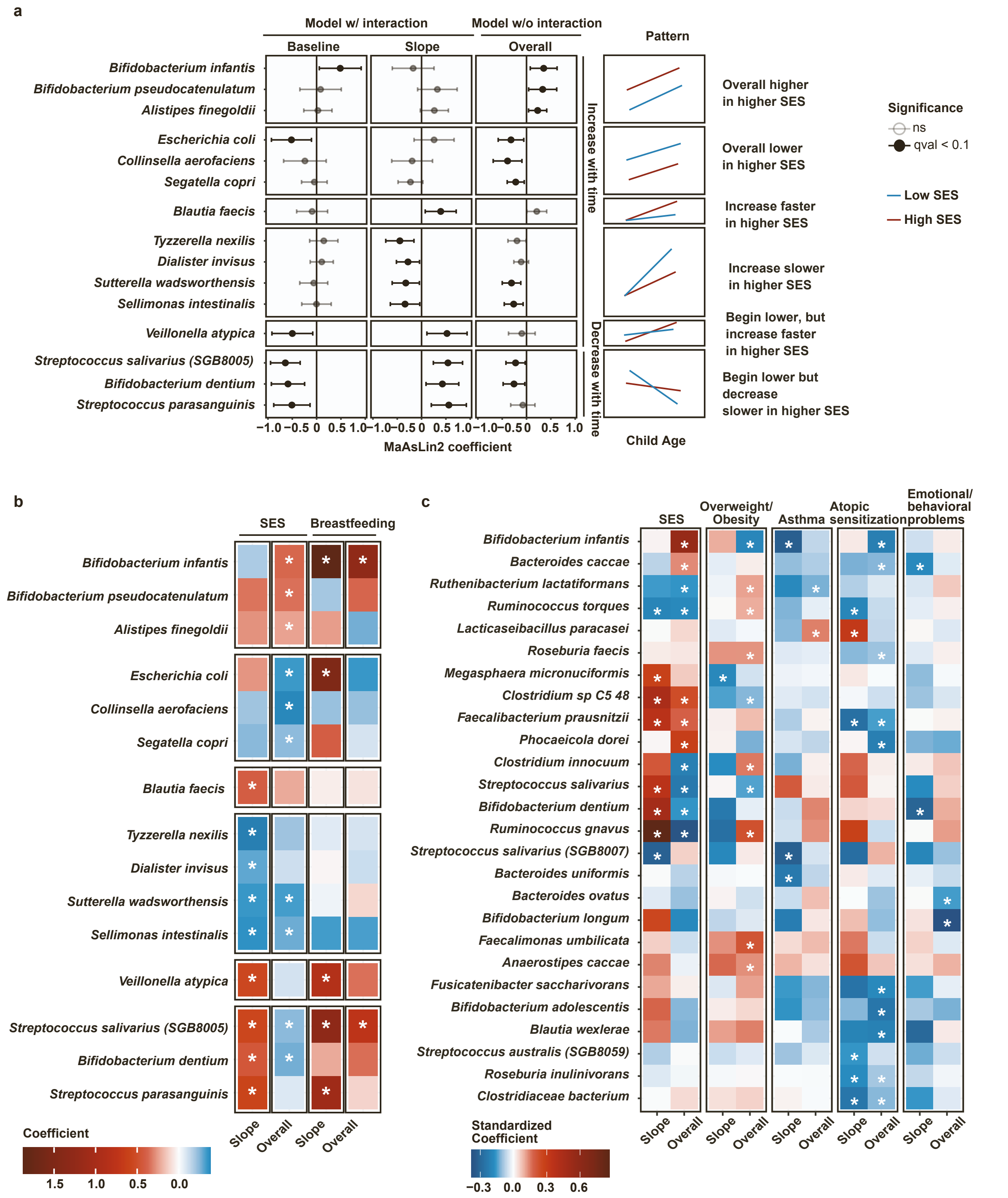

Fig. S5. Species significantly associated with SES and breastfeeding in COPSAC$_{2010}$ cohort, Related to Figure 4 and 6. (a) Baseline and slope effect (model with interaction) and overall effect (model without interaction) of SES index for species significantly associated with COPSAC$_{2010}$ SES index (FDR of slope effect or overall effect < 0.1)

across 1 month to 1 year based on MaAslin2 models. Data were presented as standardized coefficients of SES ± 1.96*standard error of the mean. The right-side panel summarizes the pattern of SES and time effect on each species across child age, in which the red line represents higher SES and the blue line represents lower SES and there is an interaction if the slope effect is significant. (b) Heatmap of the standardized slope and overall effect of SES and breastfeeding on SES-associated species based on MaAslin2 models (FDR <0.1). Red represents positive association, while blue represents negative association. Stars represent significant associations (FDR < 0.1). (c) Heatmap of standardized coefficient of SES index and childhood NCD risk factors for species significantly associated with at least one of four childhood NCD risk factors (FDR < 0.1) using MaAslin2 models with (slope effect) and without (overall effect) interaction in CHILD Cohort. All MaAslin2 models used subject ID and study center as random effects and were adjusted for stool sample collection age and processing time. Red represents positive association, while blue represents negative association. Stars represent FDR < 0.1.

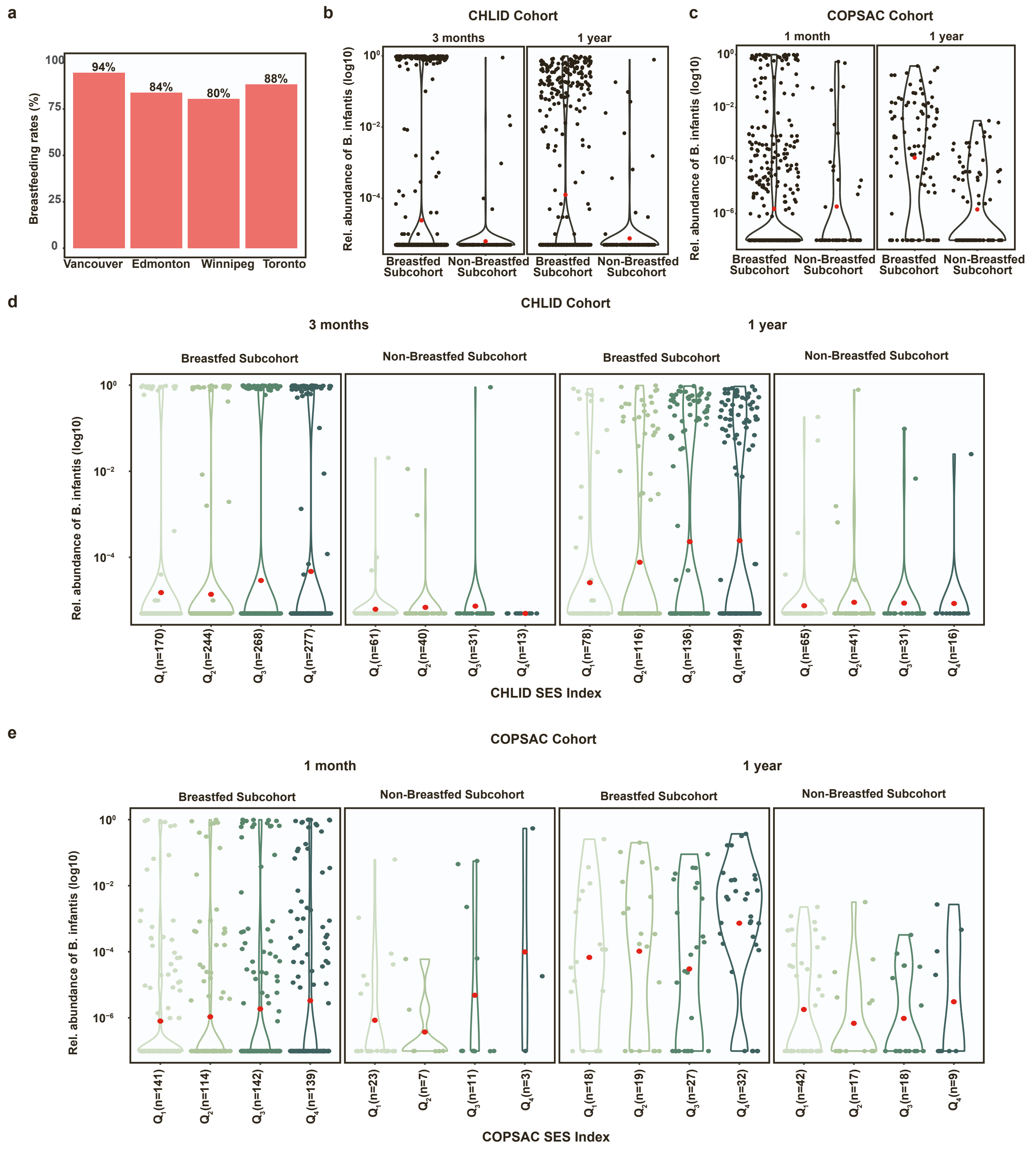

Fig S6. Breastfeeding rate in CHILD cohort and *B. infantis* abundance in COPSAC$_{2010}$ cohort, Related to Figure 6. (a) Breastfeeding rate (some breastfeeding at least 3 months) across CHILD study sites. (b-c) Violin plot of log 10 transformed relative abundance of *B. infantis* in Breastfed subcohort children and Non-Breastfed subcohort children in CHILD cohort (b) and COPSAC$_{2010}$ cohort (c). (d-e) Violin plot of log 10 transformed relative abundance of *B. infantis* in Breastfed and Non-Breastfed subcohort children at each visit in CHILD cohort (3-month visit and 1-year visit) (d) and COPSAC$_{2010}$ cohort (1-month visit and 1-year visit) (e). Red dot represents the mean.

Table S1. Characterizations of CHILD samples and across SES quartiles, Related to STAR Methods.

Category	Variable	Cohort	SES Index Q_1 (0-25%)	SES Index Q_2 (25-50%)	SES Index Q_3 (50-75%)	SES Index Q_4 (75-100%)
No. patients		3263	691	690	684	687
Confounder	Male, n(%)					
		1717 (52.6%)	350 (50.7%)	361 (52.3%)	374 (54.7%)	368 (53.6%)
	Study center, n(%)					
	Edmonton	768 (23.5%)	181 (26.2%)	206 (29.9%)	169 (24.7%)	104 (15.1%)
	Toronto	768 (23.5%)	71 (10.3%)	119 (17.2%)	162 (23.7%)	284 (41.3%)
	Vancouver	735 (22.5%)	109 (15.8%)	150 (21.7%)	199 (29.1%)	212 (30.9%)
	Winnipeg	992 (30.4%)	330 (47.8%)	215 (31.2%)	154 (22.5%)	87 (12.7%)
Health	Atopic sensitization at 5y, n(%)					
		494 (19.3%)	82 (15.2%)	107 (18.9%)	119 (21.4%)	113 (21.4%)
	Unknown	708 (21.7%)	151 (21.9%)	125 (18.1%)	128 (18.7%)	160 (23.3%)
	Asthma at 5y, n(%)					
		165 (6.9%)	37 (7.5%)	34 (6.5%)	44 (8.3%)	28 (5.4%)
	Unknown	864 (26.5%)	199 (28.8%)	167 (24.2%)	154 (22.5%)	171 (24.9%)
	Overweight/obese at 5y, n(%)					
		542 (20.6%)	125 (22.8%)	152 (26.2%)	92 (16.1%)	81 (14.7%)
	Unknown	638 (19.6%)	142 (20.5%)	109 (15.8%)	113 (16.5%)	137 (19.9%)
	Emotional/behavioral problems at 5y, n(%)					
		172 (7.3%)	49 (10.3%)	42 (7.8%)	24 (4.7%)	27 (5.1%)
	Unknown	903 (27.7%)	215 (31.1%)	153 (22.2%)	168 (24.6%)	159 (23.1%)
Pregnancy	Maternal antibiotics (preg.), n(%)					
		314 (9.6%)	81 (11.7%)	75 (10.9%)	61 (8.9%)	61 (8.9%)
	Gestational diabetes, n(%)					
		154 (4.7%)	52 (7.5%)	39 (5.7%)	19 (2.8%)	22 (3.2%)
	Maternal distress (preg.)					
	Median (Range)	13 (0, 35)	16 (0, 35)	13 (0, 33)	11 (0, 30)	10.5 (0, 34)
	IQR (Q1,Q3)	9, 17	11, 20	9, 17	7, 16	7, 14.8
	Unknown	325 (10%)	57 (8.2%)	48 (7%)	53 (7.7%)	33 (4.8%)
	Prenatal smoking, n(%)					
		606 (19.1%)	242 (35.3%)	125 (18.2%)	84 (12.3%)	50 (7.3%)
	Unknown	97 (3%)	5 (0.7%)	2 (0.3%)	2 (0.3%)	2 (0.3%)
Birth	Intrapartum antibiotics, n(%)					
		1326 (41.2%)	124 (24.1%)	135 (27%)	143 (28.2%)	137 (26.9%)
	Unknown	45 (1.4%)	176 (25.5%)	190 (27.5%)	177 (25.9%)	178 (25.9%)
	Delivery mode, n(%)					
	Vaginal	2412 (74.8%)	515 (76%)	502 (73.5%)	508 (74.9%)	511 (75.3%)
	C-section with labor	425 (13.2%)	88 (13%)	101 (14.8%)	93 (13.7%)	80 (11.8%)
	C-section without labor	387 (12%)	75 (11.1%)	80 (11.7%)	77 (11.4%)	88 (13%)
	Unknown	39 (1.2%)	13 (1.9%)	7 (1%)	6 (0.9%)	8 (1.2%)
	Birth weight Z score					
	Median (Range)	-0.1 (-3.1, 4.3)	0 (-2.9, 3.9)	-0.1 (-2.5, 4.3)	-0.2 (-2.6, 3.3)	-0.1 (-3.1, 2.8)
	IQR (Q1,Q3)	-0.7, 0.5	-0.6, 0.6	-0.7, 0.6	-0.7, 0.5	-0.7, 0.4
	Unknown	75 (2.3%)	20 (2.9%)	17 (2.5%)	13 (1.9%)	17 (2.5%)
	Gestational age					
	Median (Range)	278 (238, 300)	277 (240, 299)	278 (240, 300)	278 (241, 296)	278 (240, 295)
	IQR (Q1,Q3)	272, 283	271, 282	272, 283	272, 284	272, 284
	Unknown	54 (1.7%)	16 (2.3%)	10 (1.4%)	8 (1.2%)	12 (1.7%)
	Season of birth, n(%)					
	Spring	889 (27.2%)	181 (26.2%)	173 (25.1%)	202 (29.5%)	204 (29.7%)
	Summer	830 (25.4%)	168 (24.3%)	204 (29.6%)	148 (21.6%)	175 (25.5%)
	Fall	754 (23.1%)	167 (24.2%)	166 (24.1%)	164 (24%)	150 (21.8%)
	Winter	790 (24.2%)	175 (25.3%)	147 (21.3%)	170 (24.9%)	158 (23%)
Postnatal	Antibiotics use (1y), n(%)					
		619 (26.1%)	137 (28.2%)	128 (24.5%)	128 (23.8%)	124 (24.3%)
	Unknown	890 (27.3%)	206 (29.8%)	168 (24.3%)	146 (21.3%)	176 (25.6%)
	Reg. childcare attend. (1y), n(%)					
		735 (28.5%)	150 (28.4%)	165 (28.4%)	175 (31.2%)	152 (27.3%)
	Unknown	688 (21.1%)	163 (23.6%)	110 (15.9%)	123 (18%)	131 (19.1%)
	Reg. childcare attend. (3m), n(%)					
		338 (11.3%)	85 (13.5%)	80 (12.5%)	73 (11.3%)	54 (8.4%)
	Unknown	274 (8.4%)	61 (8.8%)	48 (7%)	38 (5.6%)	43 (6.3%)
	Any breastfeeding dur.					
	Median (Range)	10 (0, 30)	8 (0, 25)	10 (0, 26)	11 (0, 24)	11 (0, 30)
	IQR (Q1,Q3)	5, 14	3, 13	5.5, 14	6, 15	6, 15
	Unknown	106 (3.2%)	21 (3%)	12 (1.7%)	15 (2.2%)	12 (1.7%)
	Pet (cat) (1y), n(%)					
		550 (21.4%)	136 (25.7%)	128 (22.2%)	113 (20.2%)	113 (20.4%)
	Unknown	690 (21.1%)	161 (23.3%)	113 (16.4%)	125 (18.3%)	134 (19.5%)
	Pet (dog) (1y), n(%)					
		701 (27.1%)	155 (29.2%)	163 (28.1%)	165 (29.4%)	123 (22.1%)
	Unknown	681 (20.9%)	160 (23.2%)	109 (15.8%)	123 (18%)	131 (19.1%)
	Older siblings (#)					
	Median (Range)	0 (0, 4)	1 (0, 4)	0 (0, 4)	0 (0, 3)	0 (0, 3)
	IQR (Q1,Q3)	0, 1	0, 1	0, 1	0, 1	0, 1
	Unknown	101 (3.1%)	6 (0.9%)	4 (0.6%)	2 (0.3%)	2 (0.3%)
	Postnatal smoking, n(%)					

		620 (23.1%)	241 (41.6%)	148 (25%)	80 (14.1%)	38 (6.7%)
	Unknown	578 (17.7%)	111 (16.1%)	98 (14.2%)	118 (17.3%)	122 (17.8%)
	Exclusive breastfeeding (6m), n(%)					
		522 (17.1%)	94 (14.5%)	94 (14.2%)	120 (18.5%)	151 (23.3%)
	Unknown	208 (6.4%)	41 (5.9%)	30 (4.3%)	36 (5.3%)	39 (5.7%)
	Cleaning product use					
	Median (Range)	31 (4, 76)	32 (4, 76)	31 (8, 76)	31 (7, 66)	31 (9, 68)
	IQR (Q1,Q3)	25, 38	25, 40	24, 37	24, 38	25, 38
	Unknown	341 (10.5%)	79 (11.4%)	64 (9.3%)	56 (8.2%)	55 (8%)
	Multifamily home, n(%)					
		878 (28.9%)	200 (31.6%)	183 (28.1%)	184 (28.3%)	184 (27.9%)
	Unknown	223 (6.8%)	58 (8.4%)	38 (5.5%)	33 (4.8%)	28 (4.1%)
	Clutter home, n(%)					
		581 (19.1%)	186 (29.4%)	131 (20.1%)	86 (13.2%)	74 (11.3%)
Home Environment	Unknown	229 (7%)	59 (8.5%)	38 (5.5%)	34 (5%)	30 (4.4%)
	Potted plant, n(%)					
		1822 (59.9%)	305 (48.2%)	388 (59.5%)	435 (66.8%)	451 (68.4%)
	Unknown	223 (6.8%)	58 (8.4%)	38 (5.5%)	33 (4.8%)	28 (4.1%)
	Carpet floor, n(%)					
		1722 (56.6%)	410 (64.8%)	377 (57.8%)	372 (57.1%)	315 (47.8%)
	Unknown	223 (6.8%)	58 (8.4%)	38 (5.5%)	33 (4.8%)	28 (4.1%)
	Medium/high mould, n(%)					
		359 (11.8%)	87 (13.7%)	73 (11.2%)	75 (11.5%)	68 (10.3%)
	Unknown	223 (6.8%)	58 (8.4%)	38 (5.5%)	33 (4.8%)	28 (4.1%)
	Dwelling density					
	Median (Range)	0.2 (-0.8, 10.7)	0.1 (-0.8, 9.4)	0.1 (-0.8, 10.7)	0.3 (-0.8, 9.7)	0.9 (-0.7, 10)
	IQR (Q1,Q3)	-0.2, 1.4	-0.3, 0.6	-0.2, 0.9	-0.1, 1.6	0, 2.2
	Unknown	197 (6%)	22 (3.2%)	13 (1.9%)	14 (2%)	9 (1.3%)
	Air pollution (NO_2) (preg.)					
	Median (Range)	10.6 (1.3, 35.8)	7.3 (1.3, 30.8)	10.2 (1.3, 26.9)	11.1 (1.4, 33.7)	13 (1.4, 35.8)
	IQR (Q1,Q3)	5.3, 15	4, 12.5	5.1, 14.5	7, 15	9.8, 16.7
	Unknown	191 (5.9%)	40 (5.8%)	34 (4.9%)	40 (5.8%)	33 (4.8%)
	Air pollution (NO_2) (1y)					
Neighborhood Environment	Median (Range)	9.1 (0.5, 30.5)	6.1 (0.5, 26.3)	8.8 (1, 23.1)	9.4 (1.4, 28.7)	11.5 (1.2, 30.5)
	IQR (Q1,Q3)	4.6, 13.3	3.7, 10.7	4.5, 12.3	5.8, 13.3	8.3, 15
	Unknown	194 (5.9%)	41 (5.9%)	36 (5.2%)	39 (5.7%)	35 (5.1%)
	Tree density (preg.)					
	Median (Range)	20 (0, 99)	17 (0, 91)	18 (0, 92)	20 (0, 94)	26 (0, 99)
	IQR (Q1,Q3)	13, 32	11, 23.5	10, 26	14, 32	16, 62
	Unknown	663 (20.3%)	192 (27.8%)	150 (21.7%)	134 (19.6%)	91 (13.2%)
	Rural, n(%)					
		186 (6%)	74 (11.1%)	42 (6.3%)	20 (3.1%)	12 (1.8%)
	Unknown	146 (4.5%)	25 (3.6%)	28 (4.1%)	45 (6.6%)	30 (4.4%)
	Paternal asthma, n(%)					
		510 (19.2%)	98 (18.7%)	106 (18.5%)	116 (19.7%)	119 (19.5%)
	Unknown	600 (18.4%)	168 (24.3%)	116 (16.8%)	96 (14%)	76 (11.1%)
	Paternal atopy, n(%)					
		1663 (67.7%)	292 (62.7%)	353 (65.6%)	394 (71.1%)	402 (72.3%)
	Unknown	806 (24.7%)	225 (32.6%)	152 (22%)	130 (19%)	131 (19.1%)
	Maternal asthma, n(%)					
		749 (23.4%)	176 (25.7%)	153 (22.2%)	163 (23.9%)	150 (21.9%)
	Unknown	56 (1.7%)	6 (0.9%)	2 (0.3%)	1 (0.1%)	1 (0.1%)
	Maternal distress (1y)					
	Median (Range)	12 (0, 40)	14 (0, 40)	12 (0, 32)	11 (0, 36)	10 (0, 32)
	IQR (Q1,Q3)	7, 17	9, 18	8, 17	6, 15	6, 15
Parental Diet/Health	Unknown	685 (21%)	160 (23.2%)	117 (17%)	125 (18.3%)	119 (17.3%)
	Maternal BMI					
	Median (Range)	23.3 (13.9, 56.9)	24.9 (15.6, 56.9)	23.7 (16.4, 54.4)	22.9 (13.9, 43.2)	22.3 (17, 45.8)
	IQR (Q1,Q3)	21, 27	21.5, 30.2	21.5, 27.5	20.8, 25.8	20.5, 24.7
	Unknown	327 (10%)	74 (10.7%)	48 (7%)	57 (8.3%)	69 (10%)
	Maternal UPF diet					
	Median (Range)	46.8 (1.9, 82.9)	50.3 (8.2, 82.9)	46.7 (13.9, 76.5)	45.8 (15.1, 77.1)	44.9 (3.6, 77.3)
	IQR (Q1,Q3)	39.7, 53.8	43.6, 56.9	39.4, 53.7	39.5, 52.1	38.1, 52
	Unknown	291 (8.9%)	49 (7.1%)	32 (4.6%)	47 (6.9%)	42 (6.1%)
	Maternal atopy, n(%)					
		1727 (57.7%)	340 (53.5%)	383 (59%)	377 (59.3%)	400 (63%)
	Unknown	268 (8.2%)	56 (8.1%)	41 (5.9%)	48 (7%)	52 (7.6%)

Table S2. SES factors across all CHILD sites and $COPSAC_{2010}$ cohort, Related to STAR Methods.

CHILD Cohort						COPSAC	
**Variable**	**Cohort**	**Edmonton**	**Toronto**	**Vancouver**	**Winnipeg**	**Variable**	**Copenhagen**
No. patients	3263	768	768	735	992	No. patients	700
Annual household income (CAD), n(%)						Household income (DKK) covering last 3 months, n(%)	
0-49999	399 (14.1%)	82 (12.1%)	38 (5.7%)	73 (10.8%)	206 (25.2%)	0-150.000	237 (33.9%)
50000-99999	943 (33.2%)	239 (35.2%)	130 (19.6%)	219 (32.3%)	355 (43.5%)	150.000-200.000	200 (28.6%)
100000-149999	795 (28%)	214 (31.5%)	183 (27.6%)	209 (30.8%)	189 (23.1%)	200.000-250.000	157 (22.4%)
>=150000	700 (24.7%)	144 (21.2%)	311 (47%)	178 (26.2%)	67 (8.2%)	>250.000	106 (15.1%)
Unknown	426 (13.1%)	89 (11.6%)	106 (13.8%)	56 (7.6%)	175 (17.6%)	Unknown	0 (0%)
Education of mother, n(%)						Education of mother, n(%)	
High school	276 (8.8%)	63 (8.7%)	24 (3.2%)	21 (2.9%)	168 (18.1%)	College or High school	55 (7.9%)
College	905 (28.9%)	281 (38.7%)	143 (19%)	164 (22.7%)	317 (34.1%)	Tradesman	130 (18.6%)
University	1353 (43.2%)	303 (41.7%)	379 (50.3%)	334 (46.2%)	337 (36.3%)	Medium academic	321 (45.9%)
Master or Phd	598 (19.1%)	80 (11%)	207 (27.5%)	204 (28.2%)	107 (11.5%)	University	194 (27.7%)
Unknown	131 (4%)	41 (5.3%)	15 (2%)	12 (1.6%)	63 (6.4%)	Unknown	0 (0%)
Education of father, n(%)						Education of father, n(%)	
High school	475 (15.3%)	126 (17.5%)	52 (7%)	43 (6%)	254 (27.5%)	College or High school	71 (10.4%)
College	1038 (33.5%)	321 (44.6%)	172 (23.1%)	216 (30.2%)	329 (35.6%)	Tradesman	213 (31.3%)
University	1096 (35.3%)	203 (28.2%)	335 (45%)	290 (40.6%)	268 (29%)	Medium academic	206 (30.2%)
Master or Phd	494 (15.9%)	70 (9.7%)	185 (24.9%)	166 (23.2%)	73 (7.9%)	University	191 (28%)
Unknown	160 (4.9%)	48 (6.2%)	24 (3.1%)	20 (2.7%)	68 (6.9%)	Unknown	19 (2.7%)
Social status (canada)						Data not collected	
Median (range)	7 (1, 10)	7 (1, 10)	7 (1, 10)	7 (1, 10)	6 (1, 10)		
IQR (Q1,Q3)	6, 8	6, 8	7, 8	6, 8	5, 7		
Unknown	190 (5.8%)	53 (6.9%)	31 (4%)	15 (2%)	91 (9.2%)		
Social status (community)							
Median (range)	7 (1, 10)	7 (1, 10)	7 (1, 10)	7 (1, 10)	7 (1, 10)		
IQR (Q1,Q3)	6, 8	6, 8	6, 8	6, 8	5, 8		
Unknown	193 (5.9%)	51 (6.6%)	35 (4.6%)	15 (2%)	92 (9.3%)		
SES index						SES index	
Median (range)	0.1 (-2.4, 1.6)	-0.1 (-2.4, 1.3)	0.4 (-1.9, 1.6)	0.2 (-2, 1.6)	-0.2 (-2.3, 1.3)	Median (range)	0 (-1.3, 1)
IQR (Q1,Q3)	-0.4, 0.4	-0.5, 0.3	0, 0.7	-0.2, 0.6	-0.7, 0.2	IQR (Q1,Q3)	-0.4, 0.4
Unknown	511 (15.7%)	108 (14.1%)	132 (17.2%)	65 (8.8%)	206 (20.8%)	Unknown	19 (2.7%)

Table S3. Prevalence of *Bifidobacterium* species in children breastfed up to 3 months across CHILD study sites (presence at either 3-month or 1-year visit) and $COPSAC_{2010}$ cohort (presence at 1-month or 1-year visit) , Related to Figure 6. q-value is calculated based on chi-square test and with adjustment of multiple comparisons using Benjamini-Hochberg (BH) method.

Species	Vancouver	Toronto	Winnipeg	Edmonton	q-value across CHILD study sites	CHILD Study	$COPSAC_{2010}$ Cohort	q-value between CHILD study and $COPSAC_{2010}$ cohort
Bifidobacterium adolescentis	28%	24%	33%	29%	0.12	29%	83%	<0.001
Bifidobacterium animalis	44%	51%	47%	48%	0.47	47%	<10%	<0.001
Bifidobacterium bifidum	48%	40%	50%	45%	0.12	46%	89%	<0.001
Bifidobacterium breve	66%	60%	56%	63%	0.12	61%	94%	<0.001
Bifidobacterium dentium	36%	40%	39%	34%	0.47	38%	38%	0.94
Bifidobacterium infantis	37%	30%	23%	17%	<0.001	28%	75%	<0.001
Bifidobacterium longum	89%	87%	87%	89%	0.94	88%	100%	<0.001
Bifidobacterium pseudocatenulatum	31%	34%	36%	38%	0.47	35%	86%	<0.001

Table S4. Prevalence and mean relative abundance of *B. infantis* across CHILD and COPSAC cohort, Related to Figure 6.

Cohort	Visit	Sample Size (n)	*B. infantis* prevalence (%)	Mean relative abundance (all samples)	Mean relative abundance (*B. infantis*–positive children)
CHILD	3 months	1332	12.8%	0.097	0.753
	1 year	1426	19.1%	0.050	0.261
COPSAC	1 month	598	29.8%	0.0542	0.182
	1 year	645	54.7%	0.0068	0.012

Table S5. Proportion of missing across SES quartiles for NCD risk factors in CHILD study, Related to STAR Methods. P-value was estimated using fisher's exact test.

Health Outcomes at 5 years of age	Cohort	SES Q_1 (0-25%)	SES Q_2 (25-50%)	SES Q_3 (50-75%)	SES Q_4 (75-100%)	P-value
No. patients	3263	691	690	684	687	
Atopic sensitization at 5y, n(%)						0.051
Missing	708 (21.7%)	151 (21.9%)	125 (18.1%)	128 (18.7%)	160 (23.3%)	
Not missing	2555 (78.3%)	540 (78.1%)	565 (81.9%)	556 (81.3%)	527 (76.7%)	
Asthma at 5y, n(%)						0.053
Missing	864 (26.5%)	199 (28.8%)	167 (24.2%)	154 (22.5%)	171 (24.9%)	
Not missing	2399 (73.5%)	492 (71.2%)	523 (75.8%)	530 (77.5%)	516 (75.1%)	
Overweight/obesity at 5y, n(%)						0.047
Missing	638 (19.6%)	142 (20.5%)	109 (15.8%)	113 (16.5%)	137 (19.9%)	
Not missing	2625 (80.4%)	549 (79.5%)	581 (84.2%)	571 (83.5%)	550 (80.1%)	
Emotional/behavioral problem at 5y, n(%)						0.00059
Missing	903 (27.7%)	215 (31.1%)	153 (22.2%)	168 (24.6%)	159 (23.1%)	
Not missing	2360 (72.3%)	476 (68.9%)	537 (77.8%)	516 (75.4%)	528 (76.9%)	
