# Supplementary material for: Breastfeeding may lessen socioeconomic disparities in child health through differences in the infant gut microbiome
Source: Cell Rep Med. 2026 Apr 13;7(4):102755. doi: 10.1016/j.xcrm.2026.102755 (PMC13130633; doi:10.1016/j.xcrm.2026.102755)
Supplement: Document S2. Article plus supplemental information [file mmc2.pdf]

Cell Reports Medicine

Article

Breastfeeding may lessen socioeconomic disparities in child health through differences in the infant gut microbiome

Graphical abstract

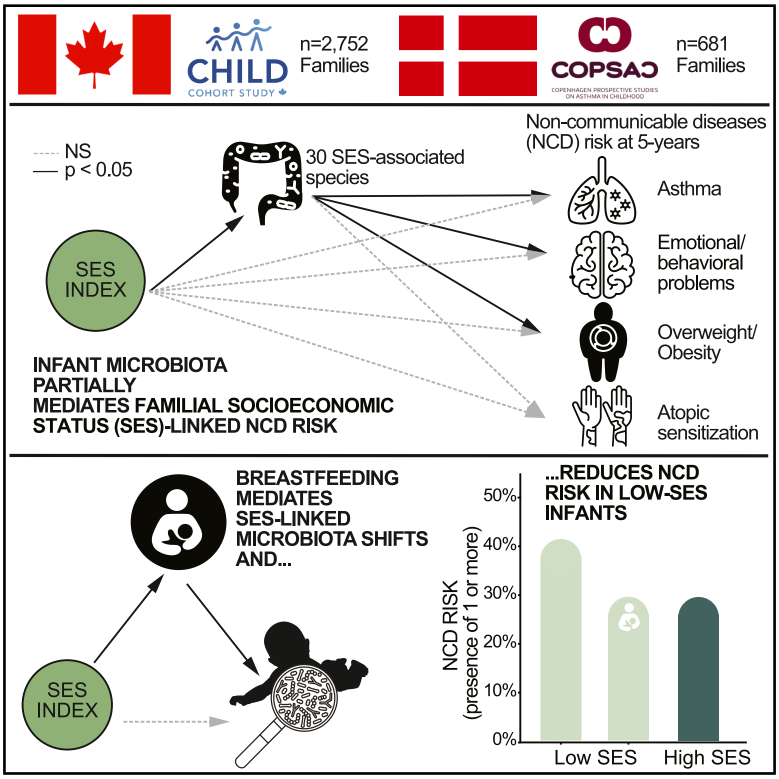

Highlights

- Family SES shapes perinatal exposures and predicts later child health outcomes
- Breastfeeding shields infant microbiota and reduces risks for disadvantaged infants
- Infant gut microbiota partly mediates links between SES and child health
- Canadian findings were replicated in an independent Danish birth cohort

Authors

Darlene L.Y. Dai, Melissa B. Manus, Courtney Hoskinson, ..., Jakob Stokholm, Charisse Petersen, Stuart E. Turvey

Correspondence

charisse.petersen@bcchr.ca (C.P.),
sturvey@cw.bc.ca (S.E.T.)

In brief

Darlene Dai, Charisse Petersen, Stuart Turvey, and colleagues report that breastfeeding may mitigate the adverse effects of socioeconomic disadvantage on early risk factors for non-communicable diseases. The authors link this effect to differences in infant gut microbiota, including enrichment of *Bifidobacterium infantis*, highlighting potentially modifiable pathways underlying intergenerational health inequities.

Dai et al., 2026, Cell Reports Medicine 7, 102755
April 21, 2026 © 2026 The Author(s). Published by Elsevier Inc.
https://doi.org/10.1016/j.xcrm.2026.102755

CellPress

Cell Reports Medicine

CellPress
OPEN ACCESS

Article

Breastfeeding may lessen socioeconomic disparities in child health through differences in the infant gut microbiome

Darlene L.Y. Dai,[1] Melissa B. Manus,[2] Courtney Hoskinson,[1,3] Jie Jiang,[4,5] Hind Sbihi,[6,7] Kozeta Miliku,[8,9] Susan C. Campisi,[10,11] Daphne J. Korczak,[11,12] Qingling Duan,[13,14] Theo J. Moraes,[15] Piushkumar J. Mandhane,[16,17] B. Brett Finlay,[3,18,19] Elinor Simons,[20] Hannah Lishman,[6] David M. Patrick,[6,7] Padmaja Subbarao,[9,15,21] Meghan B. Azad,[20,22,23] Bo Chawes,[4,24] Klaus Bønnelykke,[4,24] Søren Johannes Sørensen,[25] Jonathan Thorsen,[4,24] Jakob Stokholm,[4,5,26] Charisse Petersen,[1,*] and Stuart E. Turvey[1,27,*]

[1]Department of Pediatrics, BC Children's Hospital, University of British Columbia, Vancouver, BC, Canada
[2]Department of Anthropology, University of Texas at San Antonio, San Antonio, TX, USA
[3]Department of Microbiology and Immunology, University of British Columbia, Vancouver, BC, Canada
[4]COPSAC, Copenhagen Prospective Studies on Asthma in Childhood, Copenhagen University Hospital - Herlev and Gentofte, Copenhagen, Denmark
[5]Department of Food Science, Faculty of Science, University of Copenhagen, Frederiksberg C, Denmark
[6]BC Centre for Disease Control, Vancouver, BC, Canada
[7]School of Population and Public Health, University of British Columbia, Vancouver, BC, Canada
[8]Department of Nutritional Sciences, Temerty Faculty of Medicine, University of Toronto, Toronto, ON, Canada
[9]Department of Medicine, Faculty of Health Sciences, McMaster University, Hamilton, ON, Canada
[10]Nutrition and Dietetics Program, Clinical Public Health Division, Dalla Lana School of Public Health, University of Toronto, Toronto, ON, Canada
[11]Department of Psychiatry, The Hospital for Sick Children, Toronto, ON, Canada
[12]Department of Psychiatry, University of Toronto, Toronto, ON, Canada
[13]Department of Biomedical and Molecular Sciences, Queen's University, Kingston, ON, Canada
[14]School of Computing, Queen's University, Kingston, ON, Canada
[15]Department of Pediatrics, The Hospital for Sick Children, Toronto, ON, Canada
[16]Department of Pediatrics, University of Alberta, Edmonton, AB, Canada
[17]Faculty of Medicine and Health Sciences, UCSI University, Kuala Lumpur, Malaysia
[18]Michael Smith Laboratories, UBC, Vancouver, BC, Canada
[19]Department of Biochemistry and Molecular Biology, University of British Columbia, Vancouver, BC, Canada
[20]Section of Allergy and Immunology, Department of Pediatrics and Child Health, University of Manitoba, Winnipeg, MB, Canada
[21]Dalla Lana School of Public Health, University of Toronto, Toronto, ON, Canada
[22]Department of Food and Human Nutritional Sciences, University of Manitoba, Winnipeg, MB, Canada
[23]Manitoba Interdisciplinary Lactation Centre (MILC), Children's Hospital Research Institute of Manitoba, Winnipeg, MB, Canada
[24]Department of Clinical Medicine, Faculty of Health and Medical Sciences, University of Copenhagen, Copenhagen, Denmark
[25]Section of Microbiology, University of Copenhagen, Copenhagen, Denmark
[26]Department of Pediatrics, Slagelse Hospital, Slagelse, Denmark
[27]Lead contact
*Correspondence: charisse.petersen@bcchr.ca (C.P.), sturvey@cw.bc.ca (S.E.T.)
https://doi.org/10.1016/j.xcrm.2026.102755

SUMMARY

Lower familial socioeconomic status (SES) is linked to increased childhood disease risk. Since SES has no inherent biological basis, identifying how it becomes physiologically embedded is essential for equitable intervention. Using data from the Canadian CHILD birth cohort (n = 2,752) with replication in the Danish Copenhagen Prospective Studies on Asthma in Childhood 2010 ($COPSAC_{2010}$) cohort (n = 681), we analyze modifiable pathways linking SES to child health and find that the infant gut microbiota plays a key mediating role. Breastfeeding is associated with a stabilized infant microbiota, buffering against environmental impacts and reducing health risks in lower SES contexts. The presence of *Bifidobacterium infantis*, enriched through breastfeeding, is linked to protection against adverse outcomes from SES. Together, these results suggest that improving breastfeeding rates and restoring breastfeeding-enriched microbes, like *B. infantis*, may help buffer early biological impacts of social inequality and support healthier trajectories for children growing up in industrialized settings.

© 2026 The Author(s). Published by Elsevier Inc.
This is an open access article under the CC BY license (http://creativecommons.org/licenses/by/4.0/).

INTRODUCTION

Socioeconomic disparity and health inequity are closely intertwined, with consequences that can span generations. Lower socioeconomic status (SES), a social construct with no inherent biological basis, is associated with heightened risk of chronic non-communicable diseases (NCDs), including cardiometabolic disease, chronic respiratory disease, and mental illness.[1–4] These health disparities can emerge early in life, as limited socioeconomic resources increase children's likelihood of developing NCD-associated conditions.[3,5] Moreover, industrialization has coincided with an increased prevalence of NCDs, which globally now account for approximately 70% of all deaths and are increasingly affecting children as well,[6] exacerbating both the issue and its disproportionate impact on vulnerable populations.[7,8] Understanding how socioeconomic inequity becomes biologically embedded is, therefore, essential, yet the mechanisms driving this process remain unclear. Identifying modifiable pathways through which SES affects health could inform innovative strategies that equitably support childhood health and ultimately reduce the burden of NCDs.[9,10]

The rise of NCDs closely follows factors linked to industrialization, such as antibiotic use, urbanization, processed food diets, and a highly sanitized environment, all of which have reshaped human interactions with the microbial world.[11–13] As a result, industrialization has driven intergenerational changes in both our individual microbiome, the communities of microbes living in and on our bodies, as well as the broader metacommunity of microbes capable of colonizing all of us.[14,15] Researchers have justifiably identified the microbiota as a potential pathway by which essential microbial exposures that support healthy development are diminished or altered.[8,16,17] Many SES-related factors also affect the microbiota, especially during early life, including breastfeeding initiation and duration, antibiotic exposure, and access to green spaces and fresh foods.[10,18,19] Since the infant microbiota is both highly sensitive to disruptions and critical for healthy development, it may serve as a nexus linking SES-related exposures to childhood health outcomes. Thus, safeguarding or restoring the early-life microbiota represents an innovative opportunity to limit the rise of NCDs.[18] However, measures to ensure early exposure to essential microbes, while limiting factors that disrupt health-promoting microbial development, must be implemented equitably to help break cycles of health disparities.[9]

The infant microbiota matures alongside multiple rapidly developing physiological systems, such as the immune, metabolic, and neurobehavioral systems.[20,21] Early-life microbiota disruptions have been associated with multiple diseases linked to metabolic dysfunction, asthma, and allergies.[9,22,23] While a growing body of evidence suggests that familial SES influences the infant and childhood gut microbiota,[24–26] comprehensive analyses of the environmental factors mediating this relationship are limited. Moreover, SES is a multidimensional construct that typically encompasses not only family income but also educational attainment and subjective perceptions of social status, yet studies typically investigate these different social factors in isolation.[24–26] Integrating SES parameters with early-life exposures to define their impact on the infant microbiota and health could identify critical SES-related drivers of microbiota development, highlighting opportunities to optimize the early-life microbiota and improve population health.

In this study, we leveraged the CHILD Cohort Study, the largest prospective birth cohort in Canada, which includes diverse families across socioeconomic, geographic, and ethnic backgrounds. The study captured perinatal measures of SES, parental demographics and history, environmental exposures, infant gut microbiota profiles, and physician-defined health outcomes at age 5.[27] We investigated how familial SES shaped early-life exposures and parental well-being and assessed its associations with infant microbiota composition and later child health. Regression models revealed considerable overlap between the associations of familial SES and breastfeeding rates on both infant microbiota composition and multiple health outcomes, with consistent directional patterns. Furthermore, microbes enriched by breastfeeding, most notably *Bifidobacterium infantis*, were linked to protection against several childhood health risk factors for NCDs. We replicated key associations in the independent Copenhagen Prospective Studies on Asthma in Childhood 2010 ($COPSAC_{2010}$) cohort from Denmark, reinforcing links among SES, breastfeeding, the infant microbiome, and child health. Together, these findings suggest that supplementation with key beneficial bacteria, alongside efforts to reduce breastfeeding barriers, may offer scalable strategies to improve child health and reduce socioeconomic health disparities.

RESULTS

Familial SES is associated with multiple domains of perinatal experience and links to future adverse child health outcomes

The Canadian CHILD Study collected five measures of SES when enrolling pregnant women and their partners in the study at approximately 18 weeks of gestation. These included the household income, the highest education of mother and father, and the MacArthur scale of subjective social status in both Canada and their local community.[27,28] Of the 3,263 eligible families enrolled in the general cohort of the CHILD Study (Figure S1A; Table S1), 2,752 children had complete data of the five individual SES factors and were used to construct a single "SES index" integrating all five measures based on confirmatory factor analysis (CFA) (Figure 1A). Independent cluster analysis confirmed the index's internal consistency ($\alpha = 0.76$) (Figure S1B). A single SES index was derived from this estimated latent factor and was used in all downstream analyses.

We next sought to understand how SES related to 36 perinatal factors collected between 18 weeks of gestation and infants' 1-year visit. These factors spanned parental diet and health, prenatal exposures, specific measures related to birth, postnatal exposures within the first year, and the home or local community environment (Figure 1B). Of the 36 perinatal variables analyzed, nearly half were associated with SES after correction for multiple testing (false discovery rate [FDR]<0.05). Moreover, SES was significantly linked to factors across multiple exposure types, highlighting the broad and integral role of SES in shaping parental health and exposures during the perinatal period. Sensitivity analyses confirmed that our SES index is a good

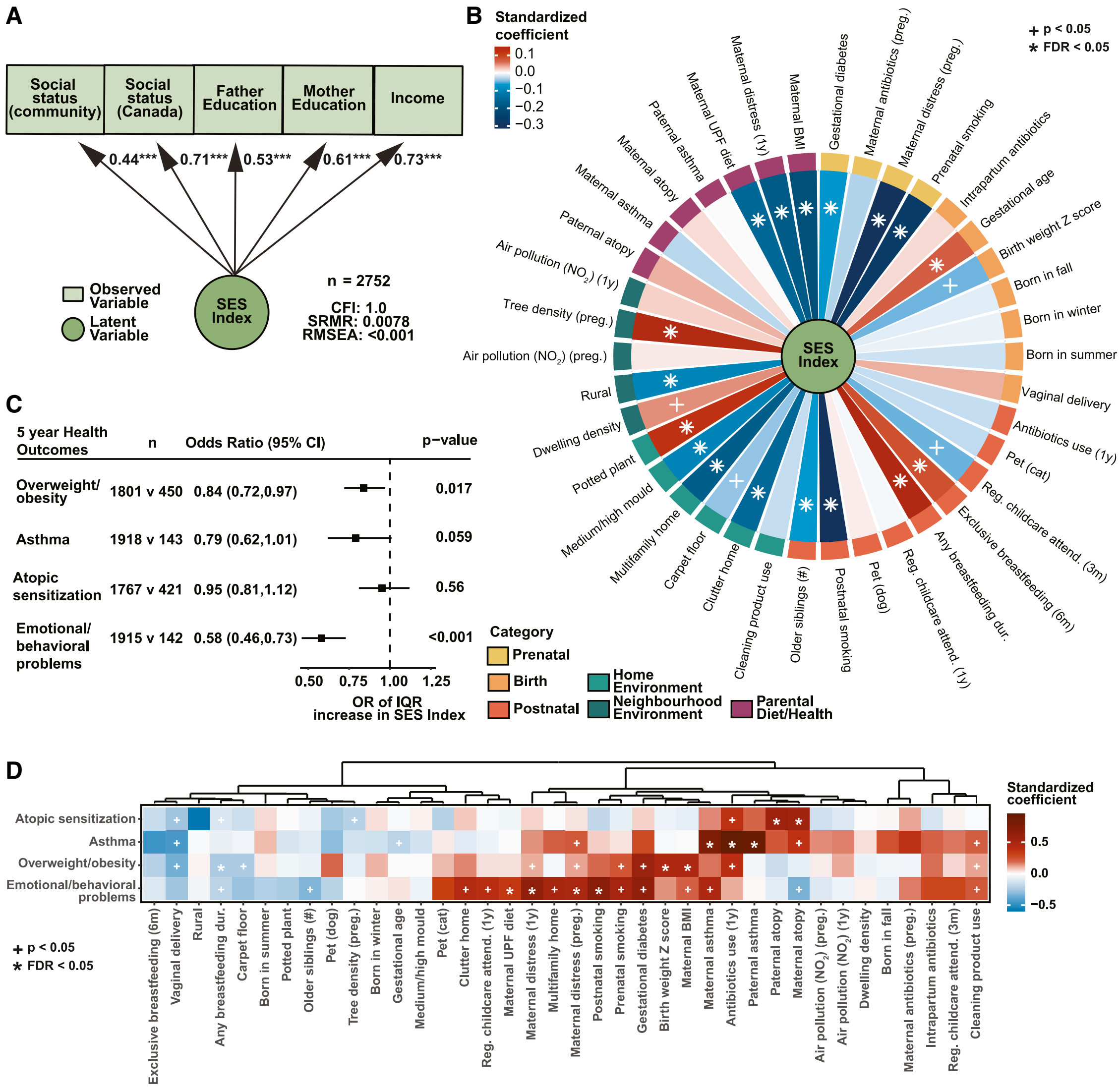

Figure 1. Summarized SES index is associated with childhood NCD risk factors and perinatal factors and exposomes

(A) SES index derived from observed SES factors using confirmatory factor analysis. Circles denote latent variables, and rectangles represent observed variables. One-headed arrows indicate standardized path coefficients between variables (***$p < 0.001$). CFI, comparative fit index; RMSEA, root-mean-square error of approximation; SRMR, standardized root mean residuals.

(B) Associations between SES and perinatal factors. Values show standardized coefficients of perinatal factors on SES index collected during pregnancy.

(C) Forest plot of odds ratio (95% CI) for the association between an interquartile range (IQR) increase in SES and childhood NCD risk factor at age 5.

(D) Heatmap of associations between childhood health outcomes at age 5 and perinatal and exposome factors. Associations reflect standardized coefficients from logistic regression adjusted for study site and sex. Factors are ordered by hierarchical clustering. Red represents positive association, and blue represents negative associations, with + indicating $p < 0.05$ and * indicating FDR<0.05.

representor of the 5 observed SES factors and robustly associates with perinatal factors (Figure S1C).

SES has furthermore been associated with the likelihood of developing NCDs.[3,5] We, therefore, assessed adverse health outcomes at age 5 years that are early risk factors for multiple NCDs, including atopic (also known as allergic) sensitization (defined by a positive allergen skin prick test), physician diagnosis of asthma, overweight or obesity classifications (determined by BMI *Z* scores), and emotional/behavioral problems (defined using the childhood behavioral checklist). Infants from families with higher SES had significantly lower odds of overweight/obesity (odds ratio [OR] for an interquartile range [IQR] increase in SES index = 0.84, $p = 0.017$) and emotional or behavioral problems (OR = 0.58, $p < 0.001$), with a trend toward protection from asthma (OR = 0.79, $p = 0.059$). In contrast, SES was not associated with 5-year atopic sensitization (Figure 1C). Reinforcing the importance of the early-life environment in the developmental origins of health and disease, 12 of the 36 perinatal factors were significantly (FDR<0.05) associated with at least one 5-year health outcome (Figure 1D). Notable

protective factors included longer breastfeeding duration. In contrast, factors such as early antibiotic exposure (within the first year) and maternal distress during pregnancy were positively associated with adverse health outcomes (FDR<0.05). Together these data demonstrate that SES is linked to risk factors for multiple distinct NCDs within the CHILD cohort, supporting further investigating the biological underpinnings of SES.

Familial SES significantly associates with variation in the infant gut microbiota

The infant gut microbiota is highly sensitive to disruption, including by factors associated with SES, and changes in the microbiota during early life can have long-lasting effects on physiological and behavioral development.[9,10,18,22,23] After defining the associations between SES and perinatal exposures (Figure 1), we next examined how SES and these perinatal factors were associated with the infant microbiota. We analyzed shotgun metagenomic profiles of the gut microbiota from 1,479 participants with stool samples collected at clinical assessment scheduled for 3 months and/or 1 year of age (Figure S1). Infants from higher-SES families had significantly lower alpha diversity at 3 months (continuous SES index: $\beta_{SES} = -0.09$, $p = 0.0011$; lowest vs. highest quartile by Wilcoxon $p < 0.001$) and experienced a greater increase in diversity by age 1 year ($\beta_{SES} = 0.12$, $p = 0.0019$; Wilcoxon $p = 0.0035$) (Figures 2A and S2A). Although higher microbial biodiversity is typically associated with positive health outcomes, there exists a well-known paradox that breastfeeding, considered beneficial early in life, constrains or delays diversification in early infancy.[22,29] Consistent with this, breastfeeding rates at 3 months and 1 year were higher in the highest SES quartile (Q4) (89% and 45%, respectively) than in the lowest SES quartile (Q1) (70% and 31%). Notably, when adjusting for current breastfeeding, the differences in alpha diversity between lower and higher SES groups became non-significant (Figure S2A), suggesting that the observed associations were driven by higher breastfeeding rates in families with higher SES. We then evaluated the association between SES and the taxonomic microbiota composition (beta diversity) at 3 months and 1 year using Aitchison's distances (Figure 2B). Principal coordinate analysis (PCoA) revealed significant differences between the lowest and highest SES quartiles at 3 months along the first two PCoA axes ($p < 0.001$), with a clear dose-response association for SES (continuous SES index: PCoA1 $\beta_{SES} = 1.39$, PCoA2 $\beta_{SES} = -1.53$, $p < 0.001$). Within 1-year samples, SES associations were more modest but still evident in the third PCoA axis, which was significantly associated with the SES index (PCoA3 $\beta_{SES} = -0.63$, $p = 0.036$) (Figures 2B and S2B). These results indicate that the association between SES and the infant microbiota is strongest at 3 months but still detectable at 1 year.

As SES itself is a social construct that is not directly biologically linked to the microbiota, we explored which perinatal exposures could be mediating this effect. For each time point, we applied permutation-based multivariate analysis of variance (PERMANOVA) with the study center as strata and adjusting for technical covariates, to compare the influence of SES and each perinatal factor (Figure 2C). Together, the SES index and perinatal exposures accounted for 9.98% and 10% of the microbiota community variation at the 3-month and 1-year time points, respectively, which although incomplete, is comparable to variation explained in other similar population studies.[30] SES ranked as the fourth most influential factor at 3 months ($R^2 = 0.68\%$, $p < 0.001$) and sixth at 1 year ($R^2 = 0.31\%$, $p < 0.001$). Only extensively validated influencers of the infant microbiota, such as breastfeeding status, delivery mode, and the number of older siblings, had a stronger association than SES at 3 months (Figure 2C). A biplot visualizing the top 10 influencing factors at both time points indicated that SES and breastfeeding consistently moved in similar directions at both time points (Figure 2D). To further investigate the mediating role of breastfeeding, we employed structural equation modeling (SEM), a statistical approach that tests how well-observed data fit a hypothesized causal framework. Using PCoA1 at 3 months and PCoA3 at 1 year to represent the overall SES-associated gut microbiota, we found that among the 36 perinatal factors, current breastfeeding at the time of stool sample collection was the only significant mediator at both 3 months (indirect effect $\beta_{std} = 0.048$, $p < 0.001$) and 1 year ($\beta_{std} = -0.044$, $p = 0.038$) (Figure 2E). These findings indicate that differences in breastfeeding mediated the relationship between SES and the microbiota at both time points, suggesting that breastfeeding may be a key pathway through which SES-related health inequities arise within the infant microbiota to impact long-term health trajectories.

Breastfeeding shields the infant microbiota and can mitigate health risks in infants born to lower-SES families

Human milk plays a key role in shaping the infant gut microbiota and has been demonstrated to pace microbial development and select for keystone species critical for infant health.[29,31,32] To assess the broader extent of this buffering effect, we first compared the overall beta-dispersion of 3-month and 1-year samples based on participant breastfeeding status (Figure 3A). Participants who were not breastfeeding (No BF) by the 3-month visit were compared to infants who were still breastfeeding (BF) at the time of sample collection. At 3 months, the BF infants had significantly lower beta-dispersion compared to the No BF infants (p value<0.001) (Figure 3A). These differences in beta-dispersion were no longer detectable in the 1-year stool sample (p value = 0.6). To control for the unbalanced group sizes between BF and No BF infants, we applied a bootstrapping approach where we randomly selected the same number of samples as the smaller group with replacement for 100 iterations and performed PERMANOVA in each run. Factors that were significant ($p < 0.05$) in at least 80% of runs were considered significant. Using this approach, we found that at 3 months, 20 perinatal factors were significantly associated with microbiota composition in No BF infants, whereas only three factors remained significant in BF infants (Figure 3B). Similarly, at 1 year, although breastfeeding no longer significantly constrained microbiota beta-dispersion, only 1 of the 18 perinatal factors that explained significant variance in No BF infants remained significant in BF infants. These data suggest that the presence of human milk is associated with less microbiota variability in young infants, as measured by beta-dispersion, and with reduced influence of external factors during the first year of life, consistent with a potential stabilizing effect of breastfeeding.

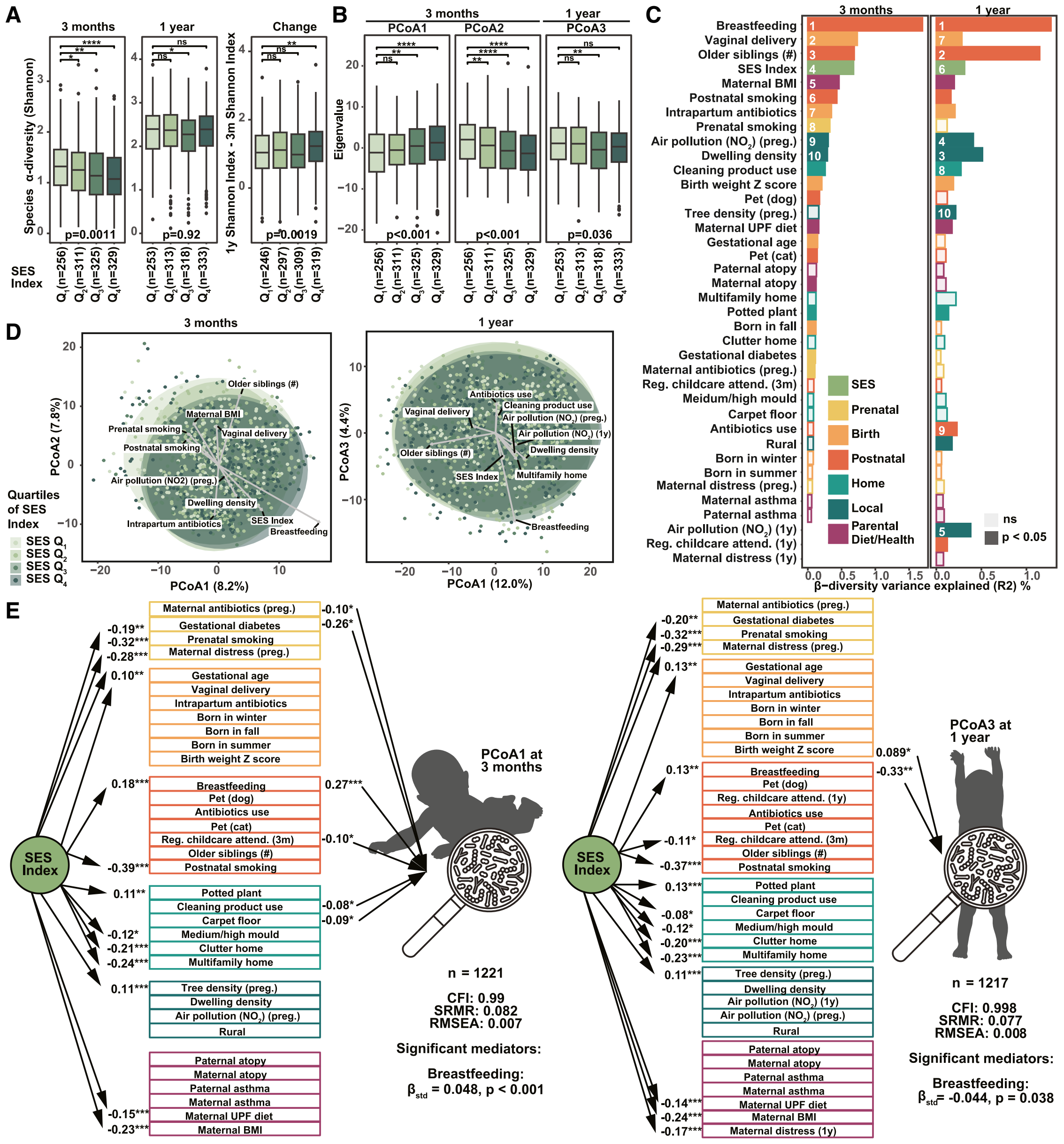

Figure 2. SES a top influencer on infant gut microbiota, primarily through breastfeeding

(A and B) Gut microbiota α-diversity (Shannon index) at 3 months and 1 year and the change between these ages (A); the top three significant PCoA axes (B) across SES quartiles (Q_1 = lowest 25%, Q_4 = highest 25%). Wilcoxon tests compare each quartile to Q_1. *p* values shown beneath each panel are from regression models using the continuous SES index with study site as a random effect and adjustment for stool collection age and processing time. ns, $p > 0.05$; *$p < 0.05$, **$p < 0.01$, ***$p < 0.001$.

(C) Variance explained (R^2) for infant gut microbiota beta-diversity at 3 months and 1 year, with the top 10 significant factors labeled. Bars are colored only for factors with $p < 0.05$.

(D) PCoA plots of 3-month and 1-year beta-diversity, colored by SES quartiles. Arrows indicate Pearson correlations between metadata variables and microbiome PCoAs. Ellipses show 95% confidence regions using a multivariate t-distribution.

(E) SEM models linking SES, perinatal factors and exposomes, and infant gut microbiota (PCoA1 at 3 months on left; PCoA3 at 1 year on right). Only significant indirect effects and associations (one-headed arrows; $p < 0.05$) are shown.

To define the association between breastfeeding and child health outcomes, we divided participants into above and below median of the SES index and quantified their likelihood of developing one or more of the SES-associated NCD risk factors (childhood overweight/obesity, asthma, or emotional/behavioral problems at 5 years) based on whether they were exclusively breastfed for 6 months, which is the duration currently recommended by the WHO. Among participants from low-SES

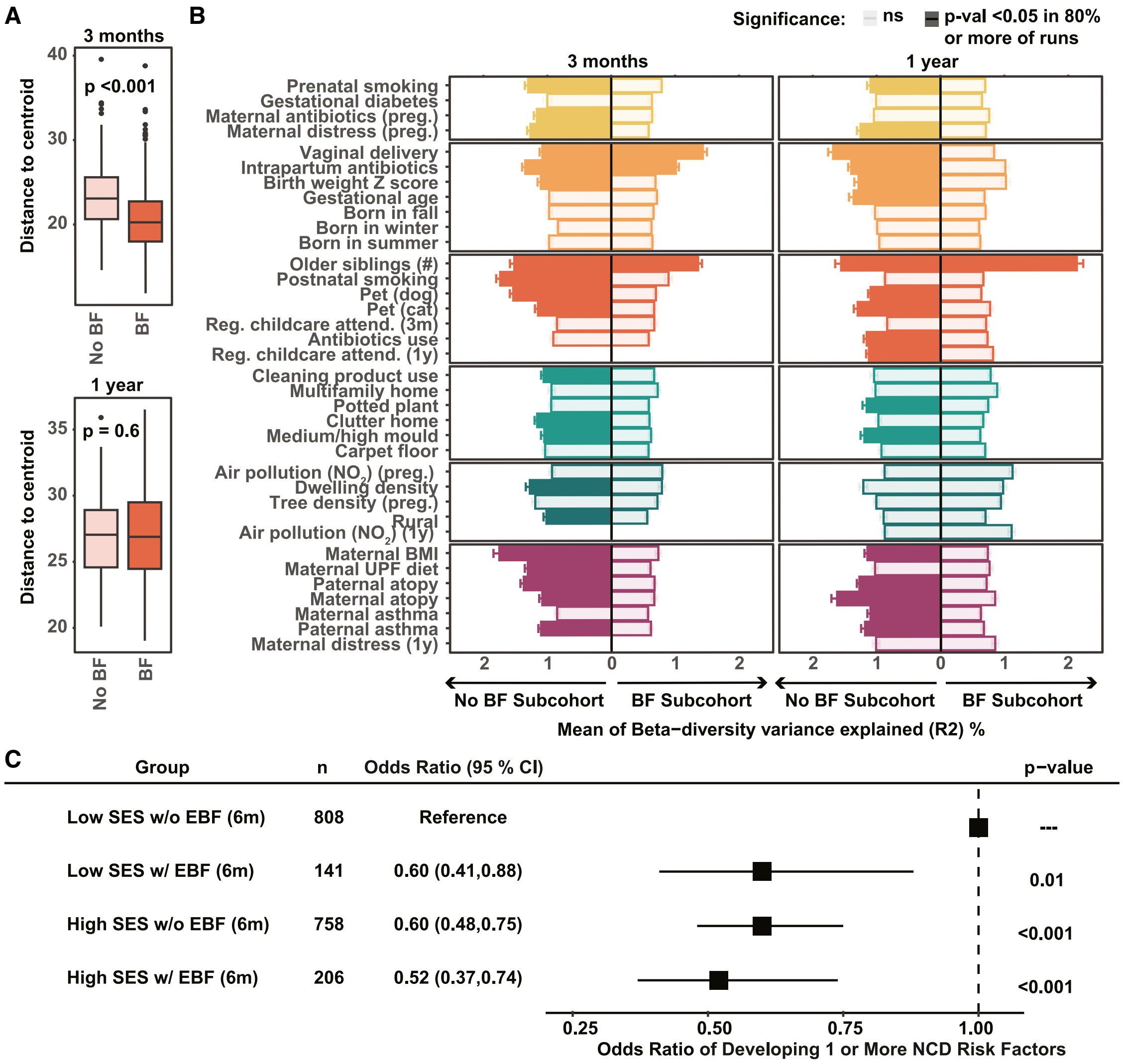

Figure 3. Breastfeeding shields the infant microbiota and can mitigate health risks in infants with limited SES

(A) Beta-dispersion at 3 months and 1 year among children who were breastfeeding (BF) at the stool sample collection age (3 months $n = 1{,}001$, 1 year $n = 479$) and those who had never breastfed or stopped by the 3-month visit (No BF; 3 months $n = 159$, 1 year $n = 149$). Beta-dispersion was calculated based on Aitchison's distance on modified centered log-ratio-transformed relative abundance. Group differences were tested using Wilcoxon tests.

(B) Variance explained (R^2) by each factor for infant gut microbiota at 3 months and 1 year in BF and No BF subgroups, estimated via 100 bootstrap runs with matched sample sizes. Error bars show ±standard error of the mean across runs. Variables with $p < 0.05$ in at least 80% of runs were considered significant and are colored by factor category.

(C) Forest plot of odds ratio (95% CI) for compound SES and EBF at 6 months on the presence of one or more SES-associated NCD risk factor at age 5 (childhood overweight/obesity, asthma, and emotional/behavioral problems).

families, exclusive breastfeeding for 6 months was associated with 40% reduced odds of developing one or more NCD risk factors (OR = 0.60; 95% confidence interval [CI]: 0.41–0.88; $p = 0.01$) (Figure 3C). Notably, this protective association was comparable to that observed in participants from high-SES families, who were protected compared to low-SES families regardless of breastfeeding status (OR = 0.60, 95% CI: 0.48–0.75, and OR = 0.52, 95% CI: 0.37–0.74, for high-SES without and with BF, respectively, both $p < 0.001$). In addition, sensitivity analysis examining any breastfeeding up to 3 months was performed, and we found a similar pattern to the exclusive breastfeeding status (EBF)-based analysis (Figure S3A). These findings indicate that breastfeeding not only mediates the effect of SES on the infant microbiota but also may dampen the impact of external factors and associate with reduced adverse health outcomes among children from lower-SES families.

Replication in the independent Danish COPSAC$_{2010}$ cohort

To replicate key findings in an independent cohort, we turned to the COPSAC$_{2010}$, a population-based Danish birth cohort comprising 700 mother-child pairs. SES was assessed at

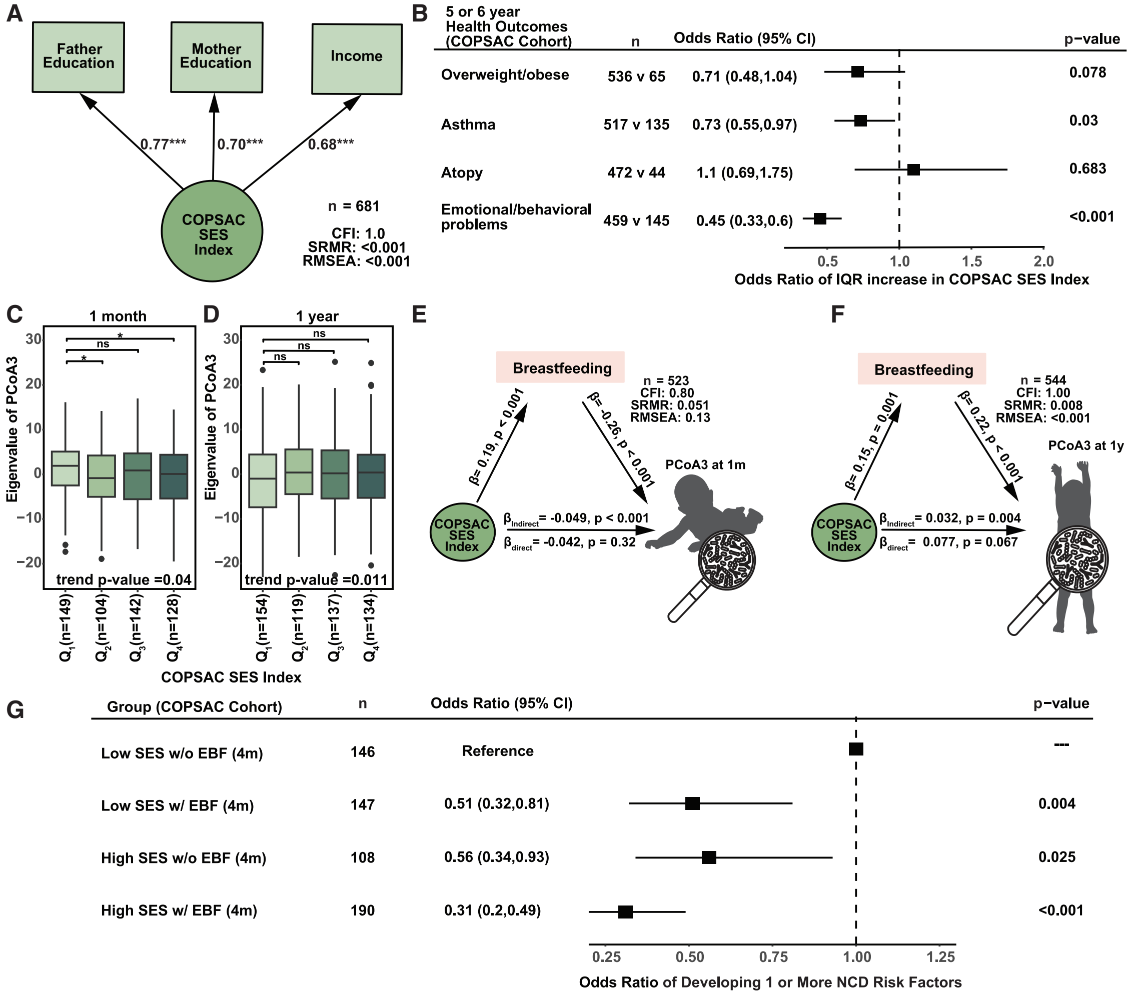

Figure 4. Replication in the independent Danish COPSAC$_{2010}$ cohort

(A) The summarized COPSAC$_{2010}$ SES index derived from observed SES variables using confirmatory factor analysis. Circles denote latent variables, and rectangles represent observed variables. One-headed arrows indicate standardized path coefficients between variables (***p < 0.001). CFI, comparative fit index; RMSEA, root-mean-square error of approximation; SRMR, standardized root mean residuals.

(B) Forest plot of odds ratio (95% CI) for the association between an interquartile increase in SES and childhood NCD risk factors at 5 or 6 years.

(C and D) Boxplot of the PCoA3 across SES quartiles at 1 month (C) and 1 year (D). Wilcoxon tests compare each quartile to Q_1. p values shown beneath each panel are from regression models using the continuous SES index with adjustment for stool collection age and shipment time. ns, $p > 0.05$; *$p < 0.05$.

(E and F) SEM models linking the relationship between SES index, breastfeeding, and PCoA3 of infant gut microbiota at 1 month (E) and 1 year (F).

(G) Forest plot of odds ratio (95% CI) of compound COPSAC$_{2010}$ SES and exclusive breastfeeding status at 4 months on the presence of one or more SES-associated NCD risk factors at age 5 or 6 (overweight or obesity, asthma, and emotional or behavioral problems).

enrollment of the child 1 week after birth, using three indicators: household income and maternal and paternal education. Subjective social status was not collected in COPSAC$_{2010}$ (Table S2). Among 681 children with complete SES data, a latent SES index was constructed (Figure 4A). To ensure comparability with CHILD, we performed a sensitivity analysis by reconstructing SES index using SES factors without subjective social status in CHILD cohort and confirmed it is highly correlated with the original SES index (Pearson R = 0.83, p value <2.2e−16) and provides consistent results (Figures S3B–S3F). Consistent with results from the CHILD cohort, higher SES in COPSAC$_{2010}$ was associated with reduced risk of physician-diagnosed asthma at age 5 years (OR per IQR increase in SES index = 0.73, p = 0.03), emotional or behavioral problems (OR = 0.45, p < 0.001) at age 6 years, and a trend toward protection from overweight/obesity at age 5 years (OR = 0.71, p = 0.078). No association was observed between SES and atopic sensitization at age 6 years (Figure 4B).

We next tested whether breastfeeding mediated the relationship between SES and the infant gut microbiome in the COPSAC$_{2010}$ study using SEM. PCoA was applied to gut microbiota profiles at 1 month (n = 523) and 1 year (n = 544). In contrast to the CHILD cohort, SES-related variation in COPSAC$_{2010}$ microbiota profiles was limited to PCoA3 at both time points

(regression model using continuous SES index: 1-month $p = 0.04$; 1-year $p = 0.011$) (Figures 4C and 4D), possibly reflecting reduced SES variability within the cohort. These axes were used in the SEM to capture SES-associated microbiota variation (Figures 4E and 4F). At 1 year, breastfeeding showed a significant indirect effect ($\beta = 0.032$, $p = 0.004$). Although the SEM model at 1 month exhibited limited fit, possibly due to reduced variation or sample size constraints, the indirect effect of breastfeeding remained statistically significant ($\beta = -0.049$, $p < 0.001$). Together with the CHILD results, these findings from $COPSAC_{2010}$ support a consistent role for breastfeeding in shaping SES-related microbiota trajectories.

We also assessed whether breastfeeding modified SES-related health outcomes in the $COPSAC_{2010}$ cohort. In the $COPSAC_{2010}$ cohort only a small proportion of mothers (5%) exclusively breastfed up to 6 months. To better reflect real-world practices in Denmark and maintain sufficient statistical power, we used exclusive breastfeeding until 4 months instead of 6 months in COPSAC. Among children from lower-SES families, exclusive breastfeeding for 4 months was associated with a 49% reduction in the odds of adverse outcomes (OR = 0.51, 95% CI: 0.32–0.81, $p = 0.004$) (Figure 4G). These findings mirror those from the CHILD cohort and highlight breastfeeding as a modifiable factor that may help mitigate the effects of socioeconomic disadvantage on early microbiota development and long-term health risk.

Most SES-associated species are associated with breastfeeding in consistent directions

We next identified specific microbial species that may be impacted by SES in the first year of life. Using linear mixed-effect models (MaAsLin2), we identified 30 species significantly associated with SES, either through differences in overall abundance or changes over time (FDR<0.1). Of these, 5 species showed overall higher colonization with increasing SES: *B. infantis*, *Phocaeicola dorei*, *Bacteroides fragilis*, *Bacteroides thetaiotaomicron,* and *Bacteroides caccae*. Conversely, 3 species showed consistently lower colonization with higher SES: *Ruthenibacterium lactatiformans*, *Clostridium innocuum*, and *Phocaeicola vulgatus* (Figures 5A and S4A). These 8 species exhibited relatively stable SES-associated differences across infancy. In contrast, 22 species showed significant differences in temporal changes in colonization based on familial SES (Figures 5A and S4A), highlighting SES's extended association with microbial composition within the first year of life. Notably, all 30 species were associated with at least one of the 36 perinatal variables, with breastfeeding status linked to 23 (77%) of the species in a consistent direction (Figures 5A and S4B). For microbes significantly associated with SES or breastfeeding, both the overall effect (overall colonization levels, 53 species) and the slope effect (colonization changes over time, 48 species) showed a strong positive correlation between SES and breastfeeding coefficients (overall effect: Pearson R = 0.45, $p = 0.0007$; slope effect: R = 0.71, $p = 1.6e-08$) (Figure 5B). Moreover, functional characterization of the shotgun sequencing profiles using MetaCyc pathway classifications revealed a similar relationship between SES and breastfeeding (Figure S4C). These data reinforce the interconnected influence of SES and breastfeeding on shaping both the taxonomic composition and functional potential of the infant microbiota.

To assess the reproducibility of these findings, we applied the same approach to the $COPSAC_{2010}$ cohort. Among the 151 microbial species detected in at least 10% of $COPSAC_{2010}$ participants, 15 were significantly associated with SES (FDR <0.1) (Figure S5A). Of these 15 species, 5 were also associated with breastfeeding, including *B. infantis*, *Escherichia coli*, *Veillonella atypica*, *Streptococcus salivarius* (SGB8005), and *Streptococcus parasanguinis*. Notably, four of these species overlapped with the CHILD cohort findings: *B. infantis*, *V. atypica, B. dentium*, and *S. salivarius* (SGB8005). Given the geographic and national differences between the $COPSAC_{2010}$ and CHILD cohorts, variation in microbiota composition was expected; however, the overlap of four key species and 5 of the 15 SES-associated species also associated with breastfeeding in consistent directions reinforces the consistency of these associations across cohorts (Figure S5B).

Health benefits afforded by familial SES are mediated through changes in the infant microbiota

Our findings so far indicate that familial SES is associated with both the infant gut microbiota and multiple distinct health outcomes at 5 years in two independent cohorts. To explore whether modifying the microbiota could improve childhood health, we determined whether the microbiota mediated associations between SES and health outcomes in the CHILD Study. We again used SEM, in which the microbiota was represented by a latent factor comprising all 30 SES-associated species abundances at 3 months and 1 year using CFA. Consistent with the lack of association between SES and 5-year atopic sensitization, the indirect effect of the SES-associated microbiota on atopic sensitization was not significant ($\beta_{std} = 0.003$, $p = 0.19$). In contrast, the SES-associated microbiota emerged as a significant mediator for childhood overweight/obesity ($\beta_{std} = -0.015$, $p = 0.001$), asthma ($\beta_{std} = -0.017$, $p = 0.002$), and emotional/behavioral problems ($\beta_{std} = -0.10$, $p < 0.001$) at 5 years of age (Figure 6A). These results support the hypothesis that the infant microbiota plays a crucial role in linking familial SES to NCD-associated childhood outcomes and underscore the importance of identifying and supporting specific beneficial microbes to promote healthy child development. Of the 30 microbial species linked to familial SES, eight were associated with elevated weight gain, three with asthma, and two with emotional or behavioral problems at age 5 (Figure 6B). Notably, *B. infantis* was the only species linked to two of the three SES-related outcomes, overweight and asthma, in a consistently protective direction (Figures 6B and S5C). It also showed a significant protective association with atopic sensitization.

Having identified *B. infantis* as an important species linked to SES-associated health outcomes, we next investigated the factors shaping its colonization and prevalence across populations. Although breastfeeding enriches *B. infantis*, it was detected in only 25% ($n = 352$ of 1,387) (Figures 6C and 6D) of the CHILD Study cohort during the first year, mirroring findings from other North American cohorts that report reduced colonization compared to non-industrialized countries.[33,34] To explore factors influencing colonization beyond breastfeeding, we leveraged the geographical diversity of the CHILD Study, which includes samples from four Canadian cities.

CellPress
OPEN ACCESS

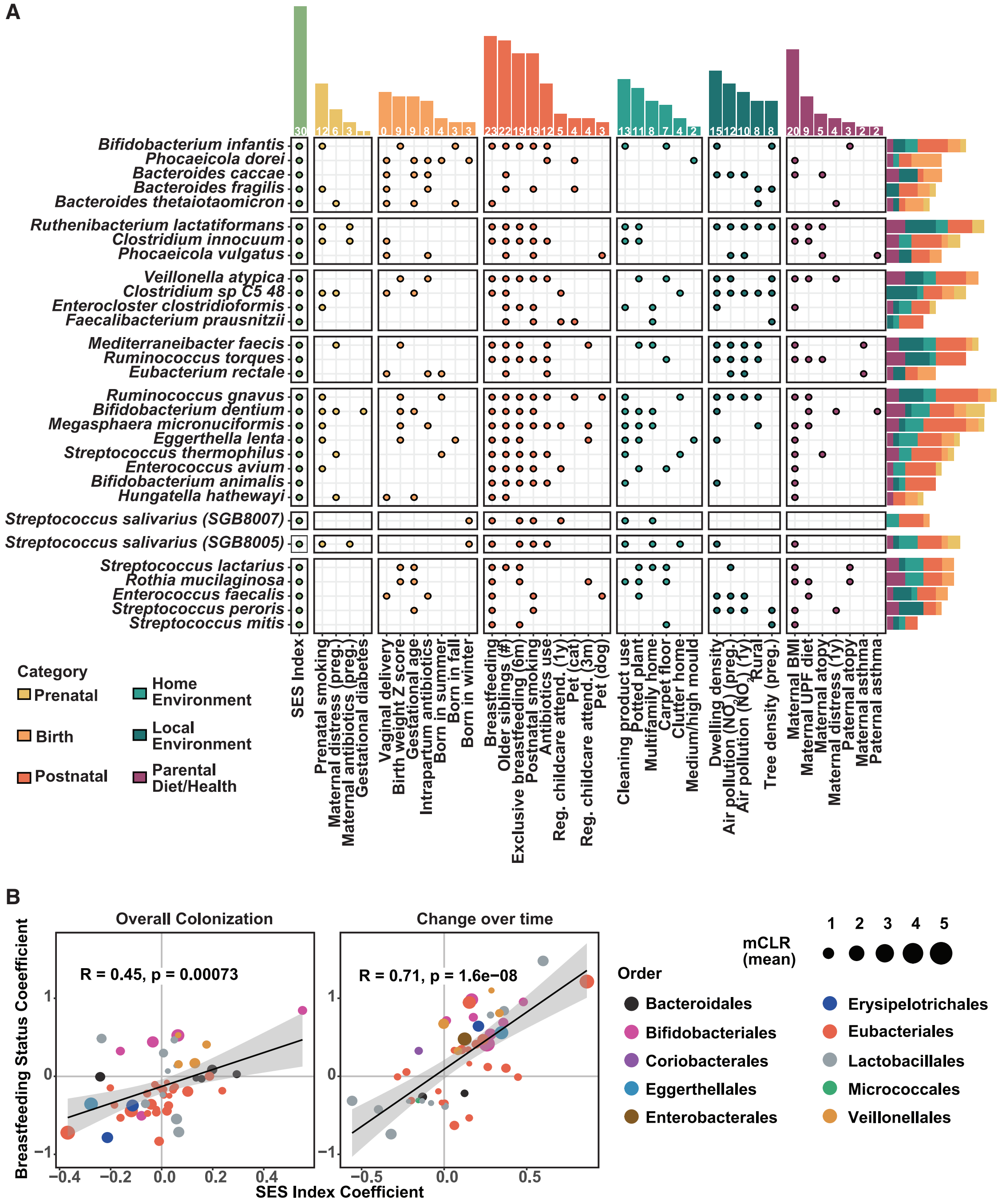

Figure 5. SES-associated species are linked to breastfeeding and other perinatal exposures

(A) Associations between perinatal factors and SES-associated species based on MaAslin2 models. Dots indicate significant associations (FDR <0.1) for either the slope or overall effect (STAR Methods), with colors denoting categories. The top bar shows the number of significant species per factor, and the right bar shows the number of significant factors per species. Species are grouped according to patterns of change across time and SES (Figure S4A). Child antibiotic use refers to exposure before stool collection, and breastfeeding refers to infants still breastfed at the time of sampling.

(B) Correlation plot of overall effects (MaAslin2 models without interaction measuring overall colonization) and slope effect (MaAslin2 models with interaction measuring change over time) for species significantly associated with SES or breastfeeding (FDR <0.1 for either effect). Dot color represents species family, and dot size represents mean relative abundance across 3 months and 1 year.

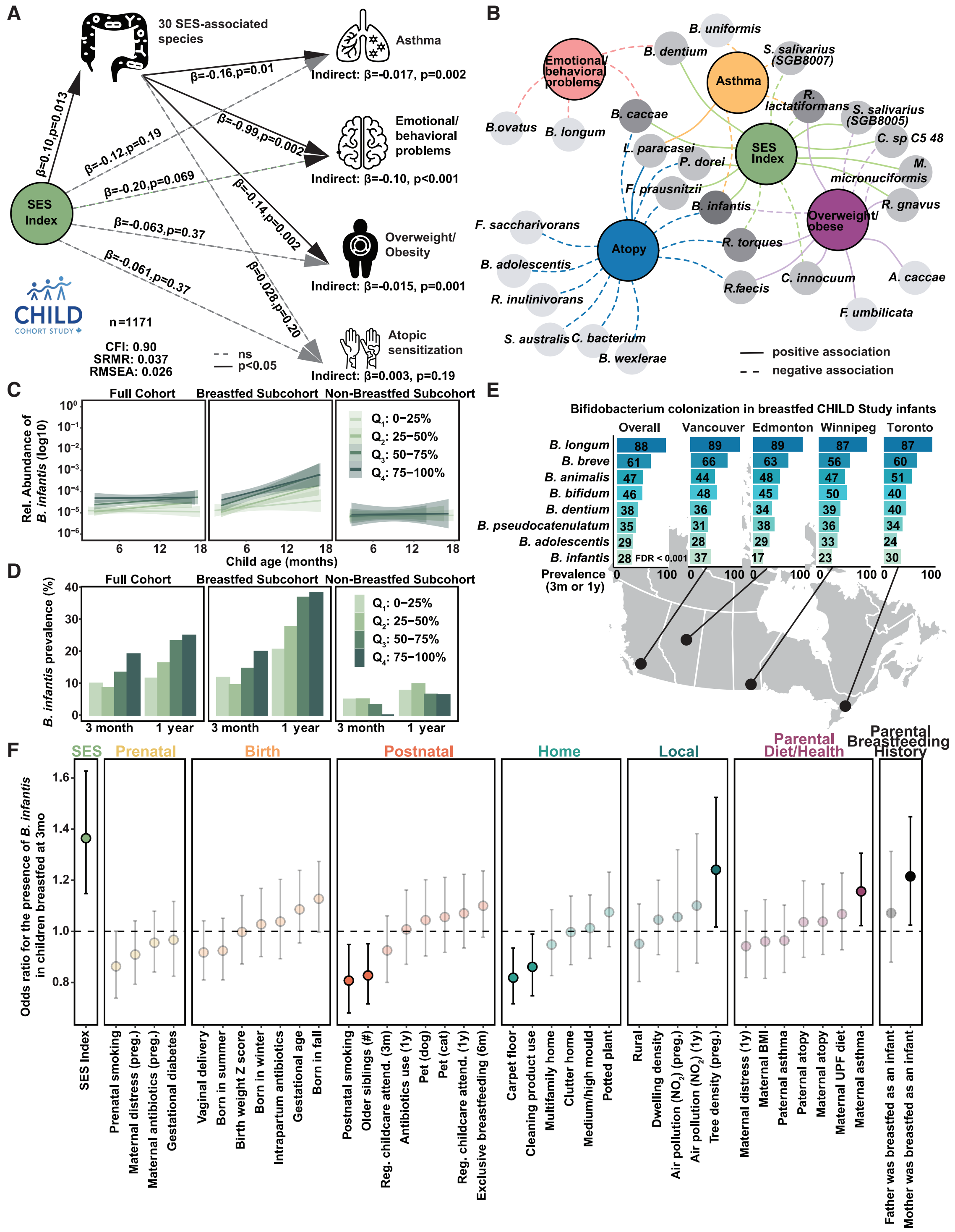

Figure 6. Higher family SES is associated with lower childhood overweight/obesity, asthma risk, and emotional/behavioral problems by shaping the infant gut microbiota in CHILD Study

(A) SEM testing mediation by SES-associated species at 3 months and 1 year on childhood outcomes at age 5 in the CHILD Study. Significant paths ($p < 0.05$) are solid black, and non-significant paths are gray dashed. Models were adjusted for sex, study site, stool collection age, and processing time.

(B) Species associated with any NCD risk factor (FDR<0.1) based on MaAslin2 slope effects or, if non-significant, overall effects. Solid lines indicate positive associations, and dashed lines indicate negative; darker shading reflects more associated phenotypes.

(legend continued on next page)

While 86% of infants were breastfed for at least 3 months, breastfeeding rates varied across sites (Figure S6A). Among these breastfed infants, *B. infantis* was the only *Bifidobacterium* species with significantly different prevalence (presence at either 3-month or 1-year visit) across cities (FDR<0.001, chi-square test), with Vancouver and Toronto showing the highest prevalence (37% and 30%) and Winnipeg and Edmonton the lowest (23% and 17%) (Figure 6E). These differences suggest that colonization is influenced by community-level factors beyond breastfeeding alone, possibly reflecting transmission influenced by the local microbiota meta-community.[35] Importantly, when we included Denmark in the analysis, we observed significant differences in the prevalence of several *Bifidobacterium* species in the first year of life (presence at either 1-month or 1-year visit) at the subject level among children breastfed up to 3 months (Table S3). One example is *B. infantis*, which showed lower prevalence in the CHILD cohort, but with higher relative abundance among children in whom it was detected (Table S4). This may reflect technical differences in sampling age or broader population differences between the North American and European cohorts, such as environmental exposures, healthcare practices, or community microbiota composition.[36,37] Regardless, within the Canadian birth cohort, *B. infantis* remained the only *Bifidobacterium* species with variable prevalence, underscoring its distinct role in early colonization patterns.

Both the abundance and prevalence of *B. infantis* were higher in breastfed infants during their first year than in those not breastfed (Figures 6C and 6D). However, even after accounting for breastfeeding, *B. infantis* remained more abundant in children from higher-SES families in both the CHILD and $COPSAC_{2010}$ cohorts (Figures S6B–S6E), suggesting additional influences on colonization. To further explore factors shaping *B. infantis* colonization, we used the breadth of perinatal and environmental data available from CHILD (Figure 6F). Beyond environmental exposures, we assessed parental breastfeeding history, offering insights into potential cross-generational effects. Four factors were negatively associated with *B. infantis* prevalence: postnatal smoking, number of older siblings, cleaning product use, and carpet flooring. In contrast, neighborhood tree density, maternal asthma, and maternal—but not paternal—breastfeeding history were positively associated. These results suggest that the enrichment of *B. infantis* may be supported by both breastfeeding and community-level exposures.

DISCUSSION

To effectively address environmental and lifestyle factors influencing disease, the scientific and medical communities must consider the compounded pressures of cultural, educational, and economic demands in industrialized societies.[38] This approach lays the foundation for reducing health disparities across all societal groups. In this study, we identify familial SES as a key driver of early-life exposures that shape microbiota composition and physiological development, setting the stage for long-term health trajectories. We defined SES using five measures, including income, educational attainment, and perceived social status, and found it was associated with more than half of the perinatal exposures infants in the CHILD Study encountered and associated with significant differences in the infant microbiota. This interplay was associated with health consequences ranging from asthma and overweight/obesity to behavioral challenges, illustrating how social and health inequities may emerge early in life.

The perinatal factors examined in this study encompassed parental health and diet, *in utero* exposures, early-life encounters during the first year, and conditions in both the home and broader environment. Each factor was linked to at least one health outcome at age 5 years, with many also associated with the infant gut microbiota as early as 3 months. Among these, breastfeeding emerged as the strongest associated factor, explaining much of the SES-associated differences in microbiota diversity and composition. Furthermore, breastfeeding may act as a buffer for the infant microbiota from environmental exposures, and the ability to exclusively breastfeed an infant for the first 6 months of life, in accordance with current global health recommendations, may provide protection from the development of NCD-associated childhood risk factors in lower-SES families. However, this protection appears less pronounced in children from higher-SES families. This pattern is consistent with additional differences in early-life exposures across socioeconomic contexts, including variation in prenatal and postnatal supports, access to healthcare, and exposures that affect the microbiota, such as antibiotics.[18] The influence of breastfeeding may be most evident when the infant microbiota experiences disruption. This aligns with our previous findings that breastfeeding itself was not associated with asthma risk overall but showed a protective effect among children exposed to antibiotics during the first year of life.[22] Breastfeeding is a highly conserved and adaptive behavior with extensive benefits for shaping both the microbiota and infant development, yet high-income countries have the lowest breastfeeding rates.[18,29,31,32,39] SES disparities in breastfeeding rates highlight modern challenges families face in maintaining breastfeeding, including pressure on parents to return to work quickly, and these challenges can be exacerbated by a lack of personal breastfeeding experience in previous generations.[40,41] Breastfeeding disparities, therefore, reflect a mismatch between societal and biological evolution, potentially contributing to the rise of NCDs in industrialized countries.[7,42] Our findings linking breastfeeding disparities to the rise of multiple NCD risk factors highlight a disproportionate burden

(C and D) (C) $\log_{10}$-transformed relative abundance and (D) prevalence of *B. infantis* among the full cohort, breastfed subcohort children (children breastfeeding at the time of stool sample collection) and non-breastfed subcohort (children were not breastfeeding by 3-month visit) in CHILD Study. Color indicates SES quartile.
(E) Prevalence of *Bifidobacterium* species, defined by presence in either 3-month or 1-year sample, across study sites among infants still breastfeeding at 3-month visit. Only *B. infantis* differed across sites (FDR<0.001, chi-square test).
(F) Odds ratio (95% CI) of perinatal factors and parental breastfeeding history associated with *B. infantis* colonization in the first year (defined by presence in either 3-month or 1-year sample), estimated using logistic regression adjusted for sex and study site. Colors denote factor categories; only significant associations ($p < 0.05$) are solid.

on families already experiencing social inequities and reaffirm arguments that the long-term health and economic benefits of breastfeeding are widely underestimated.[43] Addressing this issue requires societal-level policy and financial support, including generous paid parental leave, universal breastfeeding education and promotion in hospitals and birthing centers, and access to lactation specialists for ongoing support.[43,44] Furthermore, when breastfeeding is not clinically possible, improving access to donor human milk or developing human milk-inspired alternatives is essential to preserve the benefits of a human milk diet for infants and their developing microbiota.[45,46]

Our analysis of the infant microbiota revealed that breastfeeding-associated changes were associated with protection against multiple adverse health outcomes at age 5 years. Notably, higher levels of the species *B. infantis* were linked to reduced risk of atopic sensitization, asthma, and overweight/obesity. *B. infantis* has co-evolved with breastfeeding and is positively associated with several aspects of infant development.[47,48] However, it is present in only 25% of our cohort, a low prevalence echoed across North America, with some suggesting *B. infantis* loss may be a multi-generational consequence of industrialization.[33,34] Given the sensitivity of *B. infantis* to antibiotic exposure (Figures 5A and S4B), the combined rise in antibiotic use and formula feeding may have led to its reduction from our microbiota metacommunity over time, limiting its colonization even among breastfed infants. If this is the case, reducing barriers to a human milk diet in infancy should be complemented by the reintroduction of this important keystone species. While randomized controlled trials have already demonstrated some short-term benefits of supplementing *B. infantis* in breastfed neonates, the long-term impacts require further study and replicating stable *B. infantis* colonization and associated benefits in formula-fed infants remains a crucial step toward promoting equitable infant health.[49–51] Moreover, it is equally important to align public health efforts, such as antibiotic stewardship programs, that preserve *B. infantis* and other beneficial species within our communities. These efforts can help restore and maintain a resilient infant microbiota, supporting healthy development across society. While *B. infantis* is one species of interest in our study, we emphasize that it is not the only species that is associated with child health or solely mediating the observed SES-associated NCD risk. Rather, it is the collective contribution of the 30 SES-associated microbial species that significantly mediates these effects.

Limitations of the study

It is important to acknowledge the study's strengths as well as its limitations, including the fact that the CHILD Study skews toward urban families with higher income and education. CHILD Study's longitudinal, multi-center data includes comprehensive socioeconomic, perinatal, biological, and clinical data. This enabled us to associate a robust compound SES index with perinatal factors and multiple childhood NCD risk factors to identify broadly protective factors. Stool samples collected at 3-month and 1-year visits also support a longitudinal, multi-analytic approach to link SES and perinatal factors with the infant gut microbiota using large-scale shotgun metagenomic sequencing. Our main analyses used a derived compound SES index, integrating several observed SES factors collected from questionnaires, which may have distinct effects. To ensure robust results, we performed sensitivity analyses using subcohorts and individual SES factors, exploring associations through various methods. What's more, we replicated key findings in the independent $COPSAC_{2010}$ cohort, including the associations between SES, breastfeeding, and multiple childhood NCD risk factors; the mediating effect of breastfeeding on SES-associated infant gut microbiome; and the positive association between SES and *B. infantis*. While we found consistent effects of breastfeeding and SES on infant gut microbiota composition and individual taxonomy, we were limited to bacterial DNA profiling through shotgun sequencing. Moreover, we only profiled the gut microbiota, and we recognize the differences in the microbiota colonizing other body sites, including the lung and skin. In addition, we acknowledge that SES is associated with breastfeeding, which is a significant mediator of SES on infant gut microbiota. Thus, the estimated associations for SES and breastfeeding should be interpreted as partially overlapping rather than fully independent effects. Finally, the proportion of missing data for some health outcomes was modestly associated with SES, particularly for behavioral outcomes (Table S5), which could potentially introduce bias.

In conclusion, our findings demonstrate that familial SES significantly shapes the perinatal environment, influencing the infant gut microbiota and childhood health outcomes linked to NCD development. The protective association of SES was largely mediated by breastfeeding and the microbial species it supports, such as *B. infantis*. Our results highlight the critical role of breastfeeding and beneficial microbes in fostering healthy childhood growth and well-being, while also emphasizing the social and economic pressures families face. These findings advocate for structural changes that prioritize evolutionarily conserved mother-infant interactions and ensure their accessibility to all families. Specifically, our study supports reducing barriers to breastfeeding, continued research into microbe-based therapies, and public health initiatives aimed at maintaining and restoring the infant microbiota in an equitable manner.

RESOURCE AVAILABILITY

Lead contact

Further requests for information should be directed to and will be fulfilled by the lead contact, Stuart E. Turvey (sturvey@bcchr.ca).

Materials availability

This study did not generate new unique reagents.

Data and code availability

- The accession number for the shotgun metagenomic data reported in this paper is BioProject accession (NCBI): PRJNA838575. The informed consent obtained from the CHILD participants, in addition to the CHILD Inter-Institutional Agreement (IIA), which has been executed between the five Canadian institutions responsible for the study, govern the sharing of CHILD data. Data described in the manuscript are available by registration to the CHILD database (https://childstudy.ca/childdb/) and the submission of a formal request. All reasonable requests will be accommodated. More information about data access for the CHILD Cohort Study can be found at https://childstudy.ca/for-researchers/data-access/. Researchers interested in collaborating on a project and accessing CHILD Cohort Study data should contact child@mcmaster.

CellPress
OPEN ACCESS

ca. COPSAC sequencing data are available in the Sequence Read Archive (SRA) under accession no. PRJNA715601. Individual-level data are protected under Danish and European laws that prohibit publication even in pseudonymized form. However, data can be made available to researchers under a data processing agreement by contacting COPSAC's Data Protection Officer (administration@dbac.dk).

- Code for analyses and figures is provided in https://github.com/turveylab/Microbiome-SES-Breastfeeding-ChildHealth.
- Any additional information required to reanalyze the data reported in this work paper is available from the lead contact upon request.

ACKNOWLEDGMENTS

We are grateful to all the families who took part in this study and the whole CHILD team, which includes interviewers, nurses, computer and laboratory technicians, clerical workers, research scientists, volunteers, managers, and receptionists. We are grateful to the children and families who participated in the $COPSAC_{2010}$ cohort study for their dedication and support. Furthermore, we acknowledge and appreciate the unique efforts of all members of the COPSAC research team. S.E.T. holds a Tier 1 Canada Research Chair in Pediatric Precision Health and the Aubrey J. Tingle Professor of Pediatric Immunology. D.L.Y.D. is funded by a Canadian Institutes of Health Research Frederick Banting and Charles Best Canada Graduate Scholarship Doctoral Award (CIHR CGS-D) and the University of British Columbia Four Year Doctoral Fellowship (4YF). M.B.A. holds a Tier 2 Canada Research Chairs in Early Nutrition and the Developmental Origins of Health and Disease and is a Fellow of the CIFAR Humans and the Microbiome Program. P.S. holds a Tier 1 Canada Research Chair in Pediatric Asthma and Lung Health. P.J.M. holds funds from the Women's and Children's Health Research Institute. Funding for this specific study came from Genome Canada and Genome British Columbia (grant to S.E.T. [274CHI]), BC Children's Hospital Foundation, the Provincial Health Services Authority, and the Canadian Institutes of Health Research (grants to S.E.T. [OGB-198237 and OGB-185749]). COPSAC is supported by a variety of private and public research funds, listed on www.copsac.com.

AUTHOR CONTRIBUTIONS

Conceptualization, D.L.Y.D., C.P., and S.E.T.; methodology, D.L.Y.D. and C.P.; investigation and formal analysis, D.L.Y.D.; visualization, D.L.Y.D. and C.P.; funding acquisition, S.E.T., P.S., P.J.M., M.B.A., T.J.M., and E.S.; project administration, S.E.T. and P.S.; supervision, C.P. and S.E.T.; first draft, D.L.Y.D., C.P., and S.E.T.; review & editing, D.L.Y.D., M.B.M., C.H., J.J., H.S., K.M., S.C.C., D.J.K., Q.D., T.J.M., P.J.M., B.B.F., E.S., H.L., D.M.P., P.S., M.B.A., B.C., K.B., S.J.S., J.T., J.S. C.P., and S.E.T.; all authors agreed to submit the manuscript, read and approved the final draft, and take full responsibility of its content, including the accuracy of the data and their statistical analysis.

DECLARATION OF INTERESTS

M.B.A. has received speaker honoraria from Prolacta Biosciences (a human milk fortifier company), consulted for DSM Nutritional Products (a food ingredient company), and serves as an advisor for Tiny Health (an infant microbiome testing company).

STAR★METHODS

Detailed methods are provided in the online version of this paper and include the following:

- KEY RESOURCES TABLE
- EXPERIMENTAL MODEL AND STUDY PARTICIPANT DETAILS
  - CHILD study
  - $COPSAC_{2010}$ cohort
  - Ethics and data access
- METHOD DETAILS
- QUANTIFICATION AND STATISTICAL ANALYSIS

SUPPLEMENTAL INFORMATION

Supplemental information can be found online at https://doi.org/10.1016/j.xcrm.2026.102755.

Received: July 14, 2025
Revised: December 27, 2025
Accepted: March 19, 2026
Published: April 13, 2026

REFERENCES

1. Braveman, P.A., Cubbin, C., Egerter, S., Williams, D.R., and Pamuk, E. (2010). Socioeconomic disparities in health in the United States: what the patterns tell us. Am. J. Public Health *100*, S186–S196. https://doi.org/10.2105/AJPH.2009.166082.
2. Ismail, S.U., Asamane, E.A., Osei-Kwasi, H.A., and Boateng, D. (2022). Socioeconomic Determinants of Cardiovascular Diseases, Obesity, and Diabetes among Migrants in the United Kingdom: A Systematic Review. Int. J. Environ. Res. Public Health *19*, 3070. https://doi.org/10.3390/ijerph19053070.
3. Reiss, F., Meyrose, A.K., Otto, C., Lampert, T., Klasen, F., and Ravens-Sieberer, U. (2019). Socioeconomic status, stressful life situations and mental health problems in children and adolescents: Results of the German BELLA cohort-study. PLoS One *14*, e0213700. https://doi.org/10.1371/journal.pone.0213700.
4. Ellison-Loschmann, L., Sunyer, J., Plana, E., Pearce, N., Zock, J.P., Jarvis, D., Janson, C., Antó, J.M., and Kogevinas, M.; European Community Respiratory Health Survey (2007). Socioeconomic status, asthma and chronic bronchitis in a large community-based study. Eur. Respir. J. *29*, 897–905. https://doi.org/10.1183/09031936.00101606.
5. Poulain, T., Vogel, M., and Kiess, W. (2020). Review on the role of socioeconomic status in child health and development. Curr. Opin. Pediatr. *32*, 308–314. https://doi.org/10.1097/MOP.0000000000000876.
6. Forrest, C.B., Koenigsberg, L.J., Eddy Harvey, F., Maltenfort, M.G., and Halfon, N. (2025). Trends in US Children's Mortality, Chronic Conditions, Obesity, Functional Status, and Symptoms. JAMA *334*, 509–516. https://doi.org/10.1001/jama.2025.9855.
7. Corbett, S., Courtiol, A., Lummaa, V., Moorad, J., and Stearns, S. (2018). The transition to modernity and chronic disease: mismatch and natural selection. Nat. Rev. Genet. *19*, 419–430. https://doi.org/10.1038/s41576-018-0012-3.
8. Finlay, B.B.; CIFAR Humans, and MicCiome (2020). Are noncommunicable diseases communicable? Science *367*, 250–251. https://doi.org/10.1126/science.aaz3834.
9. Amato, K.R., Arrieta, M.C., Azad, M.B., Bailey, M.T., Broussard, J.L., Bruggeling, C.E., Claud, E.C., Costello, E.K., Davenport, E.R., Dutilh, B.E., et al. (2021). The human gut microbiome and health inequities. Proc. Natl. Acad. Sci. USA *118*, e2017947118. https://doi.org/10.1073/pnas.2017947118.
10. Dowd, J.B., and Renson, A. (2018). "Under the Skin" and into the Gut: Social Epidemiology of the Microbiome. Curr. Epidemiol. Rep. *5*, 432–441. https://doi.org/10.1007/s40471-018-0167-7.
11. Sonnenburg, J.L., and Sonnenburg, E.D. (2019). Vulnerability of the industrialized microbiota. Science *366*, eaaw9255. https://doi.org/10.1126/science.aaw9255.
12. Vangay, P., Johnson, A.J., Ward, T.L., Al-Ghalith, G.A., Shields-Cutler, R.R., Hillmann, B.M., Lucas, S.K., Beura, L.K., Thompson, E.A., Till, L.M., et al. (2018). US Immigration Westernizes the Human Gut Microbiome. Cell *175*, 962–972.e10. https://doi.org/10.1016/j.cell.2018.10.029.

13. Sonnenburg, E.D., and Sonnenburg, J.L. (2019). The ancestral and industrialized gut microbiota and implications for human health. Nat. Rev. Microbiol. *17*, 383–390. https://doi.org/10.1038/s41579-019-0191-8.

14. Sarkar, A., McInroy, C.J.A., Harty, S., Raulo, A., Ibata, N.G.O., Valles-Colomer, M., Johnson, K.V.A., Brito, I.L., Henrich, J., Archie, E.A., et al. (2024). Microbial transmission in the social microbiome and host health and disease. Cell *187*, 17–43. https://doi.org/10.1016/j.cell.2023.12.014.

15. Miller, E.T., Svanbäck, R., and Bohannan, B.J.M. (2018). Microbiomes as Metacommunities: Understanding Host-Associated Microbes through Metacommunity Ecology. Trends Ecol. Evol. *33*, 926–935. https://doi.org/10.1016/j.tree.2018.09.002.

16. Prescott, S.L. (2020). A butterfly flaps its wings: Extinction of biological experience and the origins of allergy. Ann. Allergy Asthma Immunol. *125*, 528–534. https://doi.org/10.1016/j.anai.2020.05.025.

17. Bello, M.G.D., Knight, R., Gilbert, J.A., and Blaser, M.J. (2018). Preserving microbial diversity. Science *362*, 33–34. https://doi.org/10.1126/science.aau8816.

18. Dai, D.L.Y., Petersen, C., and Turvey, S.E. (2024). Reduce, reinforce, and replenish: safeguarding the early-life microbiota to reduce intergenerational health disparities. Front. Public Health *12*, 1455503. https://doi.org/10.3389/fpubh.2024.1455503.

19. Christian, V.J., Miller, K.R., and Martindale, R.G. (2020). Food Insecurity, Malnutrition, and the Microbiome. Curr. Nutr. Rep. *9*, 356–360. https://doi.org/10.1007/s13668-020-00342-0.

20. Petersen, C., and Turvey, S.E. (2020). Can we prevent allergic disease? Understanding the links between the early life microbiome and allergic diseases of childhood. Curr. Opin. Pediatr. *32*, 790–797. https://doi.org/10.1097/mop.0000000000000956.

21. Donald, K., and Finlay, B.B. (2023). Early-life interactions between the microbiota and immune system: impact on immune system development and atopic disease. Nat. Rev. Immunol. *23*, 735–748. https://doi.org/10.1038/s41577-023-00874-w.

22. Dai, D.L.Y., Petersen, C., Hoskinson, C., Del Bel, K.L., Becker, A.B., Moraes, T.J., Mandhane, P.J., Finlay, B.B., Simons, E., Kozyrskyj, A.L., et al. (2023). Breastfeeding enrichment of B. longum subsp. infantis mitigates the effect of antibiotics on the microbiota and childhood asthma risk. Med *4*, 92–112.e5. https://doi.org/10.1016/j.medj.2022.12.002.

23. Hoskinson, C., Dai, D.L.Y., Del Bel, K.L., Becker, A.B., Moraes, T.J., Mandhane, P.J., Finlay, B.B., Simons, E., Kozyrskyj, A.L., Azad, M.B., et al. (2023). Delayed gut microbiota maturation in the first year of life is a hallmark of pediatric allergic disease. Nat. Commun. *14*, 4785. https://doi.org/10.1038/s41467-023-40336-4.

24. Amaruddin, A.I., Hamid, F., Koopman, J.P.R., Muhammad, M., Brienen, E.A., van Lieshout, L., Geelen, A.R., Wahyuni, S., Kuijper, E.J., Sartono, E., et al. (2020). The Bacterial Gut Microbiota of Schoolchildren from High and Low Socioeconomic Status: A Study in an Urban Area of Makassar, Indonesia. Microorganisms *8*, 961. https://doi.org/10.3390/microorganisms8060961.

25. Lewis, C.R., Bonham, K.S., McCann, S.H., Volpe, A.R., D'Sa, V., Naymik, M., De Both, M.D., Huentelman, M.J., Lemery-Chalfant, K., Highlander, S.K., et al. (2021). Family SES Is Associated with the Gut Microbiome in Infants and Children. Microorganisms *9*, 1608. https://doi.org/10.3390/microorganisms9081608.

26. Lapidot, Y., Reshef, L., Maya, M., Cohen, D., Gophna, U., and Muhsen, K. (2022). Socioeconomic disparities and household crowding in association with the fecal microbiome of school-age children. NPJ Biofilms Microbiomes *8*, 10. https://doi.org/10.1038/s41522-022-00271-6.

27. Subbarao, P., Anand, S.S., Becker, A.B., Befus, A.D., Brauer, M., Brook, J.R., Denburg, J.A., HayGlass, K.T., Kobor, M.S., Kollmann, T.R., et al. (2015). The Canadian Healthy Infant Longitudinal Development (CHILD) Study: examining developmental origins of allergy and asthma. Thorax *70*, 998–1000. https://doi.org/10.1136/thoraxjnl-2015-207246.

28. Adler, N.E., Epel, E.S., Castellazzo, G., and Ickovics, J.R. (2000). Relationship of subjective and objective social status with psychological and physiological functioning: preliminary data in healthy white women. Health Psychol. *19*, 586–592. https://doi.org/10.1037//0278-6133.19.6.586.

29. Shenhav, L., Fehr, K., Reyna, M.E., Petersen, C., Dai, D.L.Y., Dai, R., Breton, V., Rossi, L., Smieja, M., Simons, E., et al. (2024). Microbial colonization programs are structured by breastfeeding and guide healthy respiratory development. Cell *187*, 5431–5452.e20. https://doi.org/10.1016/j.cell.2024.07.022.

30. Gacesa, R., Kurilshikov, A., Vich Vila, A., Sinha, T., Klaassen, M.A.Y., Bolte, L.A., Andreu-Sánchez, S., Chen, L., Collij, V., Hu, S., et al. (2022). Environmental factors shaping the gut microbiome in a Dutch population. Nature *604*, 732–739. https://doi.org/10.1038/s41586-022-04567-7.

31. Fehr, K., Moossavi, S., Sbihi, H., Boutin, R.C.T., Bode, L., Robertson, B., Yonemitsu, C., Field, C.J., Becker, A.B., Mandhane, P.J., et al. (2020). Breastmilk Feeding Practices Are Associated with the Co-Occurrence of Bacteria in Mothers' Milk and the Infant Gut: the CHILD Cohort Study. Cell Host Microbe *28*, 285–297.e4. https://doi.org/10.1016/j.chom.2020.06.009.

32. Yang, R., Gao, R., Cui, S., Zhong, H., Zhang, X., Chen, Y., Wang, J., and Qin, H. (2019). Dynamic signatures of gut microbiota and influences of delivery and feeding modes during the first 6 months of life. Physiol. Genomics *51*, 368–378. https://doi.org/10.1152/physiolgenomics.00026.2019.

33. Seppo, A.E., Bu, K., Jumabaeva, M., Thakar, J., Choudhury, R.A., Yonemitsu, C., Bode, L., Martina, C.A., Allen, M., Tamburini, S., et al. (2021). Infant gut microbiome is enriched with Bifidobacterium longum ssp. infantis in Old Order Mennonites with traditional farming lifestyle. Allergy *76*, 3489–3503. https://doi.org/10.1111/all.14877.

34. Casaburi, G., Duar, R.M., Brown, H., Mitchell, R.D., Kazi, S., Chew, S., Cagney, O., Flannery, R.L., Sylvester, K.G., Frese, S.A., et al. (2021). Metagenomic insights of the infant microbiome community structure and function across multiple sites in the United States. Sci. Rep. *11*, 1472. https://doi.org/10.1038/s41598-020-80583-9.

35. Taft, D.H., Lewis, Z.T., Nguyen, N., Ho, S., Masarweh, C., Dunne-Castagna, V., Tancredi, D.J., Huda, M.N., Stephensen, C.B., Hinde, K., et al. (2022). Bifidobacterium Species Colonization in Infancy: A Global Cross-Sectional Comparison by Population History of Breastfeeding. Nutrients *14*, 1423. https://doi.org/10.3390/nu14071423.

36. Lu, J., Zhang, L., Zhang, H., Chen, Y., Zhao, J., Chen, W., Lu, W., and Li, M. (2023). Population-level variation in gut bifidobacterial composition and association with geography, age, ethnicity, and staple food. NPJ Biofilms Microbiomes *9*, 98. https://doi.org/10.1038/s41522-023-00467-4.

37. Yatsunenko, T., Rey, F.E., Manary, M.J., Trehan, I., Dominguez-Bello, M.G., Contreras, M., Magris, M., Hidalgo, G., Baldassano, R.N., Anokhin, A.P., et al. (2012). Human gut microbiome viewed across age and geography. Nature *486*, 222–227. https://doi.org/10.1038/nature11053.

38. Manderson, L., and Jewett, S. (2023). Risk, lifestyle and non-communicable diseases of poverty. Global. Health *19*, 13. https://doi.org/10.1186/s12992-023-00914-z.

39. Victora, C.G., Bahl, R., Barros, A.J.D., França, G.V.A., Horton, S., Krasevec, J., Murch, S., Sankar, M.J., Walker, N., and Rollins, N.C.; Lancet Breastfeeding Series Group (2016). Breastfeeding in the 21st century: epidemiology, mechanisms, and lifelong effect. Lancet *387*, 475–490. https://doi.org/10.1016/S0140-6736(15)01024-7.

40. Negin, J., Coffman, J., Vizintin, P., and Raynes-Greenow, C. (2016). The influence of grandmothers on breastfeeding rates: a systematic review. BMC Pregnancy Childbirth *16*, 91. https://doi.org/10.1186/s12884-016-0880-5.

41. Di Manno, L., Macdonald, J.A., and Knight, T. (2015). The intergenerational continuity of breastfeeding intention, initiation, and duration: a systematic review. Birth *42*, 5–15. https://doi.org/10.1111/birt.12148.

42. Lea, A.J., Clark, A.G., Dahl, A.W., Devinsky, O., Garcia, A.R., Golden, C.D., Kamau, J., Kraft, T.S., Lim, Y.A.L., Martins, D.J., et al. (2023). Applying an

CellPress
OPEN ACCESS

evolutionary mismatch framework to understand disease susceptibility. PLoS Biol. *21*, e3002311. https://doi.org/10.1371/journal.pbio.3002311.

43. Rollins, N.C., Bhandari, N., Hajeebhoy, N., Horton, S., Lutter, C.K., Martines, J.C., Piwoz, E.G., Richter, L.M., and Victora, C.G.; Lancet Breastfeeding Series Group (2016). Why invest, and what it will take to improve breastfeeding practices? Lancet *387*, 491–504. https://doi.org/10.1016/S0140-6736(15)01044-2.

44. Diaz, L.E., Yee, L.M., and Feinglass, J. (2023). Rates of breastfeeding initiation and duration in the United States: data insights from the 2016-2019 Pregnancy Risk Assessment Monitoring System. Front. Public Health *11*, 1256432. https://doi.org/10.3389/fpubh.2023.1256432.

45. Bai, Y., and Kuscin, J. (2021). The Current State of Donor Human Milk Use and Practice. J. Midwifery Womens Health *66*, 478–485. https://doi.org/10.1111/jmwh.13244.

46. Xu, L.L., and Townsend, S.D. (2021). Synthesis as an Expanding Resource in Human Milk Science. J. Am. Chem. Soc. *143*, 11277–11290. https://doi.org/10.1021/jacs.1c05599.

47. Sela, D.A., Chapman, J., Adeuya, A., Kim, J.H., Chen, F., Whitehead, T.R., Lapidus, A., Rokhsar, D.S., Lebrilla, C.B., German, J.B., et al. (2008). The genome sequence of Bifidobacterium longum subsp. infantis reveals adaptations for milk utilization within the infant microbiome. Proc. Natl. Acad. Sci. USA *105*, 18964–18969. https://doi.org/10.1073/pnas.0809584105.

48. Laursen, M.F., Sakanaka, M., von Burg, N., Mörbe, U., Andersen, D., Moll, J.M., Pekmez, C.T., Rivollier, A., Michaelsen, K.F., Mølgaard, C., et al. (2021). Bifidobacterium species associated with breastfeeding produce aromatic lactic acids in the infant gut. Nat. Microbiol. *6*, 1367–1382. https://doi.org/10.1038/s41564-021-00970-4.

49. Capeding, M.R.Z., Phee, L.C.M., Ming, C., Noti, M., Vidal, K., Le Carrou, G., Frézal, A., Moll, J.M., Vogt, J.K., Myers, P.N., et al. (2023). Safety, efficacy, and impact on gut microbial ecology of a Bifidobacterium longum subspecies infantis LMG11588 supplementation in healthy term infants: a randomized, double-blind, controlled trial in the Philippines. Front. Nutr. *10*, 1319873. https://doi.org/10.3389/fnut.2023.1319873.

50. O'Brien, C.E., Meier, A.K., Cernioglo, K., Mitchell, R.D., Casaburi, G., Frese, S.A., Henrick, B.M., Underwood, M.A., and Smilowitz, J.T. (2022). Early probiotic supplementation with B. infantis in breastfed infants leads to persistent colonization at 1 year. Pediatr. Res. *91*, 627–636. https://doi.org/10.1038/s41390-020-01350-0.

51. Smilowitz, J.T., Moya, J., Breck, M.A., Cook, C., Fineberg, A., Angkustsiri, K., and Underwood, M.A. (2017). Safety and tolerability of Bifidobacterium longum subspecies infantis EVC001 supplementation in healthy term breastfed infants: a phase I clinical trial. BMC Pediatr. *17*, 133. https://doi.org/10.1186/s12887-017-0886-9.

52. McMurdie, P.J., and Holmes, S. (2013). phyloseq: An R Package for Reproducible Interactive Analysis and Graphics of Microbiome Census Data. PLoS One *8*, e61217. https://doi.org/10.1371/journal.pone.0061217.

53. Beghini, F., McIver, L.J., Blanco-Míguez, A., Dubois, L., Asnicar, F., Maharjan, S., Mailyan, A., Manghi, P., Scholz, M., Thomas, A.M., et al. (2021). Integrating taxonomic, functional, and strain-level profiling of diverse microbial communities with bioBakery 3. eLife *10*, e65088. https://doi.org/10.7554/eLife.65088.

54. Mallick, H., Rahnavard, A., McIver, L.J., Ma, S., Zhang, Y., Nguyen, L.H., Tickle, T.L., Weingart, G., Ren, B., Schwager, E.H., et al. (2021). Multivariable association discovery in population-scale meta-omics studies. PLoS Comput. Biol. *17*, e1009442. https://doi.org/10.1371/journal.pcbi.1009442.

55. Rosseel, Y. (2012). lavaan: An R Package for Structural Equation Modeling. J. Stat. Softw. *48*, 1–36. https://doi.org/10.18637/jss.v048.i02.

56. Revelle, W. (2013). Psych: Procedures for Psychological, Psychometric, and Personality Research (Evanston, Illinois: R Package Version 1.0–95).

57. Yoon, G., Gaynanova, I., and Müller, C.L. (2019). Microbial Networks in SPRING - Semi-parametric Rank-Based Correlation and Partial Correlation Estimation for Quantitative Microbiome Data. Front. Genet. *10*, 516. https://doi.org/10.3389/fgene.2019.00516.

58. Anderson, M.J. (2001). A new method for non-parametric multivariate analysis of variance. Austral Ecol. *26*, 32–46. https://doi.org/10.1111/j.1442-9993.2001.01070.pp.x.

59. World Health, O., Onís, M.d., and World Health, O. (2006). WHO Child Growth Standards : Length/height-For-Age, Weight-For-Age, Weight-For-Length, Weight-For-Height and Body Mass Index-For-Age : Methods and Development, 1st Edition (World Health Organization).

60. Reyna, M.E., Petersen, C., Dai, D.L.Y., Dai, R., Becker, A.B., Azad, M.B., Miliku, K., Lefebvre, D.L., Moraes, T.J., Mandhane, P.J., et al. (2022). Longitudinal body mass index trajectories at preschool age: children with rapid growth have differential composition of the gut microbiota in the first year of life. Int. J. Obes. *46*, 1351–1358. https://doi.org/10.1038/s41366-022-01117-z.

61. Achenbach, T.M. (1999). The Child Behavior Checklist and related instruments. In The use of psychological testing for treatment planning and outcomes assessment, 2nd ed. (Lawrence Erlbaum Associates Publishers), pp. 429–466.

62. Cohen, S., Kamarck, T., and Mermelstein, R. (1983). A global measure of perceived stress. J. Health Soc. Behav. *24*, 385–396.

63. Kramer, M.S., Platt, R.W., Wen, S.W., Joseph, K.S., Allen, A., Abrahamowicz, M., Blondel, B., and Bréart, G.; Fetal/Infant Health Study Group of the Canadian Perinatal Surveillance System (2001). A new and improved population-based Canadian reference for birth weight for gestational age. Pediatrics *108*, E35. https://doi.org/10.1542/peds.108.2.e35.

64. Parks, J., McCandless, L., Dharma, C., Brook, J., Turvey, S.E., Mandhane, P., Becker, A.B., Kozyrskyj, A.L., Azad, M.B., Moraes, T.J., et al. (2020). Association of use of cleaning products with respiratory health in a Canadian birth cohort. CMAJ (Can. Med. Assoc. J.) *192*, E154–E161. https://doi.org/10.1503/cmaj.190819.

65. Sbihi, H., Allen, R.W., Becker, A., Brook, J.R., Mandhane, P., Scott, J.A., Sears, M.R., Subbarao, P., Takaro, T.K., Turvey, S.E., and Brauer, M. (2015). Perinatal Exposure to Traffic-Related Air Pollution and Atopy at 1 Year of Age in a Multi-Center Canadian Birth Cohort Study. Environ. Health Perspect. *123*, 902–908. https://doi.org/10.1289/ehp.1408700.

66. Moossavi, S., Fehr, K., Derakhshani, H., Sbihi, H., Robertson, B., Bode, L., Brook, J., Turvey, S.E., Moraes, T.J., Becker, A.B., et al. (2020). Human milk fungi: environmental determinants and inter-kingdom associations with milk bacteria in the CHILD Cohort Study. BMC Microbiol. *20*, 146. https://doi.org/10.1186/s12866-020-01829-0.

67. Monteiro, C.A., Cannon, G., Levy, R.B., Moubarac, J.-C., Jaime, P.C., Martins, A.P.B., Canella, D.S., Louzada, M.L.d.C., and Parra, D.C. (2016). NOVA. The star shines bright. World nutrition *7*, 28–38. https://www.worldnutritionjournal.org/index.php/wn/article/view/5.

68. Ambalavanan, A., Chang, L., Choi, J., Zhang, Y., Stickley, S.A., Fang, Z.Y., Miliku, K., Robertson, B., Yonemitsu, C., Turvey, S.E., et al. (2024). Human milk oligosaccharides are associated with maternal genetics and respiratory health of human milk-fed children. Nat. Commun. *15*, 7735. https://doi.org/10.1038/s41467-024-51743-6.

69. Bisgaard, H., Vissing, N.H., Carson, C.G., Bischoff, A.L., Følsgaard, N.V., Kreiner-Møller, E., Chawes, B.L.K., Stokholm, J., Pedersen, L., Bjarnadóttir, E., et al. (2013). Deep phenotyping of the unselected COPSAC2010 birth cohort study. Clin. Exp. Allergy *43*, 1384–1394. https://doi.org/10.1111/cea.12213.

70. Bisgaard, H., Stokholm, J., Chawes, B.L., Vissing, N.H., Bjarnadóttir, E., Schoos, A.-M.M., Wolsk, H.M., Pedersen, T.M., Vinding, R.K., Thorsteinsdóttir, S., et al. (2016). Fish Oil–Derived Fatty Acids in Pregnancy and Wheeze and Asthma in Offspring. N. Engl. J. Med. *375*, 2530–2539. https://doi.org/10.1056/NEJMoa1503734.

71. Myatt, M., and Guevarra, E. (2019). Zscorer: Child Anthropometry Z-Score Calculator (Vienna, Austria: R Foundation), p. 1.

72. Bisgaard, H., Hermansen, M.N., Loland, L., Halkjaer, L.B., and Buchvald, F. (2006). Intermittent inhaled corticosteroids in infants with episodic wheezing. N. Engl. J. Med. *354*, 1998–2005. https://doi.org/10.1056/NEJMoa054692.

73. Stokholm, J., Thorsen, J., Blaser, M.J., Rasmussen, M.A., Hjelmsø, M., Shah, S., Christensen, E.D., Chawes, B.L., Bønnelykke, K., Brix, S., et al. (2020). Delivery mode and gut microbial changes correlate with an increased risk of childhood asthma. Sci. Transl. Med. *12*, eaax9929. https://doi.org/10.1126/scitranslmed.aax9929.

74. Schoos, A.M.M., Hansen, B.R., Stokholm, J., Chawes, B.L., Bønnelykke, K., and Bisgaard, H. (2020). Parent-specific effects on risk of developing allergic sensitization and asthma in childhood. Clin. Exp. Allergy *50*, 915–921. https://doi.org/10.1111/cea.13670.

75. Bryant, A., Guy, J., and CALM Team; and Holmes, J. (2020). The Strengths and Difficulties Questionnaire Predicts Concurrent Mental Health Difficulties in a Transdiagnostic Sample of Struggling Learners. Front. Psychol. *11*, 587821. https://doi.org/10.3389/fpsyg.2020.587821.

76. Ennis, D., Shmorak, S., Jantscher-Krenn, E., and Yassour, M. (2024). Longitudinal quantification of Bifidobacterium longum subsp. infantis reveals late colonization in the infant gut independent of maternal milk HMO composition. Nat. Commun. *15*, 894. https://doi.org/10.1038/s41467-024-45209-y.

77. Langmead, B., and Salzberg, S.L. (2012). Fast gapped-read alignment with Bowtie 2. Nat. Methods *9*, 357–359. https://doi.org/10.1038/nmeth.1923.

78. Hair, J.F., Black, W.C., and Babin, B.J. (2010). Multivariate Data Analysis: A Global Perspective (Pearson Education).

CellPress
OPEN ACCESS

STAR★METHODS

KEY RESOURCES TABLE

REAGENT or RESOURCE	SOURCE	IDENTIFIER
Biological samples		
Stool samples	Dai, D.L.Y. et al.[22]	CHILD study http://childstudy.ca https://www.ncbi.nlm.nih.gov/bioproject/838575
Deposited data		
CHILD shotgun metagenomic data	BioProject accession (NCBI)	PRJNA838575
COPSAC shotgun metagenomic data	Sequence Read Archive (SRA)	PRJNA715601
Software and algorithms		
R v. 4.4.0	R Core Team	https://www.r-project.org
Phyloseq v. 1.48.0	McMurdie, P.J. et al.[52]	https://joey711.github.io/phyloseq/index.html
bioBakery 3	Ho, N.T. et al.[53]	https://github.com/biobakery/biobakery
MaAsLin2 v. 1.18.0	Mallick, H. et al.[54]	https://github.com/biobakery/Maaslin2
lavaan v. 0.6.19	Rosseel, Y. et al.[55]	https://www.jstatsoft.org/article/view/v048i02
psych v. 2.4.6.26	Revelle, W.[56]	https://cran.r-project.org/web/packages/psych/index.html
metaMint v. 0.1.0	Yoon, G.[57]	https://rdrr.io/github/drjingma/metaMINT/
vegan v. 2.6.8	Anderson, M. J.[58]	https://cran.r-project.org/web/packages/vegan/index.html

EXPERIMENTAL MODEL AND STUDY PARTICIPANT DETAILS

CHILD study

Characteristics of the study population

The CHILD study is a prospective longitudinal birth cohort study, which enrolled 3,405 subjects since pregnancy from 4 largely urban study centers across Canada (Vancouver, Edmonton, Winnipeg, and Toronto) from 2008 to 2012.[27] Our study included 3,263 subjects eligible at birth (Figure S1A), who were born at a minimum of 34 weeks of gestation and had no congenital abnormalities. CHILD Study followed children prospectively and collected detailed information on environmental exposures and clinical outcomes using a combination of questionnaires and in-person clinical assessments. Questionnaires related to environmental exposures, psychosocial stresses, nutrition and general health were administered at recruitment, prenatally, at 3, 6, 12, 18, 24, 30 months, and at 3, 4, and 5 years.

Socioeconomic status

In this study, we used the 2 continuous and 3 ordered categorical socioeconomic factors collected from questionnaires fulfilled by parents during pregnancy at 18 weeks and 1 year postnatal, which were the highest education of father and mother (4 ordinal levels: 1 = complete high school, 2 = complete college, 3 = complete university, 4 = master or PhD), annual household income (4 ordinal levels in Canadian Dollars: 1 = 0–49999, 2 = 50000–99999, 3 = 100000–149999, 4 = over 150000), and continuous MacArthur scale of subjective social status score in Canada and community, where parents put themselves on the ladder, ranging from 1 to 10, of which 1 is the lowest and 10 is the highest. A total of 2,752 children had complete socioeconomic status data.

Early risk factors for multiple NCDs

In our study, adverse health outcomes collected at the 5-year visit were considered to be early risk factors for NCDs, including overweight or obesity, physician diagnosis of asthma, atopic sensitization and emotional/behavioral problems.

Childhood overweight/obese. The weight and height measurements of children were performed by trained research assistants at clinic visits when children were 5 years old. Shoes and outerwear were removed for weight (Scaletronix scale) and height (standard stadiometer) measurements. BMI was calculated and age and sex BMI *Z* score (BMIz) was derived according to the World Health Organization (WHO) child growth standards[59] for children younger than 5 years, and for 5–19 years for those children that were slightly older than 5 years old at the date of measurement. Children were classified as underweight with BMI z-scores less than -1, normal from -1 to 1, overweight from 1 to 2, and obese over 2.[60]

Childhood asthma. Childhood asthma was diagnosed (as Yes/Possible/No) by an expert study physician at the clinical assessment at the age of 5 years based on the CHILD Study's published approach.[27] For this study, children were considered to have asthma only

if the response was 'Yes' and the asthma phenotype was defined as comparing children with asthma (n = 165) at 5 years versus children without asthma (n = 2234) at 5 years, children diagnosed as "possible" (n = 247) were excluded.

Childhood atopic sensitization. Children enrolled in the CHILD study were administered a skin prick testing (SPT) at the 5-year scheduled visit. Children were then diagnosed with IgE-mediated allergic sensitization (also referred to as atopy) based on SPT to multiple common foods and environmental inhalant allergens, using $\geq$2 mm average wheal size as indicating a positive test relative to the negative control. The allergens tested at 5-year visits include cat hair, the German cockroach, Alternaria tenuis, house dust mites, dog epithelium, cow's milk, peanut, egg white, soybean, *Cladosporium*, *Penicillium*, *Aspergillus fumigatus*, trees, grass, weeds, and ragweed. Glycerin and histamine served as the negative and positive controls, respectively.[23]

Childhood emotional/behavioral problems. Childhood emotional/behavioral problems is defined based on Children Child Behavior Checklist (CBCL),[61] which is a widely used and validated questionnaire to assess behavioral and emotional problems. Parents report on the frequency of child problems or behaviors over the last 6 months using a 3-point Likert scale. Higher scores indicate greater symptoms. In this study, children with CBCL T-score >60, the borderline elevation, in either externalizing or internalizing problems at 5 years of age were defined as children with emotional/behavioral problems.

Perinatal factors and exposomes

Pregnancy. Relevant exposures during pregnancy collected by the CHILD study included gestational diabetes during pregnancy, maternal distress during pregnancy (reported perceived distress (stress or depressive symptoms) using the 10-item PSS),[62] maternal antibiotic during pregnancy and prenatal smoking (maternal and second-hand). These variables were derived from questionnaires during pregnancy at 18 weeks.

Birth. Relevant exposures during delivery collected by the CHILD study included the mode of delivery (vaginal versus c-section), the season of birth (spring, summer, fall and winter), intrapartum antibiotic exposure, gestational age and gestational age-adjusted birth weight Z score,[63] derived from birth charts or questionnaires. Child sex was also collected from birth charts.

Postnatal. Postnatal exposures in this study included any breastfeeding duration, exclusive breastfeeding status at 6 months, regular childcare attendance at 3 months and 1 year (indicated by regularly going to a location at least 1 h per day on average or at least 7 h total in a week), the number of older siblings, child antibiotic use in the first year of life, cat and dog ownership and postnatal smoking (maternal and second-hand). These variables were derived from postnatal standardized questionnaires. In analyses of infant gut microbiota, child antibiotic use was defined as antibiotic exposure before the stool sample collection age and breastfeeding was defined as children were still breastfed at the stool sample collection age.

Home environment. Home environmental factors included home type (multiple-family or single-family), and the medium or high mold, carpet floor, and having potted plant, which were summarized from kitchen, bathroom, kid room, mother room and living room, clutter home (cluttered furniture or decoration generally at home), and frequency of use score of cleaning products. These variables were summarized from questionnaires and notes during the home assessment by the research assistant when the child was 3–4 months of age. The frequency of use score of cleaning products (FUS) was derived by summing the scores assigned to their questionnaire responses for the 26 categories of products in the previous CHILD study.[64]

Neighborhood environment. Physical Environmental factors included individual exposures to nitrogen dioxide (NO_2), dwelling density z−score, tree density and urbanicity (rural vs. urban). NO_2 is a measure of traffic-related air pollution, estimated from city-specific land use regression models[65] across pregnancy and in the first year of life. Dwelling density z-scores were calculated based on the number of dwellings per hectare using data from the CANUE urban environmental health datasets during pregnancy at 18 weeks. Urbanicity was determined by the rural/urban location at the home address where the family resided the longest around the time of birth (period covers pregnancy and first year of life) based on 2006 census data.[66] Tree density was defined using public tree census data. Tree locations were geocoded and averaged within a 250m buffer centered on the study participants' home addresses during pregnancy.

Parental diet and health. Parental diet factors included maternal ultra-processed food intake. Maternal diet was assessed using a validated quantitative food frequency questionnaire (FFQ) consisting of 151 food items. Food items were then classified into four groups according to the NOVA classification system: 1) unprocessed or minimally processed foods (MPF), 2) processed culinary ingredients (PCI), 3) processed foods (PF), and 4) ultra-processed foods (UPF). The dietary energy shares (%) contributed by UPF was calculated by dividing the energy intake from UPF by the total daily energy intake, then multiplied by 100 and was used to represent the maternal ultra-processed food intake.[67] Parental health factors included maternal and paternal asthma and atopy, maternal BMI, which were derived from questionnaires during pregnancy at 18 weeks or 1 year postnatal, and maternal distress (reported perceived distress) at 1 year postnatal.

Genetic ancestry. Genetic ancestry was represented by the first 3 PC variables associated with self-reported parental ancestry derived from genotyped cord blood data using the principal component analysis.[68]

Stool sample collection

A subsample of 1,479 children had shotgun metagenomic data processed from fecal samples collected at 3 months (n = 1,422), 12 months(n = 1,426), or 3 and 12 months of age (n = 1,369) (Figure S1A). Sample collection and sequencing were performed as previously described.[22] Briefly, stool samples from diapers were collected at a home visit at around 3 months [mean (SD), 3.8 (1.1) months] and a clinic visit at around 1 year [mean (SD), 12.4 (1.3) months]. Samples were briefly stored at 4°C and then aliquoted into four 2-mL cryovials using a stainless steel depyrogenated spatula and were frozen at −80°C. CHILD recorded the time between stool collection and long-term storage. This processing time and the age of children at the time of stool sample collection were

adjusted for in our statistical analysis. Samples collected for children over 1.5 years of age or samples with processing time higher than 100 h were excluded.

$COPSAC_{2010}$ cohort

Characteristics of the study population

Similar to the CHILD study, the $COPSAC_{2010}$ cohort is an independent population-based mother-child cohort of 700 children and their families in Denmark. Families were enrolled in pregnancy and children were followed prospectively by $COPSAC_{2010}$ study physicians. All biosamples were collected by nurses and clinical diagnoses were assessed during clinical visits at 1 week, 1, 3, 6, 12, 18, 24, 30, and 36 months, yearly until the age of 6 and again at age 8 and 10 years. Information on duration of exclusive and total breastfeeding period was recorded during the scheduled visits to the research clinic.[69,70]

Socioeconomic status

The $COPSAC_{2010}$ cohort collected three measures of socioeconomic at 1 week after birth, including household income covering last 3 months of pregnancy (4 ordinal levels in Danish Krone: 1 = 0–150,000, 2 = 150,000–200,000, 3 = 200,000–250,000, 4 = over 250,000) and the highest education of mother and father mother (4 ordinal levels: 1 = complete high school or college, 2 = complete tradesman, 3 = complete medium academic, 4 = complete university).

Early risk factors for multiple NCDs

In the $COPSAC_{2010}$ cohort, information on physician-diagnosed asthma, duration of exclusive and total breastfeeding period was obtained during the scheduled visits to the research clinic. The blood sample and Strengths and Difficulties Questionnaire (SDQ) was assessed at 6-year clinic visit.

Childhood overweight/obese. Weight was measured by calibrated digital weight scales and without clothes. Length was measured until the age of 2 years using an infantometer (Kiddimeter; Raven Equipment Ltd, Dunmow, Essex, England). After the age of 2 years, height was measured using a stadiometer (Harpenden, Holtain Ltd, Crymych, Dyfed, Wales) which was calibrated yearly. BMI was calculated as weight (kg)/length^2 (m^2) and converted into age-and-sex-standardized z-scores using the R package ‘zscorer’.[71] Same as CHILD cohort, children were classified as underweight with BMI z-scores less than -1, normal from -1 to 1, overweight from 1 to 2, and obese over 2 at age 5 years.[60]

Childhood asthma. Asthma was diagnosed on the basis of a previously detailed quantitative symptom algorithm requiring all of the following criteria[72,73]: (i) verified diary recordings of five episodes of troublesome lung symptoms within 6 months, each lasting at least three consecutive days; (ii) symptoms typical of asthma including exercise-induced symptoms, prolonged nocturnal cough, and persistent cough outside of common colds; (iii) need for intermittent rescue use of inhaled β2-agonist; and (iv) response to a 3-month course of inhaled corticosteroids and relapse upon ending treatment.[72] Remission of asthma was defined by 12 months without relapse upon cessation of inhaled corticosteroid treatment. For analyses, we used the ever diagnosed asthma at age 5 years.

Childhood atopic sensitization. Specific IgE levels were determined at ages 0.5, 1.5, and 6 years by using a screening method (ImmunoCAP, Phadiatop Infant, ThermoFisher Scientific).[69] The 6-month blood sample was further analyzed using ImmunoCAP ISAC measuring 112 components from 51 different allergen sources. Levels $\geq$0.3 ISAC Standardized Units(ISU) were considered indicative of allergic sensitization. The 6-year blood sample was analyzed for dog, cat, grass, birch, mugwort, D pteronyssinus, molds, egg, milk, wheat flour, and peanut by ImmunoCAP, using any level $\geq$0.35kUA/L as indicative of sensitization, that is elevated specific IgE.[74]

Childhood emotional/behavioral problems. Childhood emotional/behavioral problems is defined based on SDQ at 6 years of age, which is a widely used screener for detecting mental health difficulties.[75] Higher scores indicate greater symptoms. To be comparable to externalizing (Conduct and Hyperactivity) or internalizing (Emotional and Peer problems) problems based on CBCL assessment in CHILD cohort. Children with score higher than clinical cut-off scores for the subscales (out of a possible 10)[75]: Emotion $\geq$5, Conduct $\geq$4, Hyperactivity $\geq$7, Peer Problems $\geq$4, were defined as children with emotional/behavioral problems.

Stool sample collection

Fecal samples were collected either at the research clinic or by the parents at home using detailed instructions. Each sample arrived at the laboratory was mixed on arrival with 1 mL of 10% (v/v) glycerol broth (SSI, Copenhagen, Denmark) and frozen at -80°C. DNA was extracted using the PowerMag Soil DNA Isolation Kit (Qiagen) and NucleoSpin Stool Kit (Macherey-Nagel). No differences were found between the different DNA extraction kits after comparison. Before library preparation, the DNA was quantified by Tecan Infinite F Nano+ Plate Reader using Quant-iT dsDNA BR Assay Kit. The enzymatic fragmentation of DNA and library construction was conducted by Tecan DreamPrep NGS using Celero EZ DNA-seq Core Module Kit. The fragmented DNA was amplified using polymerase chain reaction (PCR). Short and large DNA fragments were removed using double-sided magnetic bead size selection (AMPure XP, Beckman Coulter). Adapter sequences from Celero 96-Plex Adaptor Plate were added to each sample during library construction. The final concentration for each library was quantified by Tecan Infinite F Nano+ Plate Reader using NuQuant NGS Library Quantification Module and Qubit. The final fragment distribution is evaluated using a Fragment Analyzer 5200 (Agilent). Qubit and TapeStation were used to determine the concentration of the final library before sequencing. The library was sequenced using 2×150 bp paired-end sequencing on an Illumina NovaSeq 6000 platform (Illumina, San Diego, CA, USA). The shipment time and the age of children at the stool sample collection time were adjusted for in our statistical analysis. Similar to the CHILD study, samples collected from children over 1.5 years of age or those with shipment times exceeding 100 h were excluded.

Ethics and data access

The accession number for the shotgun metagenomic data reported in this paper is BioProject accession (NCBI): PRJNA838575. The informed consent obtained from the CHILD participants, in addition to the CHILD Inter-Institutional Agreement (IIA) which has been executed between the five Canadian institutions responsible for the study, govern the sharing of CHILD data. Data described in the manuscript are available by registration to the CHILD database (https://childstudy.ca/childdb/) and the submission of a formal request. All reasonable requests will be accommodated. More information about data access for the CHILD Cohort Study can be found at https://childstudy.ca/for-researchers/data-access/. Researchers interested in collaborating on a project and accessing CHILD Cohort Study data should contact child@mcmaster.ca. COPSAC sequencing data is available in the Sequence Read Archive (SRA) under accession no. PRJNA715601. Individual-level data is protected under Danish and European law that prohibits publication even in pseudonymized form. However, data can be made available to researchers under a data processing agreement by contacting COPSAC's Data Protection Officer (administration@dbac.dk).

Sex and gender considerations

In this study, biological sex assigned at birth was included as a covariate in all models examining associations between socioeconomic status, perinatal factors and childhood non-communicable diseases risk factors. In CHILD cohort, 52.6% children were male ($n = 3{,}263$) and male sex was positively associated with atopy, asthma and overweight/obesity at 5 years of age (all p-value<0.05), but was not significantly associated with emotional/behavioral problems at 5 years of age. In COPSAC cohort, 51.4% were male ($n = 700$) and sex was not significantly associated with childhood atopy, asthma, overweight/obesity, and emotional/behavioral problems. These findings indicate cohort specific sex difference in childhood health outcomes, while supporting the inclusion of sex as an important adjustment factor in analyses. Given the young age of participants, self-identified gender was largely concordant with sex assigned at birth, therefore gender related analyses were not feasible in this study.

METHOD DETAILS

In CHILD cohort, shotgun metagenomic sequencing data with an average depth of 5 million reads per sample was generated by Diversigen (Minneapolis, MN, USA) from fecal samples. DNA was extracted from fecal samples using the MO Bio PowerSoil Pro with bead beating in 0.1mm glass bead plates. High-quality input DNA was verified using Quant-iT Picogreen. Libraries were then prepared following a procedure adapted from the Illumina Nextera Library Prep kit and sequenced on an Illumina NextSeq using single-end 1×150 reads. Low quality (Q-Score<30) and length (<50) sequences were removed, and adapter sequences trimmed. Host and low-quality reads were removed, and only samples with at least 100,000 remaining reads or more were retained for downstream analysis. Shotgun metagenomic reads were mapped using the bioBakery 3 pipeline[53] to identify taxonomic (species and strain level) and functional features within each sample. To generate the abundance of Bifidobacterium longum subspecies, MetaPhlAn database was customized with a set of maker genes including 119 *Bifidobacterium logum. subspecies infantis* and 128 *Bifidobacterium longum subspecies longum* markers using MetaPhlAn instructions (https://github.com/biobakery/MetaPhlAn/wiki/MetaPhlAn-4) and then MetaPhlAn4 was used with the –index and –bowtie2db parameters and the customized marker-gene database[76] and HUMAnN 3 was used for functional profiling.

In COPSAC cohort, quality control of raw FASTQ files was performed using KneadData (v. 0.6.1) to remove low-quality bases and reads derived from the host genome as follows: Using Trimmomatic (v. 0.36), the reads were quality trimmed by removing Nextera adapters, leading and trailing bases with a Phred score below 20, and trailing bases in which the Phred score over a window of size 4 drops below 20. Trimmed reads shorter than 100 bases were discarded as low-quality reads. Reads that mapped to the human reference genome GRCh38 (with Bowtie2 v. 0.2.3.2 using default settings)[77] were also discarded. Read pairs in which both reads passed filtering were retained; these were classified as high-quality non-host (HQNH) reads. Shotgun metagenomic reads were mapped using the bioBakery 3 pipeline[53] to identify taxonomic (species and strain level) and functional features within each sample. Similar to the CHILD cohort, the MetaPhlAn database was customized to generate the abundance of *Bifidobacterium longum subspecies*.[76] The average sequencing depth was 24 million paired-end reads per sample and only samples with at least 100,000 remaining reads or more were retained for downstream analysis.

QUANTIFICATION AND STATISTICAL ANALYSIS

Data analysis was conducted in R (version 4.3.1). We summarized the SES latent factor using confirmatory factor analysis (CFA) using SES observed factors, including household income, the highest education of mother and father, and the MacArthur scale of subjective social status in Canada, and the community collected during pregnancy at 18 weeks (Figure 1A). The COPSAC_{2010} SES latent factor was generated using SES observed factors, including household income, the highest education of mother and father (Figure 4A). Models were estimated using "cfa" function included in the "lavaan" R package.[55] Household income and the highest education of mother and father were treated as ordered categorical variables in the models. Models were stepwise updated using the function "modindices"[55] which identified significant correlations between two covariant variables (chi-square p-value <0.05) to improve the model fit. "lavPredict" function was used to compute the estimated values ('factor scores') for the SES latent variable in the model, which was used to represent summarized SES index for later analysis. Item cluster analysis was performed to confirm the associations across the observed SES factors using "iclust" function from "psych" R package[56] to cluster SES factors and

perinatal factors and exposomes using correlations with pairwise deletion of samples (Figure S1B). Linear regression models with adjustment of study center and sex were applied to explore the associations between SES, childhood NCD risk factors and each perinatal factor. The confidence interval was estimated using the "confint" function from the "stats" R package. Factors with p-values <0.05 were defined as significant. p-values were without adjustment of multiple comparisons, while q-values (FDR) represent p-values with adjustment of multiple comparisons using Benjamini-Hochberg (BH) method (Figure 1). Ethnicity or genetic ancestry is well-known to be associated with SES. To fully understand the effects of SES and explore the robustness of the summarized SES index, we performed sensitivity analyses with and without adjustment of the genetic ancestry using the first 3 genetic ancestry PCs. In addition, sensitivity analyses using 1-year summarized SES index, each study center, and each of the observed 5 SES factors were also performed, in which the ordinal SES categorical variables were treated as numeric variables based on their orders (Figure S1C).

Microbial community structure was measured by using the within-individual species diversity (α-diversity) by Shannon index on the relative abundance of species (Figure 2A). To test the associations between infant gut microbiota and SES index and perinatal factors, Aitchison's distance ("vegan") was calculated on the modified centered log-ratio (mCLR)-transformed[57] relative abundance of species. mCLR transform applies central log transformation to non-zero values and adds a pseudocount of the minimal value to them, and the previous exact zero relative abundance entries in the OTU table remained zero using "mclr" function from "metaMint" package.[57] Then, a marginal Permutational Multivariate Analysis of Variance (PERMANOVA) was performed using "adonis2" function from "vegan" R package across 1000 permutations with adjustment of stool sample collection age, processing time and a strata of study center[58] (Figure 2C). The beta diversity based on the Aitchison's metric was used to perform principal coordinates analysis on mCLR-transformed relative abundance of species. The association between the alpha diversity and the first three principal coordinates (PCoAs) with SES index were tested using Wilcoxon tests (compare children with the second, third, and fourth quartiles of familial SES index to children with the first quartile of SES index) and linear mixed effect models (test association with continuous SES index) with study center as a random effect and adjustment of stool sample collection age and processing time. Statistical significance was defined as a p-value <0.05 (Figures 2A and 2B). The top and/or SES-associated PCoAs were graphed to visualize the sample group relationships, where the arrows represent the strength and direction of the top 10 factors that explain microbiome variation using Pearson correlation. Ellipses represent 95% confidence ellipses for each SES category using a multivariate t-distribution, with the shaded areas illustrating the distribution of samples within each SES group (Figure 2D). As we hypothesized that the effect of SES on overall infant gut microbiota would be through perinatal factors, structural equation modeling (SEM), a statistical technique used to evaluate how well observed data match hypothesized causal relationships, was then used to assess the potential mediations using the R package "lavaan".[55] For model specification, we simultaneously estimated the mediation effects (indirect effects) of perinatal factors on SES-associated infant gut microbiota composition using PCoA at 3 months and 1 year, respectively (PCoA1 for 3-month and PCoA3 for 1-year infant microbiota) (Figure 2E). Using $COPSAC_{2010}$ cohort, the mediation effect of breastfeeding was estimated on SES-associated infant gut microbiota composition using PCoA3 at 1 month and 1 year, respectively (Figure 4). All regressions in SEM models were adjusted for study center and regressions with microbiome data were also adjusted for stool sample collection age and processing/shipment time. In regression models and SEM models, all categorical variables were replaced by dummy variables, i.e., study center was replaced by Vancouver, Toronto and Edmonton. All univariable and SEM models, which focused on individual perinatal factors, excluded observations without pairwise data (considered missing at random). To explore differences in perinatal factors' effect on infant gut microbiota between children with and without breastfeeding, we compared the beta dispersion (Figure 3A) and repeated our PERMANOVA analysis on samples stratified by breastfeeding status (Figure 3B). The breastfeeding (BF) group included children who were still breastfed at the stool sample collection age and no breastfeeding (No BF) group included children who were either never or not breastfed at the 3 months and stool sample collection age, which captures children with a breastfeeding duration of less than 3 months and ensures that the gut microbiome reflects a state without active breastfeeding at the time of sampling. To account for differences in sample size, a bootstrapping test (randomly selecting the same number of samples as the smaller group with replacement for 100 runs) was applied. Factors with p-value based on PERMANOVA analysis less than 0.05 for at least 80% of runs were considered significant. Moreover, to explore the effect of breastfeeding on childhood NCD risk factors for children with different familial SES, a compound variable was generated based on the lower or upper half of the SES index and exclusive breastfeeding status to 6 months. Then, logistic regression with adjustment of study center and sex was applied to examine its association with the risk of at least one SES-associated childhood risk factors for NCDs (childhood overweight/obesity, asthma and emotional/behavioral problems at 5 years of age) with children from lower half SES index family and not breastfed to 6 months as a reference group (Figure 3C). Statistical significance was defined as a p-value <0.05.

The "Phyloseq" package[52] was used to preprocess the metagenomic taxonomy table. To identify bacterial species that were significantly associated with SES, perinatal factors, and childhood NCD risk factors, linear mixed-effect models (MaAsLin2) models were applied to the mCLR transformed relative abundance of each species and Metacyc pathways (Figures 5, S4, and S5). Only species and Metacyc pathways detected in at least 10% of samples were used. Longitudinal models were performed with and without interaction between time and factor, with the study center and subject ID as random effects. For each species and factor, the formula of model with interaction is Species (mCLR-transformed relative abundance) $\sim \beta_{a1}$*factor + β_{a2}*stool sample collection age + β_{a3}*SES*stool sample collection age+ β_{a4}*processing time/shipment time +1|(site+subject)), in which β_{a3} is for the slope effect, measuring the change of factor over time, while β_{a1} is for the baseline effect, measuring the baseline difference. The formula of the model without interaction is Species $\sim \beta_{b1}$*factor + β_{b2}*stool sample collection age+ β_{b3}*processing time/shipment

time +1|(site+subject), in which β_{b1} measures the effect of the factor on overall colonization of the species. All MaAsLin2 models were adjusted for stool sample of collection age and processing time/shipment time and the interaction term in MaAsLin2 model was tested by multiplying the two relevant variables (SES x stool sample collection age). Benjamini-Hochberg procedure was used to correct *p*-values, and tests with MaAslin2 qval (FDR) < 0.1 were considered to be significant. Then, to test the mediation effect of infant microbiome on SES-associated child health outcomes, again SEM model was applied, in which we conceptualized the infant gut microbiome (i.e., latent variable) using CFA, including the 30 SES-associated species at 3 months and 1 year (Figure 6). “modindices” was used to identify significant (chi-square *p*-value <0.05) correlations across species to improve the model fit. All regressions in SEM models were adjusted for study center and sex, and regressions with microbiome data were also adjusted for stool sample collection age and processing time/shipment time. Models with CFI exceeding 0.9, RMSEA lower than 0.05, and SRMR lower than 0.08 are considered a good fit.[78]

Cell Reports Medicine, Volume *7*

Supplemental information

Breastfeeding may lessen socioeconomic disparities in child health through differences in the infant gut microbiome

Darlene L.Y. Dai, Melissa B. Manus, Courtney Hoskinson, Jie Jiang, Hind Sbihi, Kozeta Miliku, Susan C. Campisi, Daphne J. Korczak, Qingling Duan, Theo J. Moraes, Piushkumar J. Mandhane, B. Brett Finlay, Elinor Simons, Hannah Lishman, David M. Patrick, Padmaja Subbarao, Meghan B. Azad, Bo Chawes, Klaus Bønnelykke, Søren Johannes Sørensen, Jonathan Thorsen, Jakob Stokholm, Charisse Petersen, and Stuart E. Turvey

Supplemental Figures and Tables

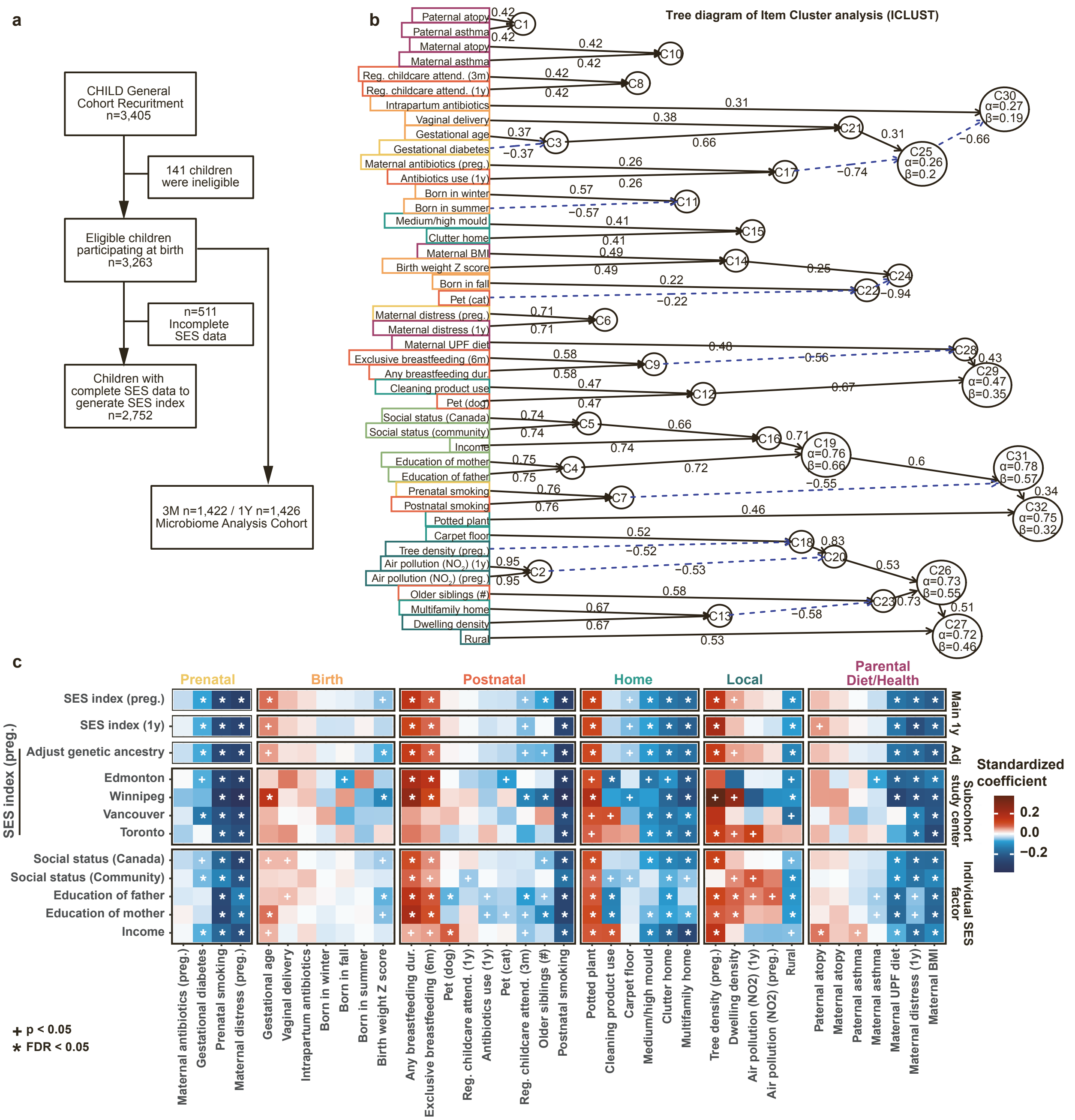

Fig. S1. Associations across SES, perinatal factors, and childhood health outcomes, Related to Figure 1. (a) Consort diagram of CHILD Cohort. (b) Tree diagram of the item cluster analysis (ICLUST) to cluster observed SES factors and perinatal factors and exposomes based on their Pearson correlations. Cluster 19 (C19) was found to include the 5 observed SES factors with Cronbach's alpha values >0.7, indicating internal consistency within the calculated clusters. (c) Heatmap of the associations between summarized SES index at enrollment and perinatal factors, and sensitivity analysis using summarized SES index based on observed SES factors collected at 1 year, with adjustment of genetic ancestry, sub-cohort analyses for each study center and individual observed SES factors using regression models. Red represents positive association and blue represents negative associations with + indicating p<0.05 and * indicating FDR<0.05. Factors are ordered by hierarchical clustering.

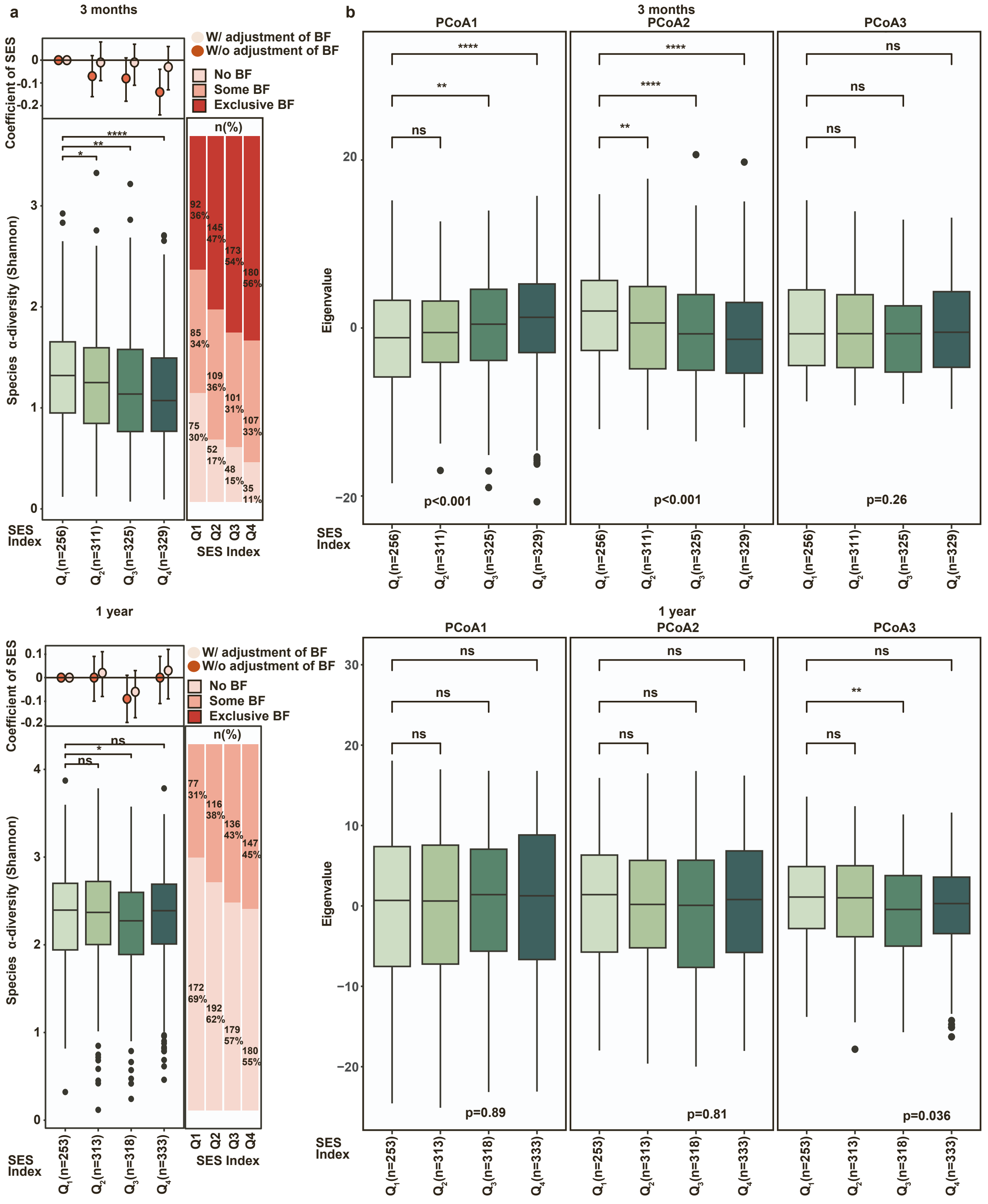

Fig. S2. SES is associated with infant microbiome composition at 3 month and 1 year, Related to Figure 2. (a) Differences in gut microbiota species α-diversity (Shannon index) at 3 months and 1 year across children with different quartiles of SES index. SES Q1 includes samples with the lowest 25% SES index and SES Q4 includes samples with the top 25% SES index. The right-side stacked bar plots showed the proportion of some (dark pink) and exclusive (orange) breastfeeding across SES quartiles. The top panels show the associations between species α-diversity and SES quartiles using linear regression models with (pink) and without (orange) adjustment of breastfeeding. Linear

regression models were all adjusted for stool sample collection age and processing time and with study site as a random effect. (b) Boxplot of the top 3 PCoAs across SES quartiles. Differences across SES quartiles compared to the first quartile of SES were estimated based on Wilcoxon test. ns $p>0.05$, * $p<0.05$, ** $p<0.01$, and *** $p<0.001$. The box displays 25th, 50th (median), and 75th percentiles, with whiskers extending 1.5 * IQR. P-values at the bottom of each figure were based on linear regression models using the continuous SES index with study site as a random effect and adjustment of stool sample collection age and processing time.

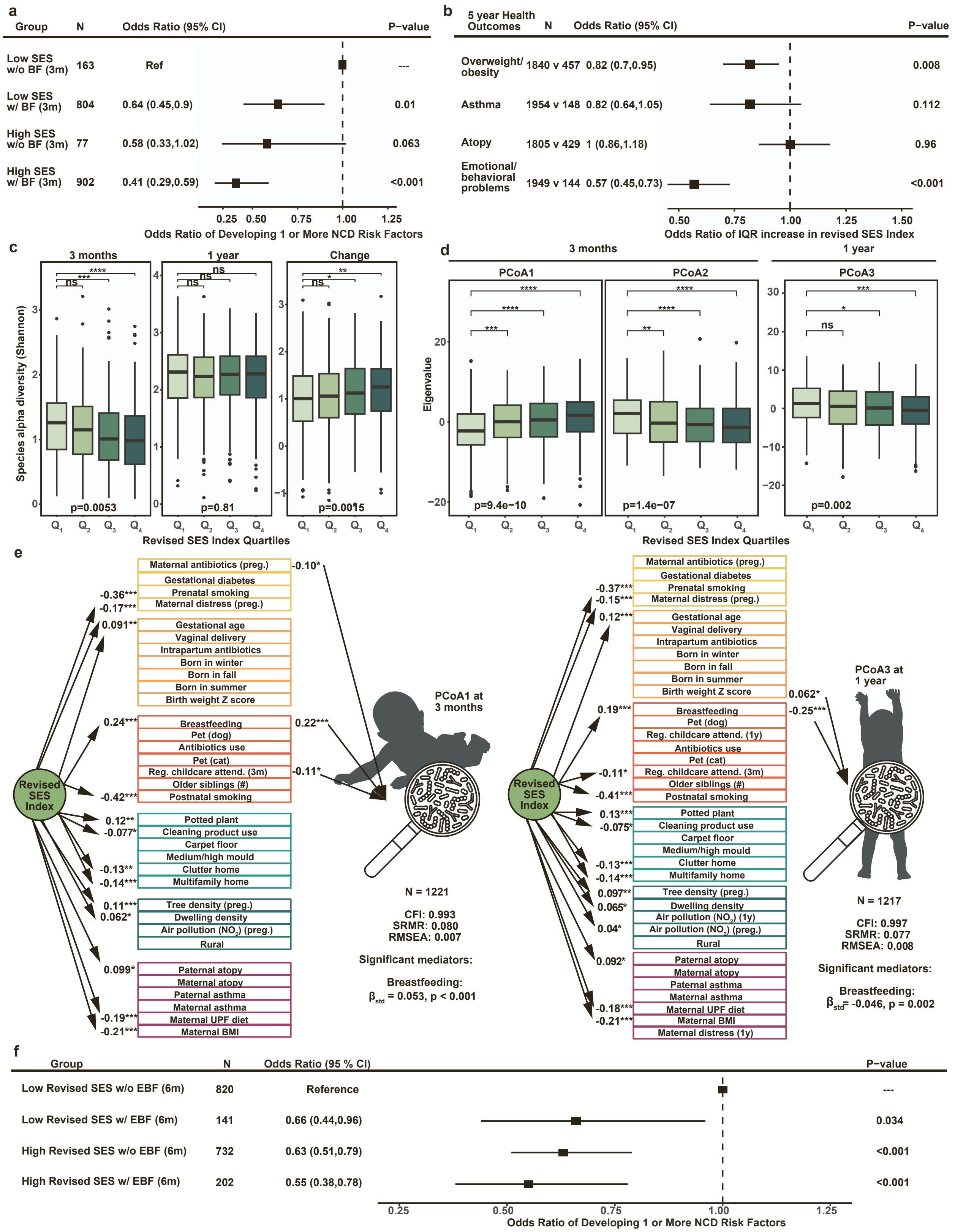

Fig. S3. Sensitivity analyses in CHILD Cohort examining any breastfeeding up to 3 months and the revised SES index, Related to Figure 2 and 3. (a) Forest plot of odds ratio (95% CI) for compound SES and any breastfeeding status (BF) at 3 months on the presence of one or more SES-associated NCD risk factor at age 5 (childhood

overweight/obesity, asthma and emotional/behavioral problems). (b) Forest plot of odds ratio (95% CI) for the association between childhood NCD risk factors at 5 years of age and an interquartile increase in the revised SES index, which is derived using only the three indicators applied in COPSAC (income and highest parental education). (c) Gut microbiota α-diversity (Shannon index) at 3 months, 1 year, and the change between these ages, and the top three significant PCoA axes (d) across the revised SES quartiles (Q_1 = lowest 25%, Q_4 = highest 25 %). Wilcoxon tests compare each quartile to Q_1. P-values shown beneath each panel are from regression models using the continuous revised SES index with study site as a random effect and adjustment for stool collection age and processing time. ns $p>0.05$, * $p<0.05$, ** $p<0.01$, *** $p<0.001$. (e) SEM models linking the revised SES index, perinatal factors and exposomes, and infant gut microbiota (PCoA1 at 3 months on left; PCoA3 at 1 year on right). Only significant indirect effects and associations (one-headed arrows; $p<0.05$) are shown. (f) Forest plot of odds ratio (95% CI) for the revised SES index and exclusive breastfeeding status (EBF) at 6 months on the presence of one or more SES-associated NCD risk factor at age 5 (childhood overweight/obesity, asthma and emotional/behavioral problems).

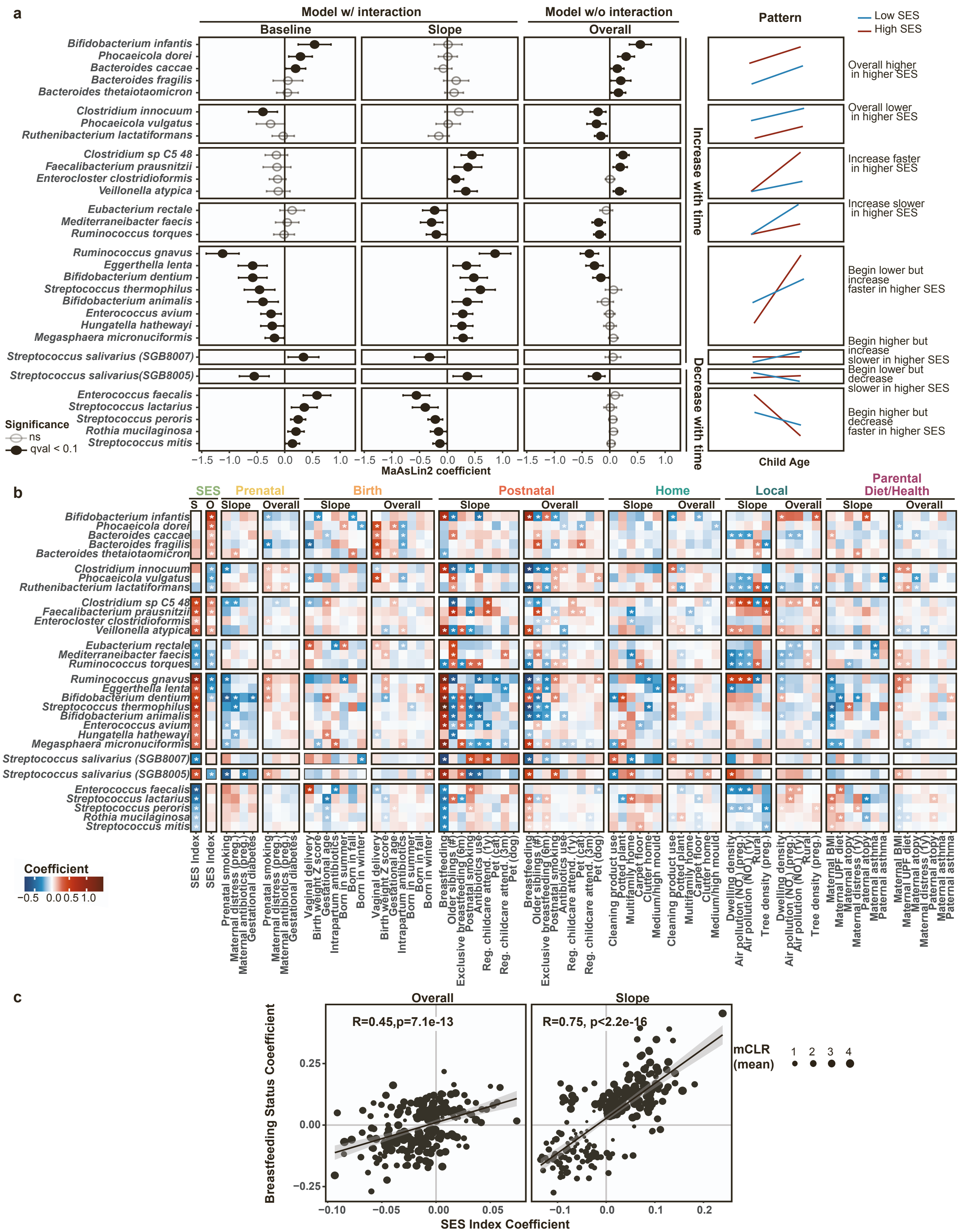

Fig. S4. **Species and Metacyc pathways associated with SES, breastfeeding, and perinatal factors and exposomes, Related to Figure 5.** (a) Baseline and slope effect (model with interaction) and overall effect (model without interaction) of SES index for species significantly associated with SES (FDR of slope effect or overall effect

< 0.1) across 3 months to 1 year based on MaAslin2 models. Data were presented as standardized coefficients of SES ± 1.96*standard error of the mean. The right-side panel summarizes the pattern of SES and time effect on each species across child age, in which the red line represents higher SES and the blue line represents lower SES and there is an interaction if the slope effect is significant. (b) Heatmap of the standardized slope and overall effect of perinatal factors on SES-associated species based on MaAslin2 models (FDR <0.1). Red represents positive association, while blue represents negative association. Stars represent significant associations (FDR < 0.1). (c) Correlation plot of overall effect (MaAslin2 models without interaction measuring overall colonization) and slope effect (MaAslin2 models with interaction measuring change over time) between SES and breastfeeding for Metacyc pathways significantly associated with either SES or breastfeeding (FDR of slope or overall effect < 0.1). Dot size represents the mean of relative abundance of Metacyc pathways across 3 months and 1 year. All MaAslin2 models used subject ID and study center as random effects and were adjusted for stool sample collection age and processing time.

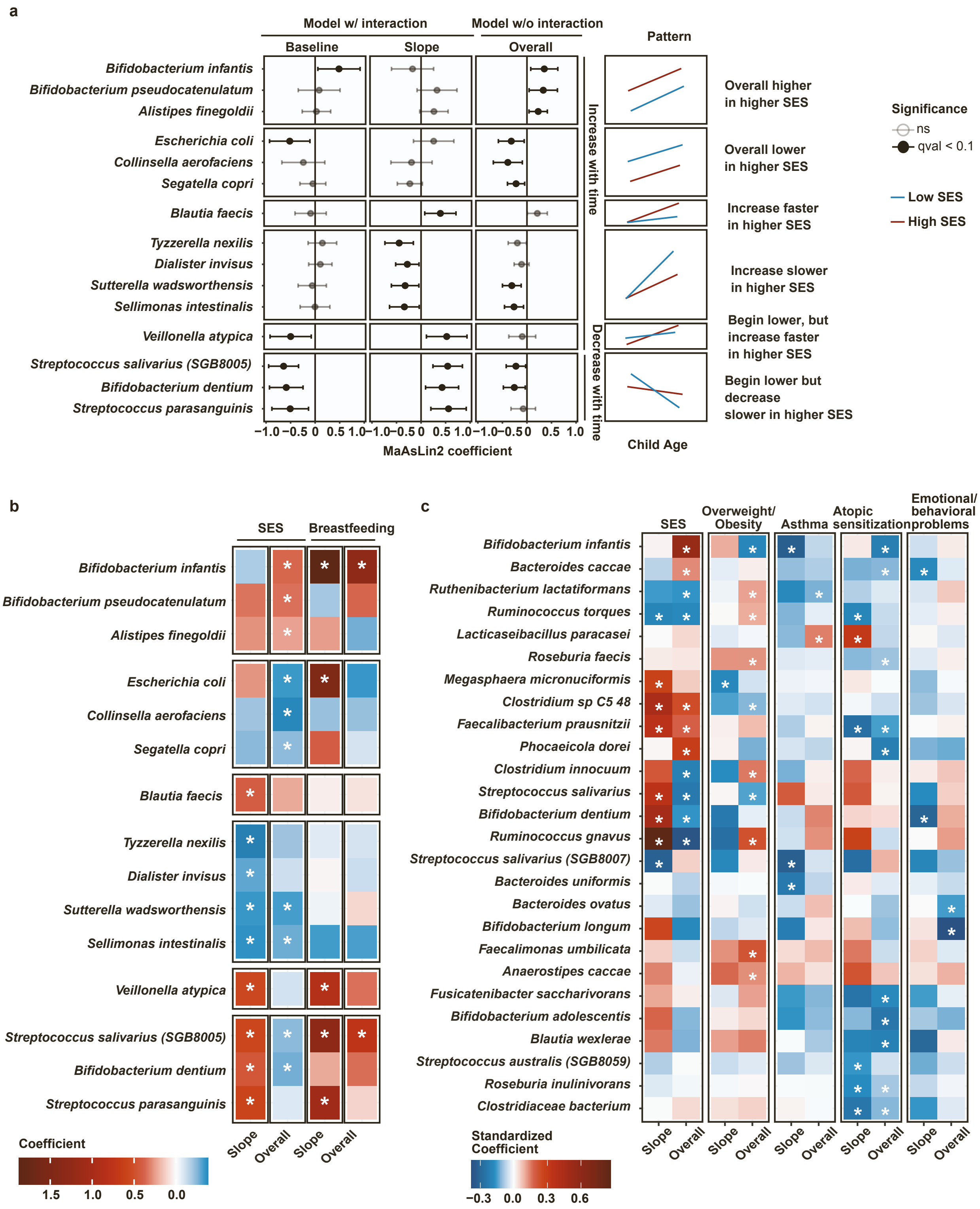

Fig. S5. Species significantly associated with SES and breastfeeding in COPSAC$_{2010}$ cohort, Related to Figure 4 and 6. (a) Baseline and slope effect (model with interaction) and overall effect (model without interaction) of SES index for species significantly associated with COPSAC$_{2010}$ SES index (FDR of slope effect or overall effect < 0.1)

across 1 month to 1 year based on MaAslin2 models. Data were presented as standardized coefficients of SES ± 1.96*standard error of the mean. The right-side panel summarizes the pattern of SES and time effect on each species across child age, in which the red line represents higher SES and the blue line represents lower SES and there is an interaction if the slope effect is significant. (b) Heatmap of the standardized slope and overall effect of SES and breastfeeding on SES-associated species based on MaAslin2 models (FDR <0.1). Red represents positive association, while blue represents negative association. Stars represent significant associations (FDR < 0.1). (c) Heatmap of standardized coefficient of SES index and childhood NCD risk factors for species significantly associated with at least one of four childhood NCD risk factors (FDR < 0.1) using MaAslin2 models with (slope effect) and without (overall effect) interaction in CHILD Cohort. All MaAslin2 models used subject ID and study center as random effects and were adjusted for stool sample collection age and processing time. Red represents positive association, while blue represents negative association. Stars represent FDR < 0.1.

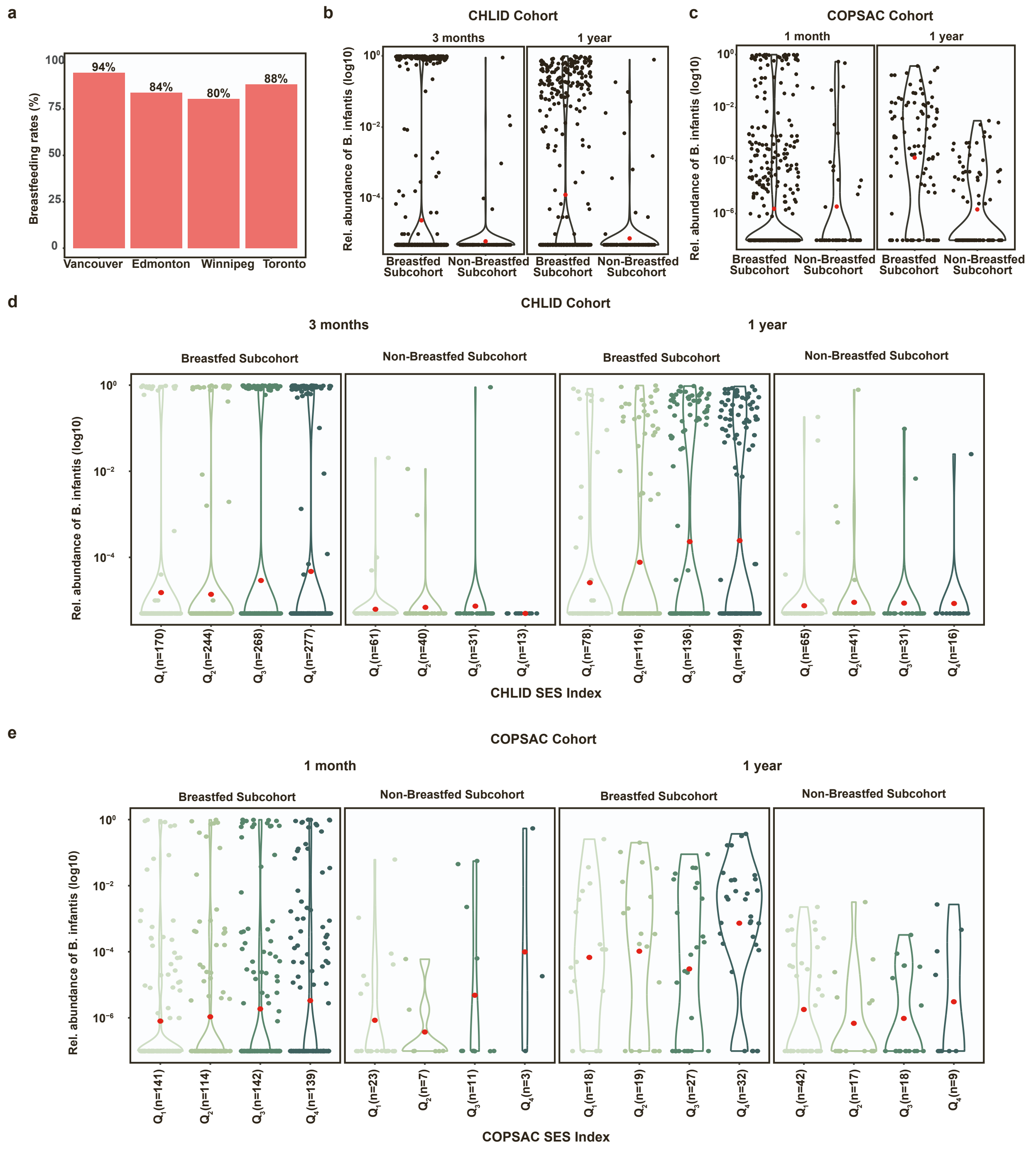

Fig S6. Breastfeeding rate in CHILD cohort and *B. infantis* abundance in COPSAC2010 cohort, Related to Figure 6. (a) Breastfeeding rate (some breastfeeding at least 3 months) across CHILD study sites. (b-c) Violin plot of log 10 transformed relative abundance of *B. infantis* in Breastfed subcohort children and Non-Breastfed subcohort children in CHILD cohort (b) and COPSAC2010 cohort (c). (d-e) Violin plot of log 10 transformed relative abundance of *B. infantis* in Breastfed and Non-Breastfed subcohort children at each visit in CHILD cohort (3-month visit and 1-year visit) (d) and COPSAC2010 cohort (1-month visit and 1-year visit) (e). Red dot represents the mean.

Table S1. Characterizations of CHILD samples and across SES quartiles, Related to STAR Methods.

Category	Variable	Cohort	SES Index Q_1 (0-25%)	SES Index Q_2 (25-50%)	SES Index Q_3 (50-75%)	SES Index Q_4 (75-100%)
No. patients		3263	691	690	684	687
Confounder	Male, n(%)					
		1717 (52.6%)	350 (50.7%)	361 (52.3%)	374 (54.7%)	368 (53.6%)
	Study center, n(%)					
	Edmonton	768 (23.5%)	181 (26.2%)	206 (29.9%)	169 (24.7%)	104 (15.1%)
	Toronto	768 (23.5%)	71 (10.3%)	119 (17.2%)	162 (23.7%)	284 (41.3%)
	Vancouver	735 (22.5%)	109 (15.8%)	150 (21.7%)	199 (29.1%)	212 (30.9%)
	Winnipeg	992 (30.4%)	330 (47.8%)	215 (31.2%)	154 (22.5%)	87 (12.7%)
Health	Atopic sensitization at 5y, n(%)					
		494 (19.3%)	82 (15.2%)	107 (18.9%)	119 (21.4%)	113 (21.4%)
	Unknown	708 (21.7%)	151 (21.9%)	125 (18.1%)	128 (18.7%)	160 (23.3%)
	Asthma at 5y, n(%)					
		165 (6.9%)	37 (7.5%)	34 (6.5%)	44 (8.3%)	28 (5.4%)
	Unknown	864 (26.5%)	199 (28.8%)	167 (24.2%)	154 (22.5%)	171 (24.9%)
	Overweight/obese at 5y, n(%)					
		542 (20.6%)	125 (22.8%)	152 (26.2%)	92 (16.1%)	81 (14.7%)
	Unknown	638 (19.6%)	142 (20.5%)	109 (15.8%)	113 (16.5%)	137 (19.9%)
	Emotional/behavioral problems at 5y, n(%)					
		172 (7.3%)	49 (10.3%)	42 (7.8%)	24 (4.7%)	27 (5.1%)
	Unknown	903 (27.7%)	215 (31.1%)	153 (22.2%)	168 (24.6%)	159 (23.1%)
Pregnancy	Maternal antibiotics (preg.), n(%)					
		314 (9.6%)	81 (11.7%)	75 (10.9%)	61 (8.9%)	61 (8.9%)
	Gestational diabetes, n(%)					
		154 (4.7%)	52 (7.5%)	39 (5.7%)	19 (2.8%)	22 (3.2%)
	Maternal distress (preg.)					
	Median (Range)	13 (0, 35)	16 (0, 35)	13 (0, 33)	11 (0, 30)	10.5 (0, 34)
	IQR (Q1,Q3)	9, 17	11, 20	9, 17	7, 16	7, 14.8
	Unknown	325 (10%)	57 (8.2%)	48 (7%)	53 (7.7%)	33 (4.8%)
	Prenatal smoking, n(%)					
		606 (19.1%)	242 (35.3%)	125 (18.2%)	84 (12.3%)	50 (7.3%)
	Unknown	97 (3%)	5 (0.7%)	2 (0.3%)	2 (0.3%)	2 (0.3%)
Birth	Intrapartum antibiotics, n(%)					
		1326 (41.2%)	124 (24.1%)	135 (27%)	143 (28.2%)	137 (26.9%)
	Unknown	45 (1.4%)	176 (25.5%)	190 (27.5%)	177 (25.9%)	178 (25.9%)
	Delivery mode, n(%)					
	Vaginal	2412 (74.8%)	515 (76%)	502 (73.5%)	508 (74.9%)	511 (75.3%)
	C-section with labor	425 (13.2%)	88 (13%)	101 (14.8%)	93 (13.7%)	80 (11.8%)
	C-section without labor	387 (12%)	75 (11.1%)	80 (11.7%)	77 (11.4%)	88 (13%)
	Unknown	39 (1.2%)	13 (1.9%)	7 (1%)	6 (0.9%)	8 (1.2%)
	Birth weight Z score					
	Median (Range)	-0.1 (-3.1, 4.3)	0 (-2.9, 3.9)	-0.1 (-2.5, 4.3)	-0.2 (-2.6, 3.3)	-0.1 (-3.1, 2.8)
	IQR (Q1,Q3)	-0.7, 0.5	-0.6, 0.6	-0.7, 0.6	-0.7, 0.5	-0.7, 0.4
	Unknown	75 (2.3%)	20 (2.9%)	17 (2.5%)	13 (1.9%)	17 (2.5%)
	Gestational age					
	Median (Range)	278 (238, 300)	277 (240, 299)	278 (240, 300)	278 (241, 296)	278 (240, 295)
	IQR (Q1,Q3)	272, 283	271, 282	272, 283	272, 284	272, 284
	Unknown	54 (1.7%)	16 (2.3%)	10 (1.4%)	8 (1.2%)	12 (1.7%)
	Season of birth, n(%)					
	Spring	889 (27.2%)	181 (26.2%)	173 (25.1%)	202 (29.5%)	204 (29.7%)
	Summer	830 (25.4%)	168 (24.3%)	204 (29.6%)	148 (21.6%)	175 (25.5%)
	Fall	754 (23.1%)	167 (24.2%)	166 (24.1%)	164 (24%)	150 (21.8%)
	Winter	790 (24.2%)	175 (25.3%)	147 (21.3%)	170 (24.9%)	158 (23%)
Postnatal	Antibiotics use (1y), n(%)					
		619 (26.1%)	137 (28.2%)	128 (24.5%)	128 (23.8%)	124 (24.3%)
	Unknown	890 (27.3%)	206 (29.8%)	168 (24.3%)	146 (21.3%)	176 (25.6%)
	Reg. childcare attend. (1y), n(%)					
		735 (28.5%)	150 (28.4%)	165 (28.4%)	175 (31.2%)	152 (27.3%)
	Unknown	688 (21.1%)	163 (23.6%)	110 (15.9%)	123 (18%)	131 (19.1%)
	Reg. childcare attend. (3m), n(%)					
		338 (11.3%)	85 (13.5%)	80 (12.5%)	73 (11.3%)	54 (8.4%)
	Unknown	274 (8.4%)	61 (8.8%)	48 (7%)	38 (5.6%)	43 (6.3%)
	Any breastfeeding dur.					
	Median (Range)	10 (0, 30)	8 (0, 25)	10 (0, 26)	11 (0, 24)	11 (0, 30)
	IQR (Q1,Q3)	5, 14	3, 13	5.5, 14	6, 15	6, 15
	Unknown	106 (3.2%)	21 (3%)	12 (1.7%)	15 (2.2%)	12 (1.7%)
	Pet (cat) (1y), n(%)					
		550 (21.4%)	136 (25.7%)	128 (22.2%)	113 (20.2%)	113 (20.4%)
	Unknown	690 (21.1%)	161 (23.3%)	113 (16.4%)	125 (18.3%)	134 (19.5%)
	Pet (dog) (1y), n(%)					
		701 (27.1%)	155 (29.2%)	163 (28.1%)	165 (29.4%)	123 (22.1%)
	Unknown	681 (20.9%)	160 (23.2%)	109 (15.8%)	123 (18%)	131 (19.1%)
	Older siblings (#)					
	Median (Range)	0 (0, 4)	1 (0, 4)	0 (0, 4)	0 (0, 3)	0 (0, 3)
	IQR (Q1,Q3)	0, 1	0, 1	0, 1	0, 1	0, 1
	Unknown	101 (3.1%)	6 (0.9%)	4 (0.6%)	2 (0.3%)	2 (0.3%)
	Postnatal smoking, n(%)					

		620 (23.1%)	241 (41.6%)	148 (25%)	80 (14.1%)	38 (6.7%)
	Unknown	578 (17.7%)	111 (16.1%)	98 (14.2%)	118 (17.3%)	122 (17.8%)
	Exclusive breastfeeding (6m), n(%)					
		522 (17.1%)	94 (14.5%)	94 (14.2%)	120 (18.5%)	151 (23.3%)
	Unknown	208 (6.4%)	41 (5.9%)	30 (4.3%)	36 (5.3%)	39 (5.7%)
Home Environment	Cleaning product use					
	Median (Range)	31 (4, 76)	32 (4, 76)	31 (8, 76)	31 (7, 66)	31 (9, 68)
	IQR (Q1,Q3)	25, 38	25, 40	24, 37	24, 38	25, 38
	Unknown	341 (10.5%)	79 (11.4%)	64 (9.3%)	56 (8.2%)	55 (8%)
	Multifamily home, n(%)					
		878 (28.9%)	200 (31.6%)	183 (28.1%)	184 (28.3%)	184 (27.9%)
	Unknown	223 (6.8%)	58 (8.4%)	38 (5.5%)	33 (4.8%)	28 (4.1%)
	Clutter home, n(%)					
		581 (19.1%)	186 (29.4%)	131 (20.1%)	86 (13.2%)	74 (11.3%)
	Unknown	229 (7%)	59 (8.5%)	38 (5.5%)	34 (5%)	30 (4.4%)
	Potted plant, n(%)					
		1822 (59.9%)	305 (48.2%)	388 (59.5%)	435 (66.8%)	451 (68.4%)
	Unknown	223 (6.8%)	58 (8.4%)	38 (5.5%)	33 (4.8%)	28 (4.1%)
	Carpet floor, n(%)					
		1722 (56.6%)	410 (64.8%)	377 (57.8%)	372 (57.1%)	315 (47.8%)
	Unknown	223 (6.8%)	58 (8.4%)	38 (5.5%)	33 (4.8%)	28 (4.1%)
	Medium/high mould, n(%)					
		359 (11.8%)	87 (13.7%)	73 (11.2%)	75 (11.5%)	68 (10.3%)
	Unknown	223 (6.8%)	58 (8.4%)	38 (5.5%)	33 (4.8%)	28 (4.1%)
Neighborhood Environment	Dwelling density					
	Median (Range)	0.2 (-0.8, 10.7)	0.1 (-0.8, 9.4)	0.1 (-0.8, 10.7)	0.3 (-0.8, 9.7)	0.9 (-0.7, 10)
	IQR (Q1,Q3)	-0.2, 1.4	-0.3, 0.6	-0.2, 0.9	-0.1, 1.6	0, 2.2
	Unknown	197 (6%)	22 (3.2%)	13 (1.9%)	14 (2%)	9 (1.3%)
	Air pollution (NO_2) (preg.)					
	Median (Range)	10.6 (1.3, 35.8)	7.3 (1.3, 30.8)	10.2 (1.3, 26.9)	11.1 (1.4, 33.7)	13 (1.4, 35.8)
	IQR (Q1,Q3)	5.3, 15	4, 12.5	5.1, 14.5	7, 15	9.8, 16.7
	Unknown	191 (5.9%)	40 (5.8%)	34 (4.9%)	40 (5.8%)	33 (4.8%)
	Air pollution (NO_2) (1y)					
	Median (Range)	9.1 (0.5, 30.5)	6.1 (0.5, 26.3)	8.8 (1, 23.1)	9.4 (1.4, 28.7)	11.5 (1.2, 30.5)
	IQR (Q1,Q3)	4.6, 13.3	3.7, 10.7	4.5, 12.3	5.8, 13.3	8.3, 15
	Unknown	194 (5.9%)	41 (5.9%)	36 (5.2%)	39 (5.7%)	35 (5.1%)
	Tree density (preg.)					
	Median (Range)	20 (0, 99)	17 (0, 91)	18 (0, 92)	20 (0, 94)	26 (0, 99)
	IQR (Q1,Q3)	13, 32	11, 23.5	10, 26	14, 32	16, 62
	Unknown	663 (20.3%)	192 (27.8%)	150 (21.7%)	134 (19.6%)	91 (13.2%)
	Rural, n(%)					
		186 (6%)	74 (11.1%)	42 (6.3%)	20 (3.1%)	12 (1.8%)
	Unknown	146 (4.5%)	25 (3.6%)	28 (4.1%)	45 (6.6%)	30 (4.4%)
Parental Diet/Health	Paternal asthma, n(%)					
		510 (19.2%)	98 (18.7%)	106 (18.5%)	116 (19.7%)	119 (19.5%)
	Unknown	600 (18.4%)	168 (24.3%)	116 (16.8%)	96 (14%)	76 (11.1%)
	Paternal atopy, n(%)					
		1663 (67.7%)	292 (62.7%)	353 (65.6%)	394 (71.1%)	402 (72.3%)
	Unknown	806 (24.7%)	225 (32.6%)	152 (22%)	130 (19%)	131 (19.1%)
	Maternal asthma, n(%)					
		749 (23.4%)	176 (25.7%)	153 (22.2%)	163 (23.9%)	150 (21.9%)
	Unknown	56 (1.7%)	6 (0.9%)	2 (0.3%)	1 (0.1%)	1 (0.1%)
	Maternal distress (1y)					
	Median (Range)	12 (0, 40)	14 (0, 40)	12 (0, 32)	11 (0, 36)	10 (0, 32)
	IQR (Q1,Q3)	7, 17	9, 18	8, 17	6, 15	6, 15
	Unknown	685 (21%)	160 (23.2%)	117 (17%)	125 (18.3%)	119 (17.3%)
	Maternal BMI					
	Median (Range)	23.3 (13.9, 56.9)	24.9 (15.6, 56.9)	23.7 (16.4, 54.4)	22.9 (13.9, 43.2)	22.3 (17, 45.8)
	IQR (Q1,Q3)	21, 27	21.5, 30.2	21.5, 27.5	20.8, 25.8	20.5, 24.7
	Unknown	327 (10%)	74 (10.7%)	48 (7%)	57 (8.3%)	69 (10%)
	Maternal UPF diet					
	Median (Range)	46.8 (1.9, 82.9)	50.3 (8.2, 82.9)	46.7 (13.9, 76.5)	45.8 (15.1, 77.1)	44.9 (3.6, 77.3)
	IQR (Q1,Q3)	39.7, 53.8	43.6, 56.9	39.4, 53.7	39.5, 52.1	38.1, 52
	Unknown	291 (8.9%)	49 (7.1%)	32 (4.6%)	47 (6.9%)	42 (6.1%)
	Maternal atopy, n(%)					
		1727 (57.7%)	340 (53.5%)	383 (59%)	377 (59.3%)	400 (63%)
	Unknown	268 (8.2%)	56 (8.1%)	41 (5.9%)	48 (7%)	52 (7.6%)

Table S2. SES factors across all CHILD sites and COPSAC$_{2010}$ cohort, Related to STAR Methods.

CHILD Cohort						COPSAC	
**Variable**	**Cohort**	**Edmonton**	**Toronto**	**Vancouver**	**Winnipeg**	**Variable**	**Copenhagen**
No. patients	3263	768	768	735	992	No. patients	700
Annual household income (CAD), n(%)						Household income (DKK) covering last 3 months, n(%)	
0-49999	399 (14.1%)	82 (12.1%)	38 (5.7%)	73 (10.8%)	206 (25.2%)	0-150.000	237 (33.9%)
50000-99999	943 (33.2%)	239 (35.2%)	130 (19.6%)	219 (32.3%)	355 (43.5%)	150.000-200.000	200 (28.6%)
100000-149999	795 (28%)	214 (31.5%)	183 (27.6%)	209 (30.8%)	189 (23.1%)	200.000-250.000	157 (22.4%)
>=150000	700 (24.7%)	144 (21.2%)	311 (47%)	178 (26.2%)	67 (8.2%)	>250.000	106 (15.1%)
Unknown	426 (13.1%)	89 (11.6%)	106 (13.8%)	56 (7.6%)	175 (17.6%)	Unknown	0 (0%)
Education of mother, n(%)						Education of mother, n(%)	
High school	276 (8.8%)	63 (8.7%)	24 (3.2%)	21 (2.9%)	168 (18.1%)	College or High school	55 (7.9%)
College	905 (28.9%)	281 (38.7%)	143 (19%)	164 (22.7%)	317 (34.1%)	Tradesman	130 (18.6%)
University	1353 (43.2%)	303 (41.7%)	379 (50.3%)	334 (46.2%)	337 (36.3%)	Medium academic	321 (45.9%)
Master or Phd	598 (19.1%)	80 (11%)	207 (27.5%)	204 (28.2%)	107 (11.5%)	University	194 (27.7%)
Unknown	131 (4%)	41 (5.3%)	15 (2%)	12 (1.6%)	63 (6.4%)	Unknown	0 (0%)
Education of father, n(%)						Education of father, n(%)	
High school	475 (15.3%)	126 (17.5%)	52 (7%)	43 (6%)	254 (27.5%)	College or High school	71 (10.4%)
College	1038 (33.5%)	321 (44.6%)	172 (23.1%)	216 (30.2%)	329 (35.6%)	Tradesman	213 (31.3%)
University	1096 (35.3%)	203 (28.2%)	335 (45%)	290 (40.6%)	268 (29%)	Medium academic	206 (30.2%)
Master or Phd	494 (15.9%)	70 (9.7%)	185 (24.9%)	166 (23.2%)	73 (7.9%)	University	191 (28%)
Unknown	160 (4.9%)	48 (6.2%)	24 (3.1%)	20 (2.7%)	68 (6.9%)	Unknown	19 (2.7%)
Social status (canada)							
Median (range)	7 (1, 10)	7 (1, 10)	7 (1, 10)	7 (1, 10)	6 (1, 10)		
IQR (Q1,Q3)	6, 8	6, 8	7, 8	6, 8	5, 7		
Unknown	190 (5.8%)	53 (6.9%)	31 (4%)	15 (2%)	91 (9.2%)		
Social status (community)						Data not collected	
Median (range)	7 (1, 10)	7 (1, 10)	7 (1, 10)	7 (1, 10)	7 (1, 10)		
IQR (Q1,Q3)	6, 8	6, 8	6, 8	6, 8	5, 8		
Unknown	193 (5.9%)	51 (6.6%)	35 (4.6%)	15 (2%)	92 (9.3%)		
SES index						SES index	
Median (range)	0.1 (-2.4, 1.6)	-0.1 (-2.4, 1.3)	0.4 (-1.9, 1.6)	0.2 (-2, 1.6)	-0.2 (-2.3, 1.3)	Median (range)	0 (-1.3, 1)
IQR (Q1,Q3)	-0.4, 0.4	-0.5, 0.3	0, 0.7	-0.2, 0.6	-0.7, 0.2	IQR (Q1,Q3)	-0.4, 0.4
Unknown	511 (15.7%)	108 (14.1%)	132 (17.2%)	65 (8.8%)	206 (20.8%)	Unknown	19 (2.7%)

Table S3. Prevalence of *Bifidobacterium* species in children breastfed up to 3 months across CHILD study sites (presence at either 3-month or 1-year visit) and $COPSAC_{2010}$ cohort (presence at 1-month or 1-year visit) , Related to Figure 6. q-value is calculated based on chi-square test and with adjustment of multiple comparisons using Benjamini-Hochberg (BH) method.

Species	Vancouver	Toronto	Winnipeg	Edmonton	q-value across CHILD study sites	CHILD Study	$COPSAC_{2010}$ Cohort	q-value between CHILD study and $COPSAC_{2010}$ cohort
Bifidobacterium adolescentis	28%	24%	33%	29%	0.12	29%	83%	<0.001
Bifidobacterium animalis	44%	51%	47%	48%	0.47	47%	<10%	<0.001
Bifidobacterium bifidum	48%	40%	50%	45%	0.12	46%	89%	<0.001
Bifidobacterium breve	66%	60%	56%	63%	0.12	61%	94%	<0.001
Bifidobacterium dentium	36%	40%	39%	34%	0.47	38%	38%	0.94
Bifidobacterium infantis	37%	30%	23%	17%	<0.001	28%	75%	<0.001
Bifidobacterium longum	89%	87%	87%	89%	0.94	88%	100%	<0.001
Bifidobacterium pseudocatenulatum	31%	34%	36%	38%	0.47	35%	86%	<0.001

Table S4. Prevalence and mean relative abundance of *B. infantis* across CHILD and COPSAC cohort, Related to Figure 6.

Cohort	Visit	Sample Size (n)	*B. infantis* prevalence (%)	Mean relative abundance (all samples)	Mean relative abundance (*B. infantis*–positive children)
CHILD	3 months	1332	12.8%	0.097	0.753
	1 year	1426	19.1%	0.050	0.261
COPSAC	1 month	598	29.8%	0.0542	0.182
	1 year	645	54.7%	0.0068	0.012

Table S5. Proportion of missing across SES quartiles for NCD risk factors in CHILD study, Related to STAR Methods. P-value was estimated using fisher's exact test.

Health Outcomes at 5 years of age	Cohort	SES Q_1 (0-25%)	SES Q_2 (25-50%)	SES Q_3 (50-75%)	SES Q_4 (75-100%)	P-value
No. patients	3263	691	690	684	687	
Atopic sensitization at 5y, n(%)						0.051
Missing	708 (21.7%)	151 (21.9%)	125 (18.1%)	128 (18.7%)	160 (23.3%)	
Not missing	2555 (78.3%)	540 (78.1%)	565 (81.9%)	556 (81.3%)	527 (76.7%)	
Asthma at 5y, n(%)						0.053
Missing	864 (26.5%)	199 (28.8%)	167 (24.2%)	154 (22.5%)	171 (24.9%)	
Not missing	2399 (73.5%)	492 (71.2%)	523 (75.8%)	530 (77.5%)	516 (75.1%)	
Overweight/obesity at 5y, n(%)						0.047
Missing	638 (19.6%)	142 (20.5%)	109 (15.8%)	113 (16.5%)	137 (19.9%)	
Not missing	2625 (80.4%)	549 (79.5%)	581 (84.2%)	571 (83.5%)	550 (80.1%)	
Emotional/behavioral problem at 5y, n(%)						0.00059
Missing	903 (27.7%)	215 (31.1%)	153 (22.2%)	168 (24.6%)	159 (23.1%)	
Not missing	2360 (72.3%)	476 (68.9%)	537 (77.8%)	516 (75.4%)	528 (76.9%)	
